# The borylamino-diborata-allyl anion†

**DOI:** 10.1039/d4sc01953a

**Published:** 2024-04-30

**Authors:** Henry T. W. Shere, Han-Ying Liu, Samuel E. Neale, Michael S. Hill, Mary F. Mahon, Claire L. McMullin

**Affiliations:** a Department of Chemistry, University of Bath Claverton Down Bath BA2 7AY UK msh27@bath.ac.uk cm2025@bath.ac.uk

## Abstract

Reactions of β-diketiminato alkaline earth alkyldiboranate derivatives [(BDI)Ae{pinBB(R)pin}] (BDI = HC{(Me)CNDipp}_2_; Dipp = 2,6-i-Pr_2_C_6_H_3_; Ae = Mg, R = *n*-Bu or Ae = Ca, R = *n*-hexyl) with *t*-BuNC provide access to the respective group 2 derivatives of unprecedented diborata-allyl, {(pinB)_2_CNBpin(*t*-Bu)}^−^, anions. Although the necessary mode of B–C bond cleavage implicated in these transformations could not be elucidated, further studies of the reactivity of magnesium triboranates toward isonitriles delivered a more general and rational synthetic access to analogous anionic moieties. Extending this latter reactivity to a less symmetric triboranate variant also provided an isomeric Mg–C-bonded dibora-alkyl species and sufficient experimental insight to prompt theoretical evaluation of this reactivity. DFT calculations, thus, support a reaction pathway predicated on initial RNC attack at a peripheral boron centre and the intermediacy of such dibora-alkyl intermediates.

## Abstract 2

Reactions of alkaline earth alkyldiboranate and triboranate derivatives with organic isonitriles provides access to unprecedented diborata-allyl anions.

## Graphical abstract

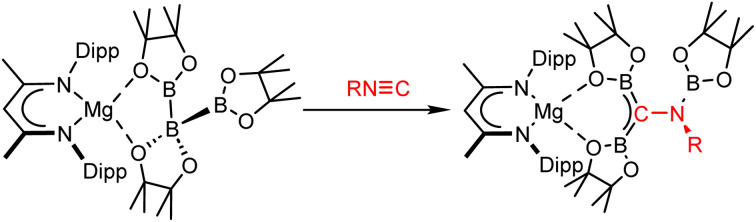

## Introduction

The isolobal replacement of carbon in an organic structure by an alternative p-block element has inspired many fundamental advances in main group chemistry.^1–6^ As the group 13 element with the closest periodic proximity to carbon, the pursuit of multiply bonded, delocalised or heteroaromatic species has been nowhere more prevalent than in the chemistry of boron. Since, for example, the initial characterisation of B=C bonding by, among others, Berndt,^7,8^ Nöth,^9^ Paetzold^10,11^ and Power,^12^ boron has been incorporated into a wide variety of homo- and heteronuclear multiply bonded compounds.^13–15^

**Fig. 1:**
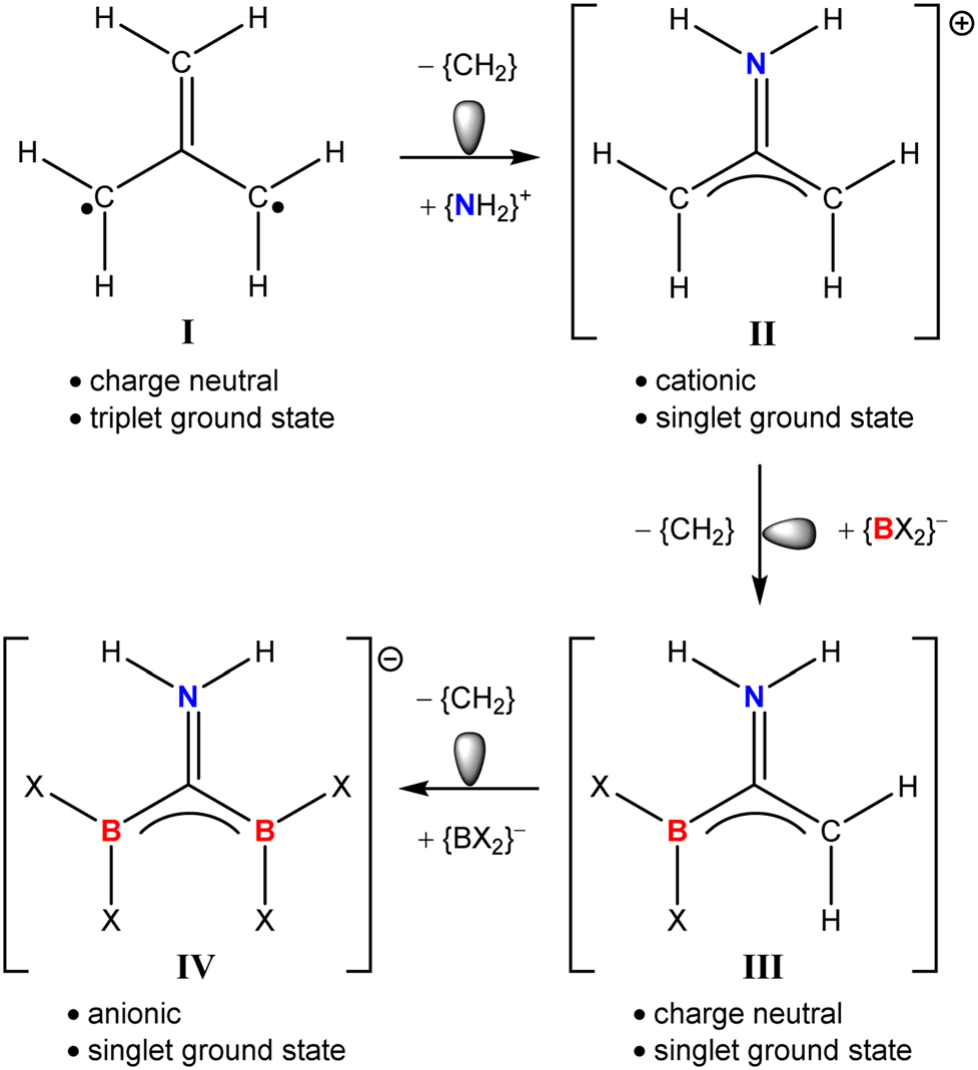
Isolobal progression from the triplet diradical ground state of (one resonance hybrid of) trimethylenemethane (I) to the hypothetical singlet species II, III and IV.

While the incorporation of planar (sp^2^) boron into the structures of aromatic heterocycles and polycyclic aromatic hydrocarbons (PAHs) continues as an area of recent topicality,^16–20^ similar modification of many fundamental organic moieties remains to be achieved. A case in point is trimethylenemethane (I), the *D*_3h_ structure of which, though conjugated, represents a non-Kekulé hydrocarbon in that no resonance structure allows its four available electrons to be distributed across three C–C π bonds (Fig. 1).^21–23^ Analysis of the frontier molecular orbitals of I identifies its HOMO as two degenerate half-filled (*e*′′) π-symmetric wavefunctions, such that Hund's rule dictates its ground state as a triplet diradical.^24,25^ Frontier MO degeneracy is lost, however, by any lowering of molecular symmetry, either by Jahn–Teller distortion or hetero-replacement of one of its carbon atoms.^26^ The lower (*C*_2v_) symmetry of the iminiumdimethylenemethane cation (II), for example, in which a single methylene of I is substituted by (NH_2_)^+^ provides a now closed shell singlet species with a fully occupied (b_1_) HOMO. While II and related wholly organic species have attracted some experimental and theoretical scrutiny,^25,27–29^ boron-centred substitution of I has evaded consideration. Isolobal progression from II, for example, may be envisaged by sequential replacement of methylene by {BX_2_}^−^ (where X represents any sigma-bonded heteroelement) to provide the respective hypothetical neutral and anionic analogues, III and IV.

**Scheme 1:**
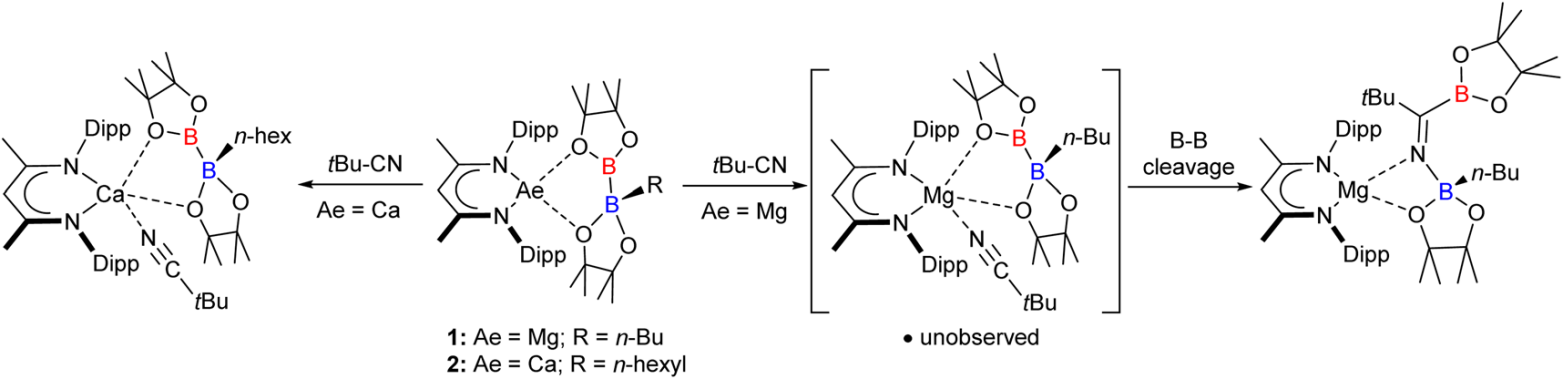
Previously reported reactivity of compounds 1 and 2 toward *t*-BuCN.

The current work arises from our study of the β-diketiminato (BDI = HC{(Me)CNDipp}_2_; Dipp = 2,6-i-Pr_2_C_6_H_3_) magnesium and calcium alkyl diboranate derivatives, compounds 1 and 2 (Scheme 1),^30,31^ which are obtained by reaction of bis(pinacolato)diboron (B_2_pin_2_) with the relevant alkaline earth alkyls.^32–35^ In common with other systems resulting in a desymmetrization of B_2_pin_2_,^36–38^ compound 1 behaves as a surrogate source of the [Bpin]^−^ anion enabling B–C bond formation when treated, for example, with *t*-BuCN (Scheme 1).^39^ Analysis of this reactivity by density functional theory (DFT) indicated that B–B′ heterolysis is induced by (‘inner sphere’) coordination at magnesium, rather than (‘outer sphere’) interaction with the trigonal boron of the diboranate anion.^31^ Although reaction of 2 with *t*-BuCN resulted in similar ‘inner sphere’ coordination to the group 2 centre, the resultant calcium nitrile adduct was stable to onward transformation, behaviour rationalised to result from the greater ability of the larger alkaline earth cation to support a 5-coordinate geometry and a consequent inability to displace RBpin (Scheme 1).^31^

Diverse insertion and bond activation reactivity has also been observed when either boron–boron or metal–boron bonded compounds are treated with isonitriles.^40–49^ In this contribution, therefore, we report that compounds 1 and 2 react with the isonitrile isomer of *t*-BuCN, *t*-BuNC, to provide an unusual diborata-allyl anion, which may also be considered as an isoelectronic (albeit non-isolobal) analogue of trimethylenemethane (see IV, Fig. 1). Although the initial identification of this reactivity was serendipitous, we further report that a more rational and general synthesis of such anions may be achieved by use of magnesium triboranate derivatives, a protocol which is also amenable to more confident theoretical (DFT) analysis.

## Results and discussion

### Group 2 diboranate/isonitrile reactivity

Addition of *t*-BuNC to a *d*_8_-toluene solution of 1 initiated an immediate colour change from colourless to orange. Monitoring by ^1^H NMR spectroscopy at room temperature revealed a gradual decrease in intensity of the BDI γ-methine resonance of 1 (*δ* 4.79 ppm), culminating in its complete disappearance after *ca.* 14 hours. This transformation occurred with a further colour change of the solution to dark green and the concurrent emergence of two predominant BDI γ-methine ^1^H NMR singlet signals at *δ* 4.91 and 4.83 ppm, which resonated with an approximate 1 : 0.6 ratio of intensities. The corresponding ^11^B{^1^H} NMR spectrum comprised three signals at *δ* 34.3, 25.2 and 7.3 ppm, consistent with the presence of two similar but discriminated 3-coordinate boron nuclei and a 4-coordinate boron environment, respectively.^50^ An analogous reaction performed between 2 and *t*-BuNC also induced an instantaneous colour change from amber to dark brown. Although a ^1^H NMR spectrum recorded after 45 minutes indicated a more complex reaction and the presence of at least five new BDI ligand environments, the resultant ^11^B{^1^H} spectrum was reminiscent of that provided by the magnesium-based reaction and again indicative of a mixture of three- (*δ* 34.2, 31.2 ppm) and four-coordinate (*δ* 7.7 ppm) boron environments.

**Scheme 2:**
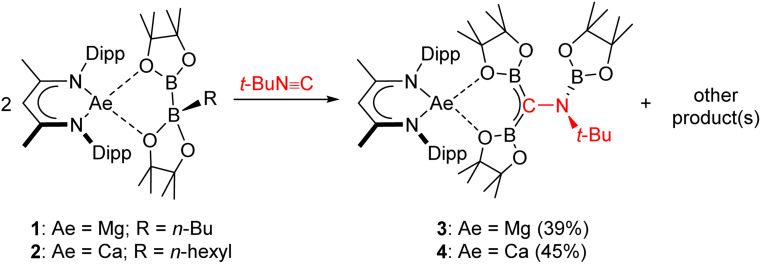
Synthesis of compounds 3 and 4.

Fractional crystallisation of both crude reaction mixtures from mixed toluene/*n*-hexane solvent systems allowed the separation of compounds 3 and 4, which were isolated in yields of 39 and 45% from the respective magnesium- and calcium-based reactions (Scheme 2). Analysis by ^1^H NMR spectroscopy of both crystalline samples now indicated the presence of single magnesium- (3, γ-methine *δ* 4.91 ppm) and calcium-bound (4, γ-methine *δ* 4.70 ppm) BDI ligand environments, while the ^11^B{^1^H} spectra displayed signals consistent with, in each case, the presence of two discriminated three-coordinate boron nuclei [3: *δ* 34.2, 25.2; 4: 34.3, 25.5 ppm].

**Fig. 2:**
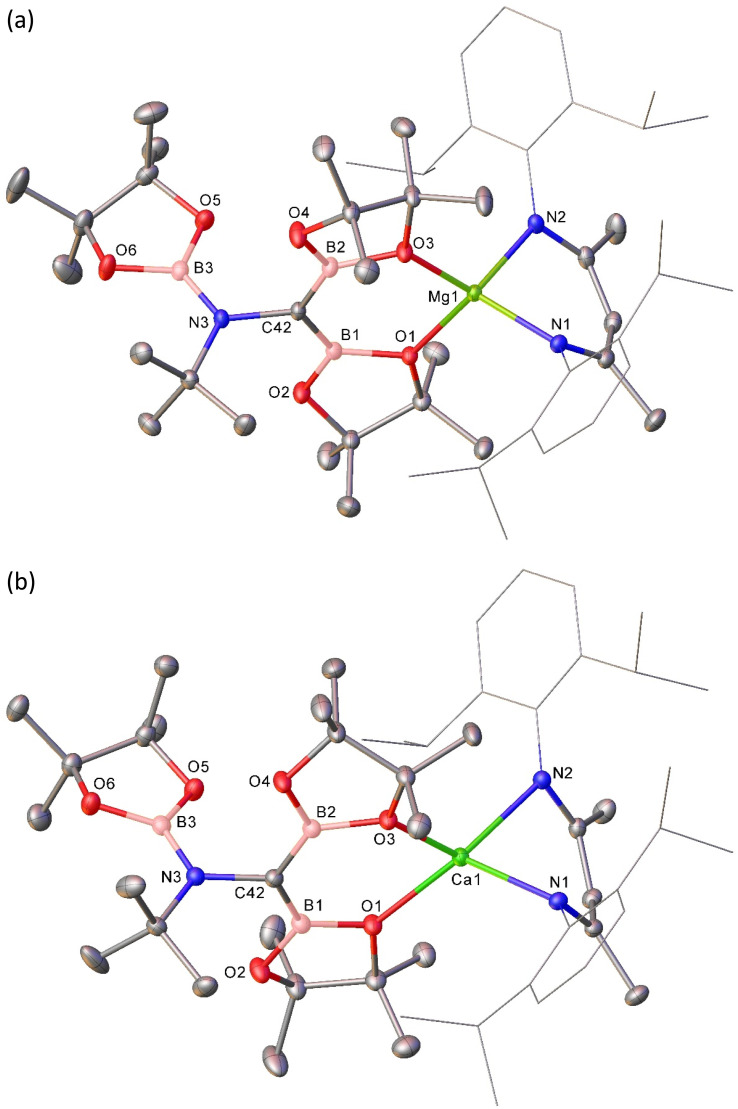
Molecular structures of (a) compound 3 and (b) compound 4. Ellipsoids displayed at 30% probability. Solvent, hydrogen atoms and minor disordered components have been omitted for clarity. Dipp substituents are displayed as wireframes, also for visual ease.

The origin of these observations was resolved by single crystal X-ray diffraction analysis performed on both 3 and 4, the results of which are shown in Fig. 2 with selected bond lengths and angle data presented in Table 1. Although not isostructural, both compounds present connectivities that differ only in the identity of the group 2 metal centre. Any minor variations in the comparable metric data may, thus, be attributed to the differing radii of the alkaline earth cations.^51^ In both cases the pseudo-tetrahedral geometries of the BDI-chelated group 2 cations are completed by *O*,*O*-coordination of a borylamino-diborata-allyl anion. The central (C42) carbon atoms can be identified as arising from the terminal carbon of the *t*-BuNC reagent through their maintenance of the N3–C42 bonds, while N3 is further bound by a third [Bpin] moiety. The B–C42 bonds in both compounds [3: B1–C42 1.467(2), B2–C42 1.477(2); 4: B1–C42 1.4888(18), B2–C42 1.4786 Å] are significantly shorter than is typical for a B–C single bond to three-coordinate boron (*ca*. 1.58–1.62 Å).^12^ These distances, thus, lie within the range established for interactions which incorporate a significant degree of B–C π bonding such as in [Mes_2_BCH_2_]^−^ (1.444(8) Å),^12^ 8,10,11a-trimethyl-7-mesityl-11a*H*-7-boratabenzo[de]anthracene (1.48 Å)^52^ and various boratabenzene-type molecules.^53,54^ B1, C42 and B2 adopt trigonal planar geometries in both structures, with sums of angles subtended about C42 of 360°. While the {CB_2_} units also adopt coplanar orientations with respect to N3, the least squares planes defined by [N3–C42–B1–O1–Ae1–O3–B2] and [B3–N3–C43] are effectively orthogonal in both structures (3, Ae = Mg: 88.14; 4, Ae = Ca 88.08°). Although the N3 atoms are also trigonal, the absence of π conjugation of these centres with C42 is evident in the N3–C42 bond lengths (3: 1.4816(19); 4: 1.4815(15) Å], which are only marginally shorter than the bonds between N3 and their *tert*-butyl substituents [3: 1.498(2); 4: 1.4865(16) Å] and, thus, consistent with reduction of the isonitrile N≡C interaction [1.184 Å] to a single bond.^55^ Although the {NCB_2_} sigma frameworks of the complex anion cores are isoelectronic with the anionic skeleton of IV (Fig. 1), the forfeit of extended π-conjugation across all four atoms dictates that their description as complete dimethylenemethane isosteres would be incorrect. Rather, the still delocalised units are better rationalised as diborata-allyl anions, each bearing a pendent borylamino {N(*t*-Bu)(Bpin)} substituent.

**Table 1:** Selected bond distances (Å) and angles (°) for compounds 3, 4, 7 and 8

	3^a^	4^b^	7^a^	8^a^
M1–O1	2.0577(8)	2.2927(9)	2.0454(9)	2.0642(11)^c^
M1–O3	1.9811(8)	2.2487(9)	1.9700(9)	2.2254(14)^d^
M1–N1	2.0657(10)	2.3153(11)	2.0411(11)	2.0654(13)
M1–N2	2.0630(10)	2.3125(10)	2.0607(11)	2.0894(12)
B1–C42	1.4758(16)	1.4888(18)	1.4800(18)	1.535(2)^e^
B2–C42	1.4642(16)	1.4786(18)	1.4726(17)	1.530(2)^f^
N3–C42	1.4805(13)	1.4815(15)	1.4785(15)	1.4966(17)^g^
N3–B3	1.3934(16)	1.3933(19)	1.3988(17)	1.394(2)
O1–M1–O3	99.03(3)	92.04(3)	97.82(4)	80.87(5)^h^
N1–M1–N2	93.52(4)	84.13(4)	93.87(4)	93.13(5)
B1–C42–B2	127.25(10)	130.84(12)	126.84(11)	114.12(12)^i^
B1–C42–N3	116.55(9)	113.10(11)	115.40(10)	115.26(12)^j^
B2–C42–N3	116.15(9)	115.46(10)	117.63(10)	118.89(12)^k^

a M1 = Mg.

b M1 = Ca.

c Mg1–O5.

d Mg1–C36.

e B1–C36.

f B2–C36.

g N3–C36.

h O5–Mg1–C36.

i B1–C42–B2.

j B1–C36–N3.

k B1–C36–N3.

**Fig. 3:**
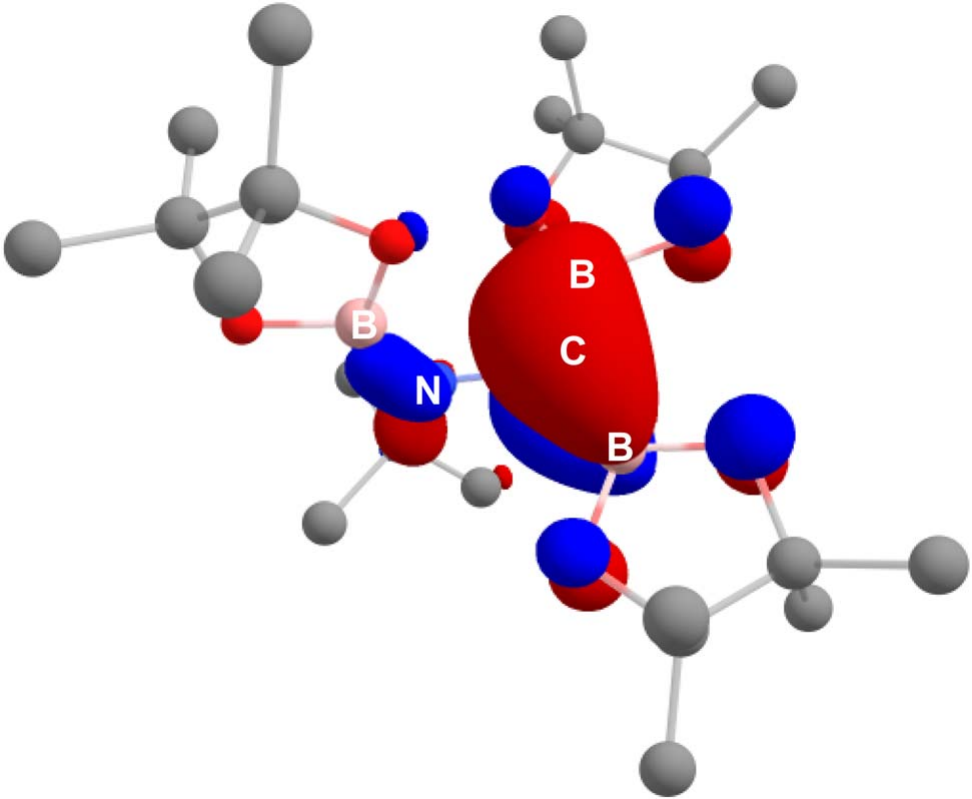
HOMO of the borylamino-diborata-allyl anion, which predominantly features B–C–B π-bonding character, computed at the BP86-D3BJ/6-311++G**//BP86/BS1 level of theory.

This interpretation of the bonding within the borylamino-diborata-allyl moieties of compounds 3 and 4 was validated by MO analysis of both compound 3 and the non-complexed anion itself. In both cases, the HOMO was identified as a molecular orbital comprising delocalised B–C–B π-bonding (Fig. 3 and S25†), with Wiberg Bond Indices (WBIs) of *ca*. 1.2 for each B–C bond and 0.94 for the N–C interaction across the {NCB_2_} core of the anion.

As discussed above, inspection of the initial ^1^H and ^11^B{^1^H} NMR spectra of both reactions leading to the isolation of 3 and 4 evidenced the formation of additional products. Although no definitive identification of any other compounds could be achieved, we tentatively suggest that the concurrent generation of alkaline earth dialkylborate, [R_2_Bpin]^−^, species provides a plausible rationalisation of the reaction mass balance and, specifically, the 4-coordinate boron environments apparent in the initially recorded ^11^B{^1^H} NMR spectra. The characterisation of compounds 3 and 4 and isolated yields approaching 50% are also suggestive of reactions that approximate to a supposed 1 : 1 stoichiometry with such dialkylborate co-products. Although we decline to assign a compound number to these proposed species, previous observations of similar dismutation of HBpin to [H_2_Bpin]^−^ in a number of magnesium-^56–60^ and calcium-based^61^ systems provides tentative support for this hypothesis.

### Magnesium triboranate/isonitrile reactivity

**Scheme 3:**
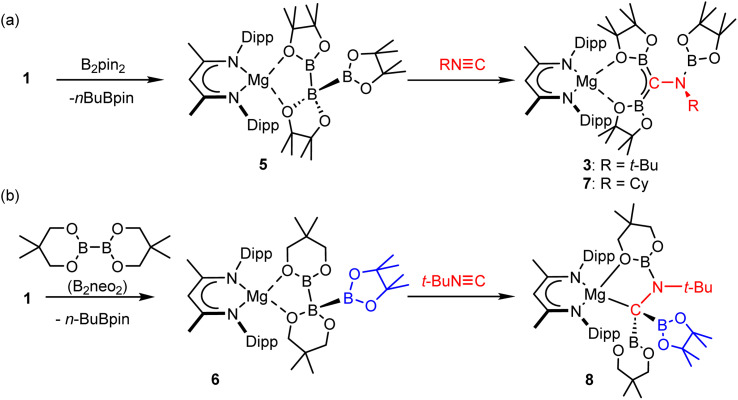
(a) Synthesis of compounds 3 and 7*via* compound 5; (b) synthesis of compound 8*via* compound 6.

We cannot with any confidence identify the respective mode of formation of compounds 3 and 4 from compounds 1 and 2. The mass balance associated with the formation of their constituent borylamino-diborata-allyl anions, however, advocates the necessary delivery of three {Bpin} moieties to each *tert*-butyl isonitrile. We have previously reported that treatment of compound 1 with a further stoichiometric equivalent of B_2_pin_2_ (or an alternative diborane ester, *vide infra*) induces the elimination of *n*BuBpin and the formation of magnesium species such as 5 and 6, comprising unusual catena-B–B–B anions (Scheme 3).^30,39^ Although related processes have also been observed for magnesium's heavier group 2 congeners, the potential utility of the resultant species was found to be limited by facile Schlenk-type equilibration of the resultant compounds.^31^

Accordingly, a reaction was performed between compound 5 and *t*-BuNC (Scheme 3a). This procedure provided a single solution species that presented ^1^H, ^13^C{^1^H} and ^11^B{^1^H} NMR spectra identical to those of a pure sample of compound 3. Furthermore, crystallisation from the reaction solution enabled the isolation of a high (>70%) yield of compound 3, the formation of which was further corroborated by a unit cell check performed on a single crystal.

With this rational route to compound 3 in hand, the generality of this transformation was assessed by treatment of compound 5 with cyclohexyl isonitrile (Scheme 3a). This procedure yielded selective conversion to a single β-diketiminato product, compound 7, which presented NMR spectra suggestive of a solution symmetry strongly reminiscent of that of compound 3. The supposed generation of a *N*-cyclohexyl substituted variant of the borylamino-diborata-allyl anion was confirmed by the isolation of compound 7 as colourless single crystals (66%) and subsequent X-ray diffraction analysis.

**Fig. 4:**
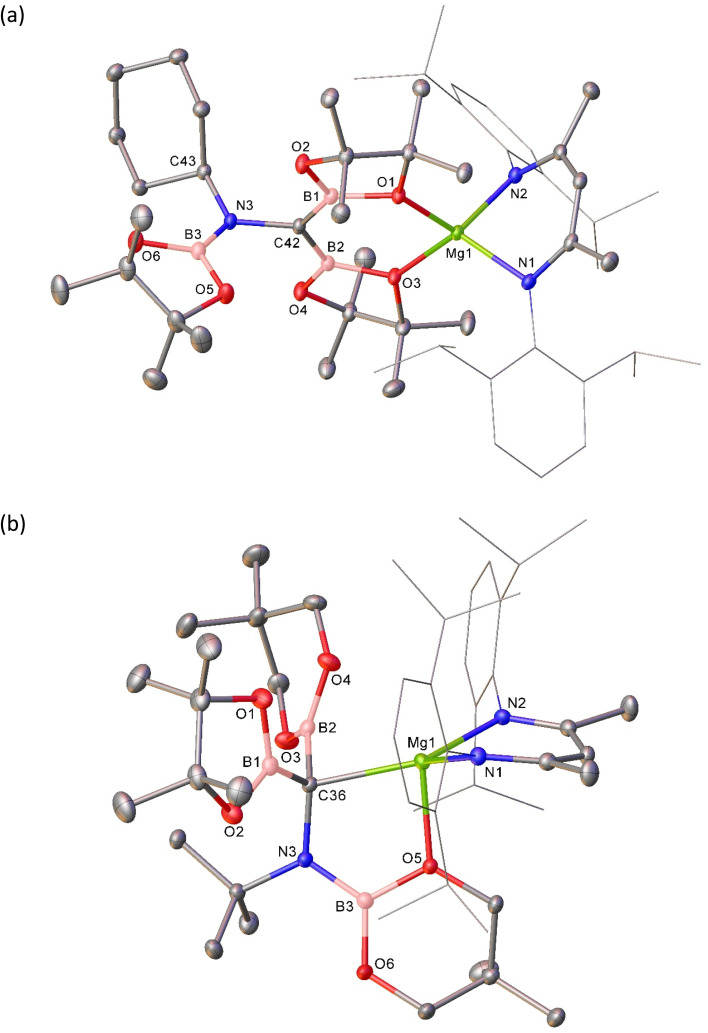
Molecular structures of (a) compound 7 and (b) compound 8. Ellipsoids displayed at 30% probability. Solvent and hydrogen atoms have been omitted for clarity. Dipp substituents are displayed as wireframes, also for visual ease.

Although the close comparison of the solid-state structure of 7 (Fig. 4a and Table 1) to that of compound 3 obviates further comment, the selective transformation of compound 5 and its delivery of three equivalents of the {Bpin} unit to both compounds 3 and 7 prompts consideration of a number of mechanistic possibilities. We have previously suggested that B–B′ heterolysis in molecules such as 1 and 2 may be induced either by (‘inner sphere’) interaction of a basic substrate molecule with the alkali metal centre itself or (‘outer sphere’) approach to the 3-coordinate boron atom of the {pinBB(R)pin}^−^ anion.^30,62–65^ While we have previously observed some structural evidence for these contrasting possibilities, in the current context, the identical constitution of all three {Bpin} components of the triboranate anion of 5 does not allow regiochemical discrimination of their delivery to the isonitrile N≡C bond. We have, however, also previously reported that reaction of compound 1 with the alternative diborane(4) ester, 5,5,5′,5′-tetramethyl-2,2′-bi(1,3,2-dioxaborinane) (B_2_neo_2_) provides compound 6 through selective formation of the similarly homocatenated but now asymmetrically-borylated {(Bneo)B(neo)(Bpin)}^−^ anion (Scheme 3b).

Reasoning this latter moiety would provide a means for discrimination between the reactivity of the {Bneo} and {Bpin} components, compound 6 was treated with an equimolar quantity of *t*-BuNC. Although the complexity of the ^1^H NMR spectrum provided by the resultant orange solution provided only limited insight into the outcome of the reaction, it was clearly evident that a single new β-diketiminato magnesium species (8; BDI γ-methine *δ*_H_ 4.82 ppm) had been formed (Scheme 3b). Single crystals of compound 8 were obtained by slow evaporation of the benzene reaction solution and its identification was achieved by X-ray diffraction analysis (Fig. 4b and Table 1).

The three boron ester functions of compound 6 again result in the generation of two B–C bonds and a single B–N bond such that the isonitrile-derived moiety is constitutionally analogous to those observed in all three compounds, 3, 4 and 7. The newly formed anion of compound 8, however, adopts a contrasting configuration and a different mode of coordination to the alkaline earth centre. While still bidentate, engagement with Mg1 is now achieved through coordination by the tetravalent C36 atom and O5, a constituent of a nitrogen-bound {Bneo} moiety. Although the Mg1–C36 bond length [2.2254(14) Å] is elongated in comparison to typical Mg–C_alkyl_ interactions in which magnesium is similarly 4-coordinate (*ca*. 2.14–2.15 Å), the pyramidalisation at C36 indicates that compound 8 is better considered as a σ-bonded organometallic derivative. This latter inference is also supported by the C36–B1 [1.535(2) Å] and C36–B2 [1.530(2) Å] bonds, both of which are significantly longer than the analogous C42–B1/2 interactions (*ca.* 1.47 Å) observed in compounds 3, 4 and 7 (Table 1). While this observation is consistent with the loss of π-conjugation attributed to these latter bonds, of more general significance is the dissimilar identity of the two C-bonded boron ester units of 8, which have resulted from the installation of the unique {Bpin} and a single {Bneo} component of the {(Bneo)B(neo)(Bpin)}^−^ anion of 5. Any mechanistic hypothesis must, therefore, account for the notable regioselectivity of this outcome.

**Fig. 5:**
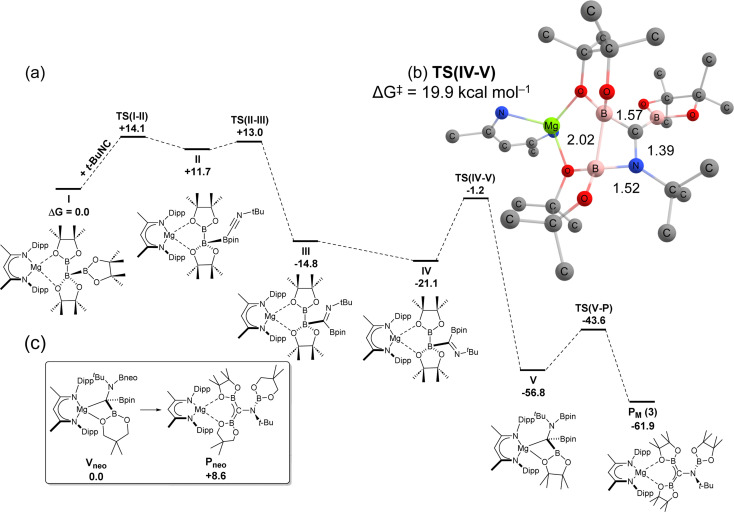
(a) Free energy profile (calculated with DFT at the BP86-D3BJ(PCM = toluene)/BS2//BP86/BS1 level of theory, energies in kcal mol^−1^) of ^*t*^BuNC to I to form P_M_. (b) Structure of rate-limiting TS(IV-V) with selected interatomic distances in Å. (c) Relative difference in free energies between V_neo_ (crystallographically observed) and P_neo_.

DFT calculations were employed (BP86-D3,PCM = toluene/BS2//BP86/BS1 level) to interrogate the mechanism of formation of diborata-allylmagnesium adduct P_M_ (3) through addition of *t*-BuNC to triboratomagnesium species I (Fig. 5). The steric demands of the triboranate anion of I ensure that initial interaction with the bulky isonitrile, *t*-BuNC, takes place *via* coordination to the outermost {Bpin} in the catenated triboron [B_3_pin_3_]^−^ group, *via*TS(I-II) (+14.1 kcal mol^−1^) to form II (+11.7 kcal mol^−1^). Subsequent B–B cleavage, by what is now effective carbene insertion, takes place *via*TS(II-III) (+13.0 kcal mol^−1^) to form a 1,1-dipinacolboratoimine species, III. Onwards torsion rotation of the imine group about the B–C bond to form IV,^66^ precedes a rate-limiting B–N coupling process *via* a four-membered B–B–N–C geometry (TS(IV-V), −1.2 kcal mol^−1^). This transition state structure, with an energetic span of +19.9 kcal mol^−1^ relative to IV, invokes concomitant B–B bond cleavage whilst forming V (−56.8 kcal mol^−1^), in a process akin to σ-bond metathesis. Finally, a conformational rearrangement takes place *via* a modest barrier (13.2 kcal mol^−1^) at TS(V–P) (−43.6 kcal mol^−1^) to afford the observed borylamino-diborata-allyl species P_M_ at −61.9 kcal mol^−1^.^67^ The structure of compound 8 presents a notable analogy with the computed Mg–C σ-bonded organomagnesium species, V_P_, which is formed en route to P_M_. We, thus, hypothesise that both triboranatomagnesium species undergo similar isonitrile addition mechanisms, whereby the divergence in the ultimate regiochemistry is governed by the thermodynamic favourability of the final structures. To this end, calculation of “V_neo_” (Δ*G* = 0.0 kcal mol^−1^) (8) and its analogous but hypothetical borylamino-diborata-allyl isomer “P_neo_” (Δ*G* = +8.6 kcal mol^−1^) from 6, revealed a computed thermodynamic preference for the pyramidal arrangement in the former species (Fig. 5, inset). This deduction is congruent with experimental observation and supports the hypothesis that the regiodivergent outcomes of the reactions of *t*-BuNC with either 1 or 6 arise from thermodynamic control in the latter stages of the isonitrile addition mechanism.

## Conclusion

Treatment of β-diketiminato alkaline earth alkyldiboranate derivatives with organic isonitriles provides access to unprecedented diborata-allyl anions. Although the mode of B–C bond cleavage necessarily implicated in this reactivity could not be identified, further studies of magnesium triboranates with isonitrile reagents provided analogous anions and sufficient experimental insight to encourage theoretical analysis. DFT calculations, thus, support a reaction pathway predicated on initial RNC attack at a peripheral boron centre and the intermediacy of isomeric dibora-alkyl intermediates akin to compound 8. While the sterically enforced non-coplanarity of the pendent bora-amine moieties of the constituent dibora-allyl anions of compounds 3, 4 and 7 disrupt any potential isolobal relationship to I (Fig. 1), the current observations prompt speculation that the latter species may yet prove accessible with modified substituents. We are, thus, continuing to address this possibility and to assess the broader coordination chemistry of these unusual anions.

## Data availability

Crystallographic data have been deposited with the CCDC under deposition numbers 2298656, 2298657, 2341389 and 2341390 for 3, 4, 7 and 8, respectively. Experimental information, images of NMR spectra and details of the X-ray and computational analyses can be found in the ESI.† Further information is available upon request to the corresponding authors.

## Author contributions

HTWS and H-YL carried out the synthesis and characterisation of all new compounds. The X-ray diffraction studies were overseen and finalised by MFM. CLM and SEN conceived and interpreted the computational investigations. MSH directed the investigation and compiled the manuscript with all authors contributing to the finalised version.

## Conflicts of interest

There are no conflicts to declare.

## Supplementary Material

SC-015-D4SC01953A-s001

SC-015-D4SC01953A-s002
