# Supplementary material for: The borylamino-diborata-allyl anion
Source: Chem Sci. 2024 Apr 30;15(21):7999–8007. doi: 10.1039/d4sc01953a (PMC11134337; doi:10.1039/d4sc01953a)
Supplement: SC-015-D4SC01953A-s001 [file SC-015-D4SC01953A-s001.pdf]

Electronic Supplementary Material (ESI) for Chemical Science.
This journal is © The Royal Society of Chemistry 2024

Supporting Information for

The Borylamino-diborata-allyl Anion

Henry T. W. Shere, Han-Ying Liu, Sam E. Neale, Michael S. Hill,* Mary F. Mahon and Claire L. McMullin*

Department of Chemistry, University of Bath, Claverton Down, Bath, BA2 7AY, UK

Experimental Information

1.1 General information

Except stated otherwise, all the experiments were conducted using standard Schlenk line and/or glovebox techniques under an inert atmosphere of argon. NMR spectra were recorded with an Agilent ProPulse spectrometer (^{1}H at 500 MHz, ^{13}C at 126 MHz, ^{11}B at 160 MHz). The spectra are referenced relative to residual protio solvent resonances. Elemental analyses were performed at Elemental Microanalysis Ltd., Okehampton, Devon, UK. Solvents were dried by passage through a commercially available solvent purification system and stored under argon in ampoules over 4 Å molecular sieves. C_6D_6 and *d*$_8$-Toluene was purchased from Sigma-Aldrich, dried over a potassium mirror before distilling and storage over molecular sieves. [(BDI^{Dipp})Mg{pinB(Bpin*n*Bu)}](**1**)[(BDI^{Dipp})Mg{pinB}$_3$](**5**),[1][(BDI^{Dipp})Ca{pinB(Bpin*n*Bu)}] (**2**),[2] [(BDI^{Dipp})Mg{(neo)BB(neo)Bpin}] (**6**),[3] and were prepared according to reported procedures. All other chemicals were purchased from Merck and used without further purification.

1.2. Synthetic procedures

Synthesis of [(BDI^{Dipp})Mg{(pinB)$_2$-C(N(*t*-Bu)(Bpin))}] **(3)**

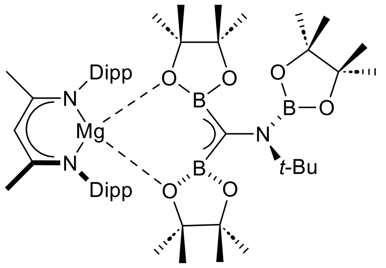

Method A: In a J Youngs NMR tube, d_8-toluene (*ca*. 0.5 mL) was added to compound [(BDI^{Dipp})Mg{pinB(Bpin*n*Bu)}] (**1**, 75.6 mg, 0.10 mmol). *t*-BuNC (2.78 mg, 3.78 μL, 0.03 mmol) was then introduced, which induced a colour change from colourless to orange. This solution was left at room temperature overnight and the toluene was removed *in-vacuo* from the now dark green solution. The resulting solid was redissolved in a mixture of toluene and *n*-hexane. Colourless block crystals (11.7 mg, 39%) deposited at room temperature to provide compound **3**. **Method B:** In a J Youngs NMR tube, *t*-BuNC (8.3 mg, 11.3 μL, 0.10 mmol) was added to a colourless d_6-benzene (*ca*. 0.5 mL) solution of [(BDI^{Dipp})Mg{pinB}$_3$] (**5**, 82.3 mg, 0.10 mmol). The reaction mixture was observed to change into a pale- yellow solution. The reaction mixture was then left at room temperature overnight, and the resulting solution was now orange. Slow evaporation of the benzene solution in the glovebox afforded compound **3** as colourless crystals suitable for X-ray single-crystal diffraction. Yield 64.2 mg, 71%. Anal. Calcd. For $C_{52}H_{87}B_3MgN_3O_6$ (**3**): C, 68.86; H, 9.67; N, 4.63%. Found: C, 69.35; H, 9.59; N, 4.73%. ^{1}H NMR (500 MHz, 298 K, d_8-Tol) δ 7.10 (m, 6H, Ar-*H*), 4.91 (s, 1H, NC(CH$_3$)C*H*), 3.36-3.17 (m, 4H, C*H*(CH$_3$)$_2$), 1.77 (d, 3H, $^3J_{HH}$ = 6.8 Hz, CH(C*H*$_3$)$_2$), 1.68 (s, 6H, C*H*$_3$), 1.67 (s, 3H, NC(C*H*$_3$)CH), 1.61 (s, 3H, NC(C*H*$_3$)CH), 1.52 (d, 3H, $^3J_{HH}$ = 6.8 Hz, CH(C*H*$_3$)$_2$), 1.44 (d, 3H, $^3J_{HH}$ = 6.8 Hz, CH(C*H*$_3$)$_2$), 1.35 (s, 3H, C*H*$_3$), 1.34 (s, 3H, C*H*$_3$), 1.27 (s, 12H, C*H*$_3$), 1.23 (d, 6H, $^3J_{HH}$ = 6.8 Hz, CH(C*H*$_3$)$_2$), 1.21 (s, 3H, C*H*$_3$), 1.20-1.18 (m, 12H, C*H*$_3$), 1.15 (d, 6H, $^3J_{HH}$ = 6.8 Hz, CH(C*H*$_3$)$_2$), 0.71 (s, 3H, C*H*$_3$), 0.69 (s, 3H, C*H*$_3$), 0.60 (s, 3H, C*H*$_3$) ppm. ^{13}C{^{1}H} NMR (126 MHz, 298 K, d_8-Tol) δ 171.9 (N*C*(CH$_3$)CH), 145.8, 145.5, 144.5, 142.6, 142.47, 126.4, 126.3, 125.2, 124.8 (*C* Ar), 97.9 (NC(CH$_3$)*C*H), 88.3, 86.4, 79.8 (B(O*C*(CH$_3$)$_2$)$_2$), 78.3 (B$_2$*C*N), 78.2 (B(O*C*(CH$_3$)$_2$)$_2$), 32.1 (B(OC(*C*H$_3$)$_2$)$_2$), 28.9, 28.9, 28.8, 28.5 (*C*H(CH$_3$)$_2$), 27.1, 27.0, 26.7, 26.5, 26.1, 26.1 (*C*H$_3$), 26.0, 26.0 (NC(*C*H$_3$)CH), 25.9, 25.9, 25.5, 25.5, 25.3, 25.1, 25.1, 24.9, 24.8, 24.4, 24.0 (*C*H$_3$) ppm. ^{11}B{^{1}H} NMR (160 MHz, 298 K, d_8-Tol): δ 34.2, 25.2 ppm.

Figure S1: ^{1}H NMR spectrum (500 MHz, 298 K, d_8-Tol) of **3**.

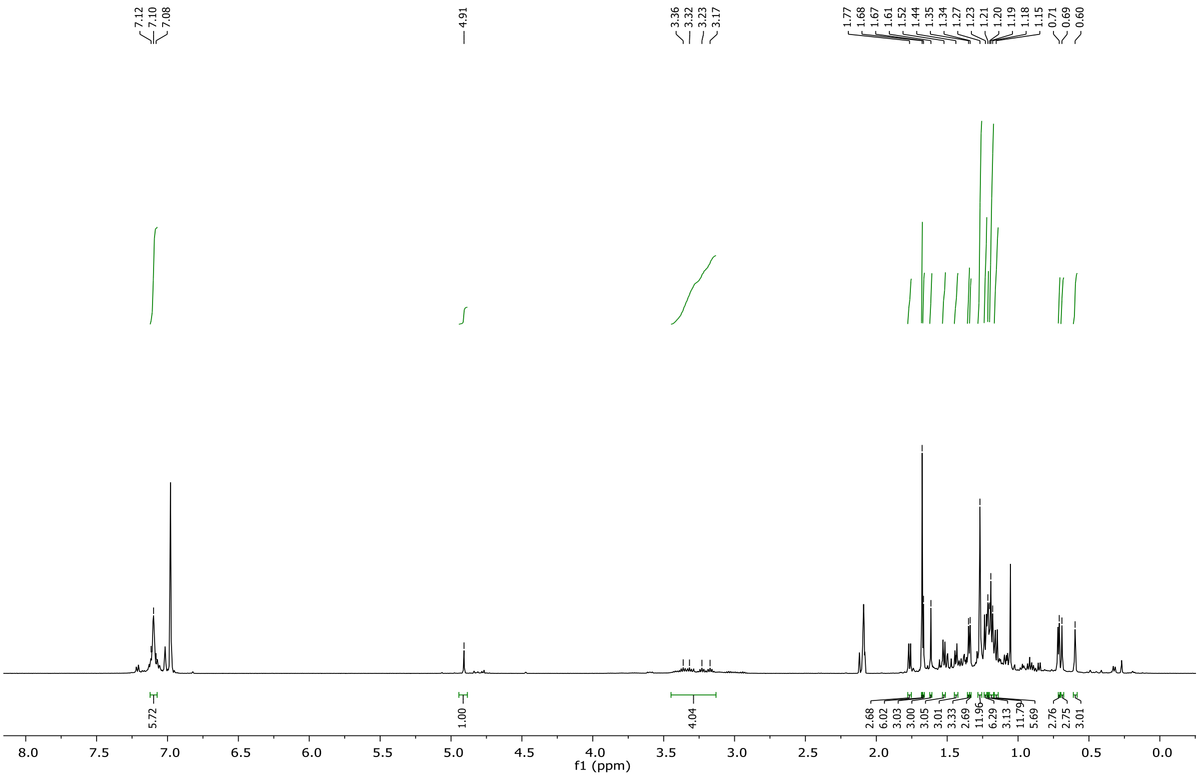

Figure S2: ^{13}C{^{1}H} NMR spectrum (126 MHz, 298 K, d_8-Tol) of **3**.

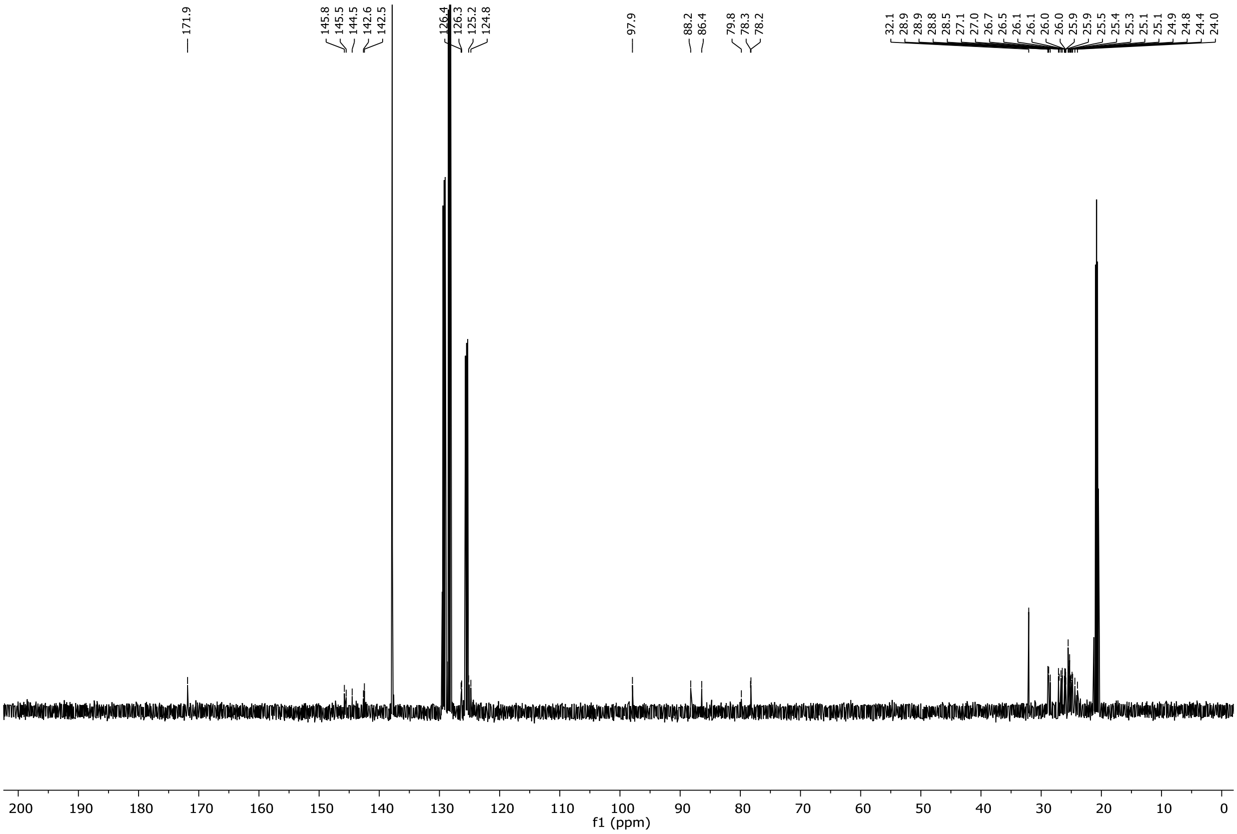

Figure S3: $^{11}B\{^{1}H\}$ NMR spectrum (160 MHz, 298 K, d_8-Tol) of **3**.

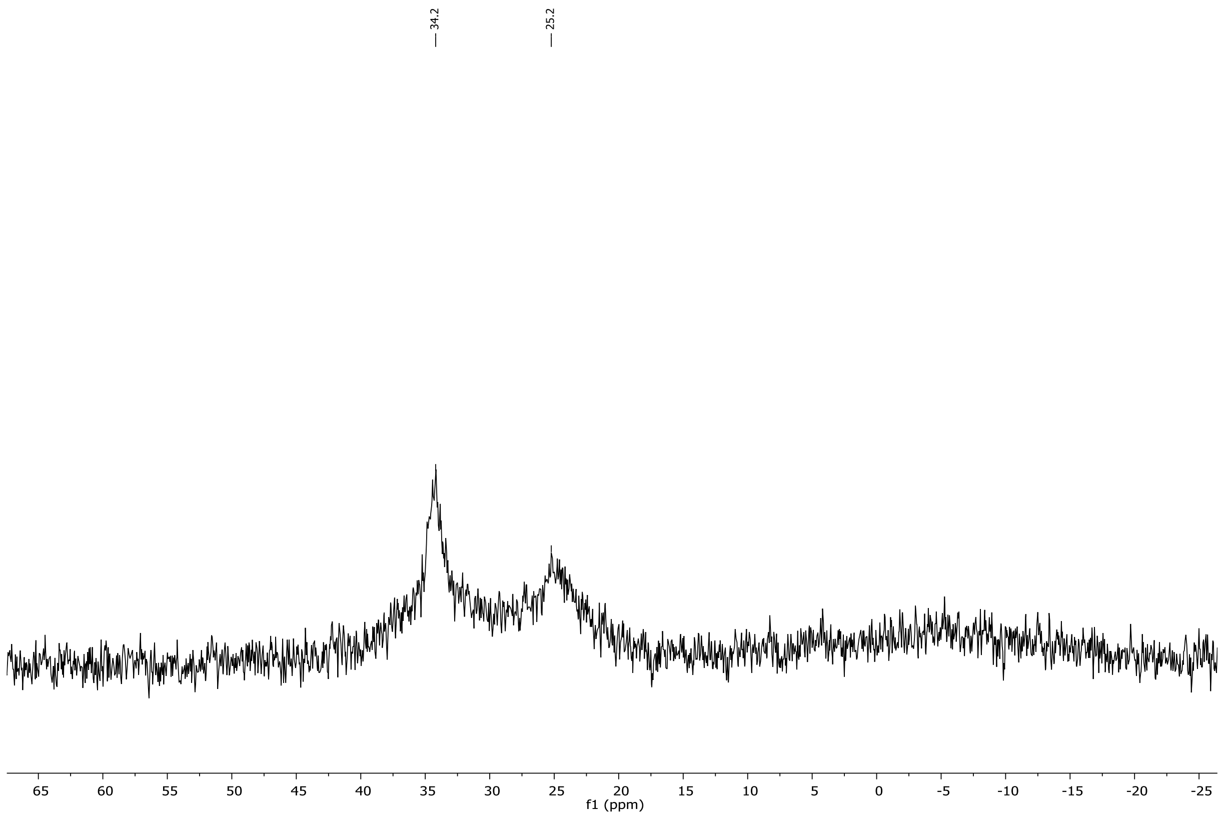

Figure S4: ^{1}H-^{1}H COSY trace of **3**.

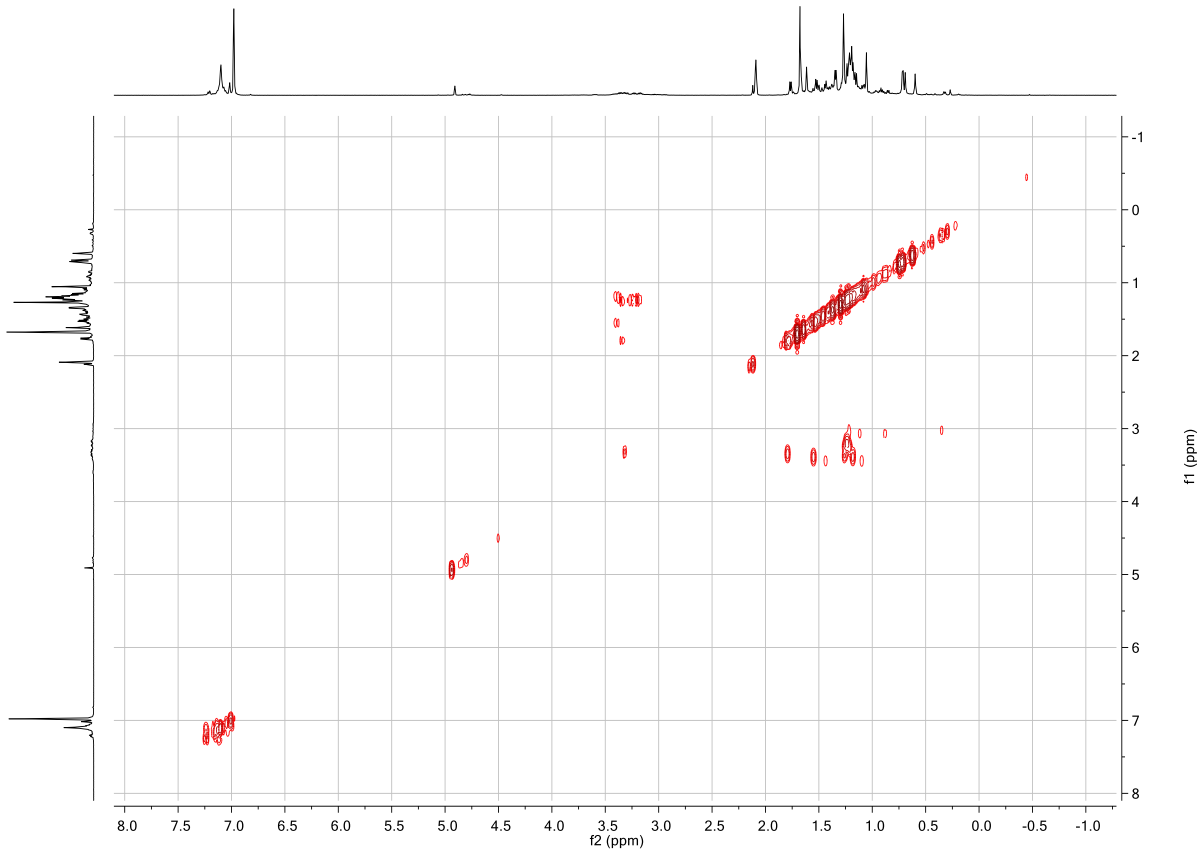

Figure S5: ^{1}H-^{13}C HSQC trace of **3**.

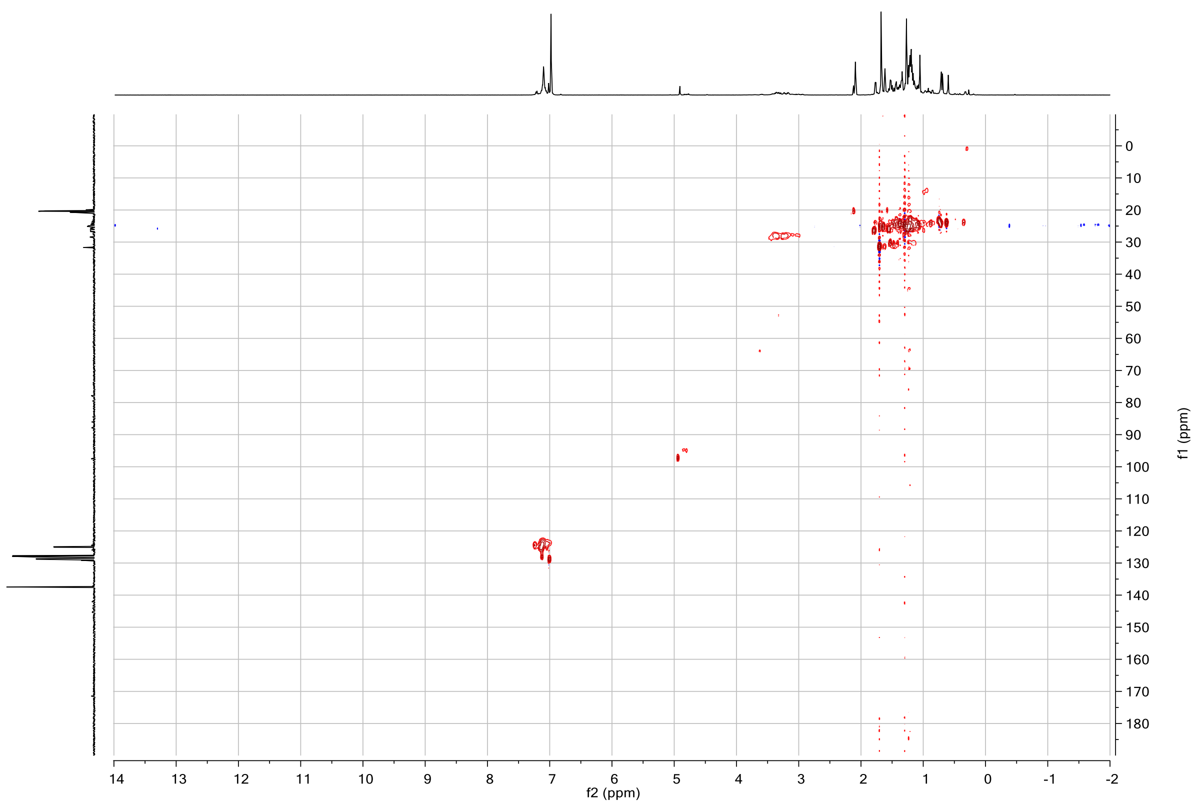

Figure S6: $^{13}C\{^{1}H\}$ DEPT analysis of **3**.

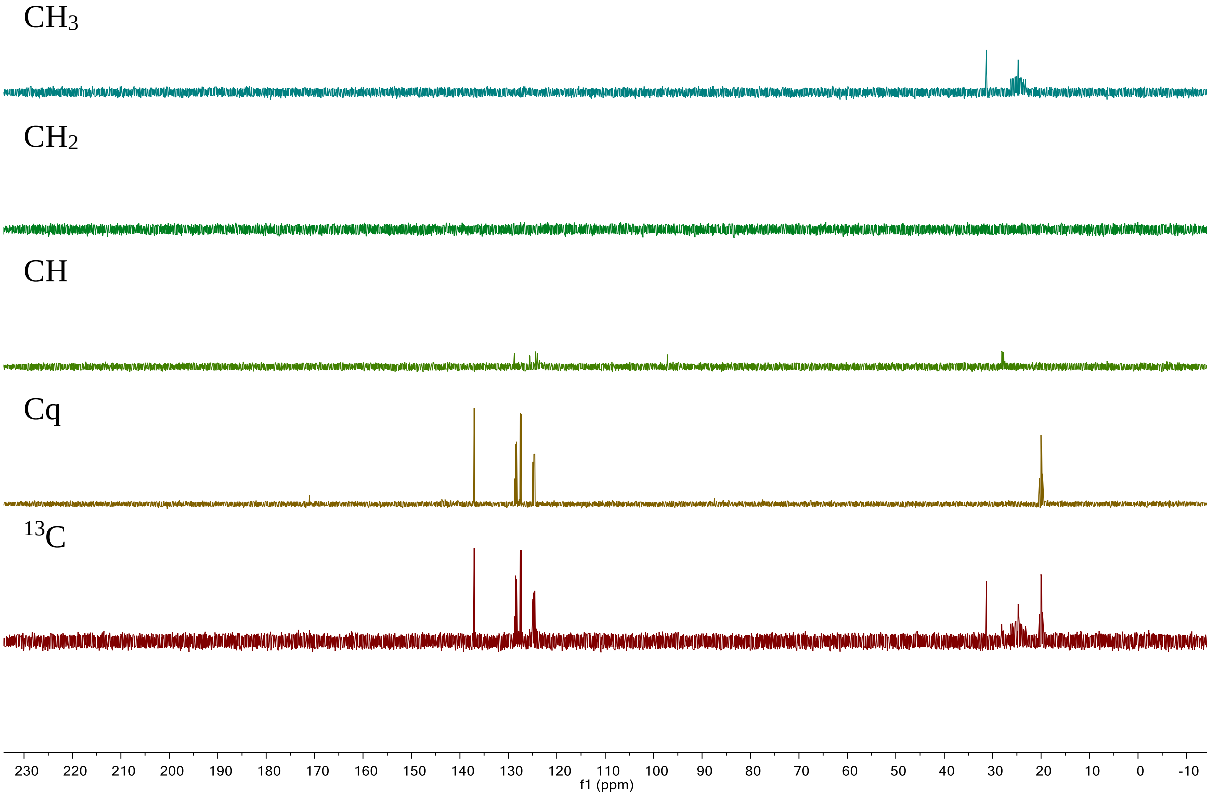

Synthesis of [(BDI^{Dipp})Ca{(pinB)$_2$-C(N(*t*-Bu)(Bpin))}] (**4**)

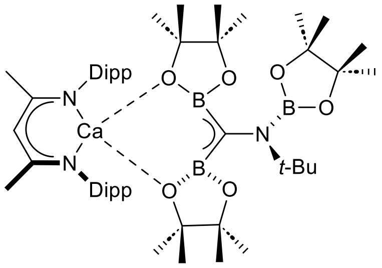

In a J Youngs NMR tube, *t*-BuNC (3.02 mg, 0.04 mmol) was added to a d_8-toluene (*ca*. 0.5 mL) solution of [(BDI^{Dipp})Ca{pinB(Bpin*n*Bu)}] (**2**, 86.9 mg, 0.11 mmol). The resultant brown solution was left at room temperature overnight. The solvent was removed *in-vacuo* and the crude brown solid was redissolved in a mixture of toluene and *n*-hexane. Colourless block crystals (15.2 mg, 45%) deposited at room temperature to provide compound **4**. Despite repeated attempts, a meaningful elemental microanalysis could not be obtained for this compound. ^{1}H NMR (500 MHz, 298 K, d_8-Tol): δ 7.10 (m, 6H, Ar-*H*), 4.70 (s, 1H, NC(CH$_3$)C*H*), 3.28 (m, 1H, C*H*(CH$_3$)$_2$), 3.23 (m, 1H, C*H*(CH$_3$)$_2$), 2.83 (m, 1H, C*H*(CH$_3$)$_2$), 1.68 (s, 6H, NC(C*H$_3$*)CH), 1.66 (s, 12H, C*H*$_3$), 1.45 (d, 3H, $^3J_{HH}$ = 7.0 Hz, C*H$_3$*), 1.36 (d, 3H, $^3J_{HH}$ = 6.7 Hz, C*H$_3$*), 1.28-1.24 (m, 24H, C*H$_3$*), 1.21 (d, 12H, $^3J_{HH}$ = 5.8 Hz, C*H$_3$*), 1.18 (d, 3H, $^3J_{HH}$ = 6.8 Hz, C*H$_3$*), 1.14 (d, 3H, $^3J_{HH}$ = 6.7 Hz, C*H$_3$*), 0.79 (s, 9H, C*H*$_3$) ppm. ^{13}C{^{1}H} NMR (126 MHz, 298 K, d_8-Tol): δ 167.0, 166.7 (N*C*(CH$_3$)CH), 146.4, 146.3, 141.3, 141.2, 141.1, 124.4, 124.4, 124.4, 124.3 (*C* Ar), 123.8 (B*C*N), 89.0 (NC(CH$_3$)*C*H), 54.1 (B(O*C*(CH$_3$)$_2$)$_2$), 32.1 (B(OC(*C*H$_3$)$_2$)$_2$), 30.2, 29.7, 28.9, 28.7 (*C*H(CH$_3$)$_2$), 28.1, 27.2, 25.8, 25.6, 25.4, 25.3, 25.3, 25.2 (*C*H$_3$), 25.2 (B(OC(*C*H$_3$)$_2$)$_2$), 25.1 (*C*H$_3$), 24.9 (NC(*C*H$_3$)CH), 24.4, 24.4, 24.3 (*C*H$_3$), 23.7 (NC(*C*H$_3$)$_3$) ppm. *^{13}C resonance correlated to B$_2$CN was not observed. ^{11}B{^{1}H} NMR (160 MHz, 298 K, d_8-Tol): δ 34.3, 25.5 ppm.

Figure S7: ^{1}H NMR spectrum (500 MHz, 298 K, d_8-Tol) of **4**.

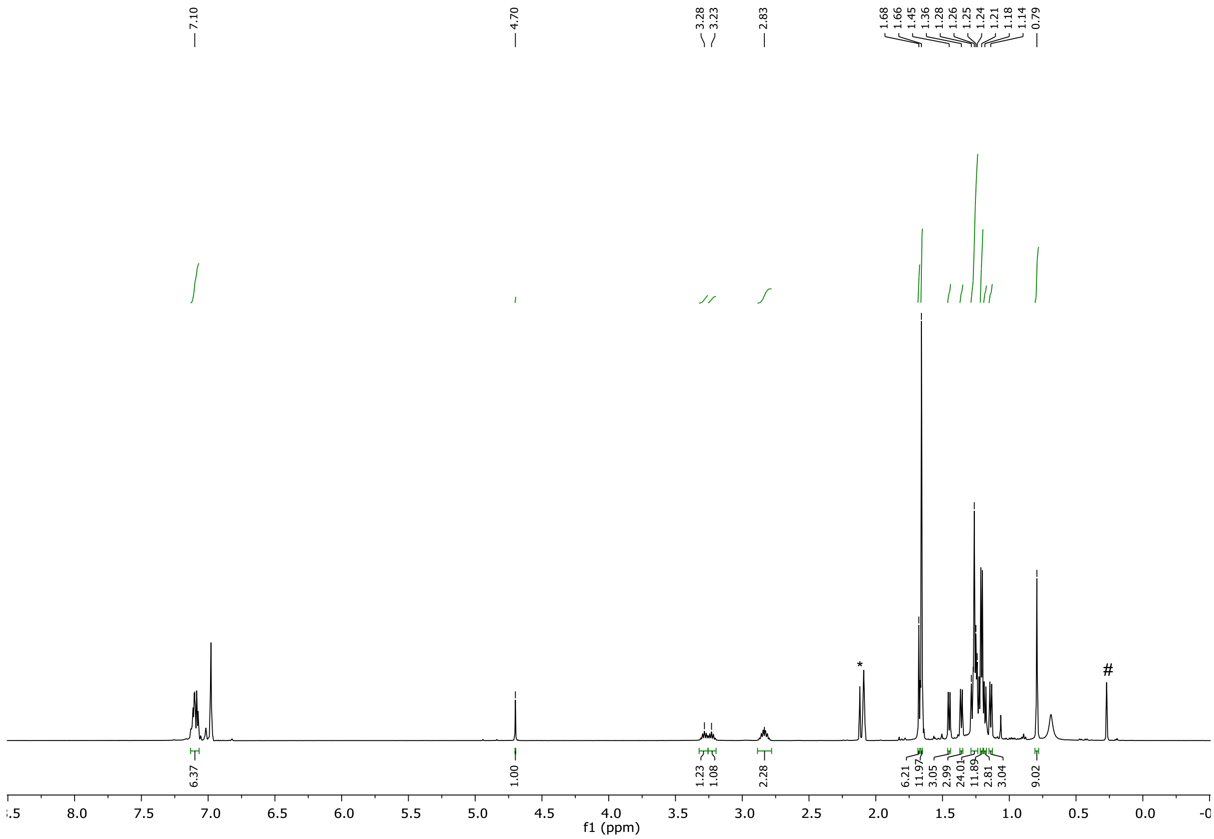

Figure S8: ^{13}C NMR spectrum (126 MHz, 298 K, d_8-Tol) of **4**.

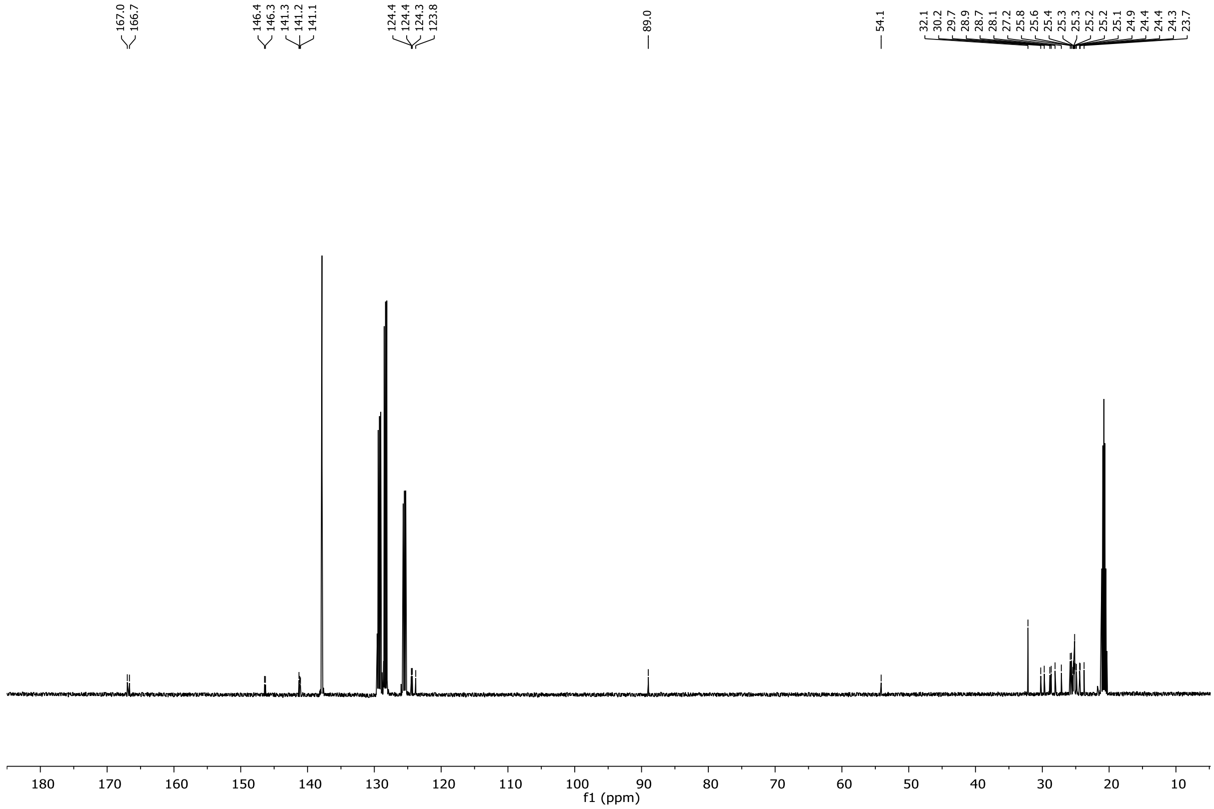

Figure S9: $^{11}B\{^1H\}$ NMR spectrum (160 MHz, 298 K, d_8-Tol) of **4**.

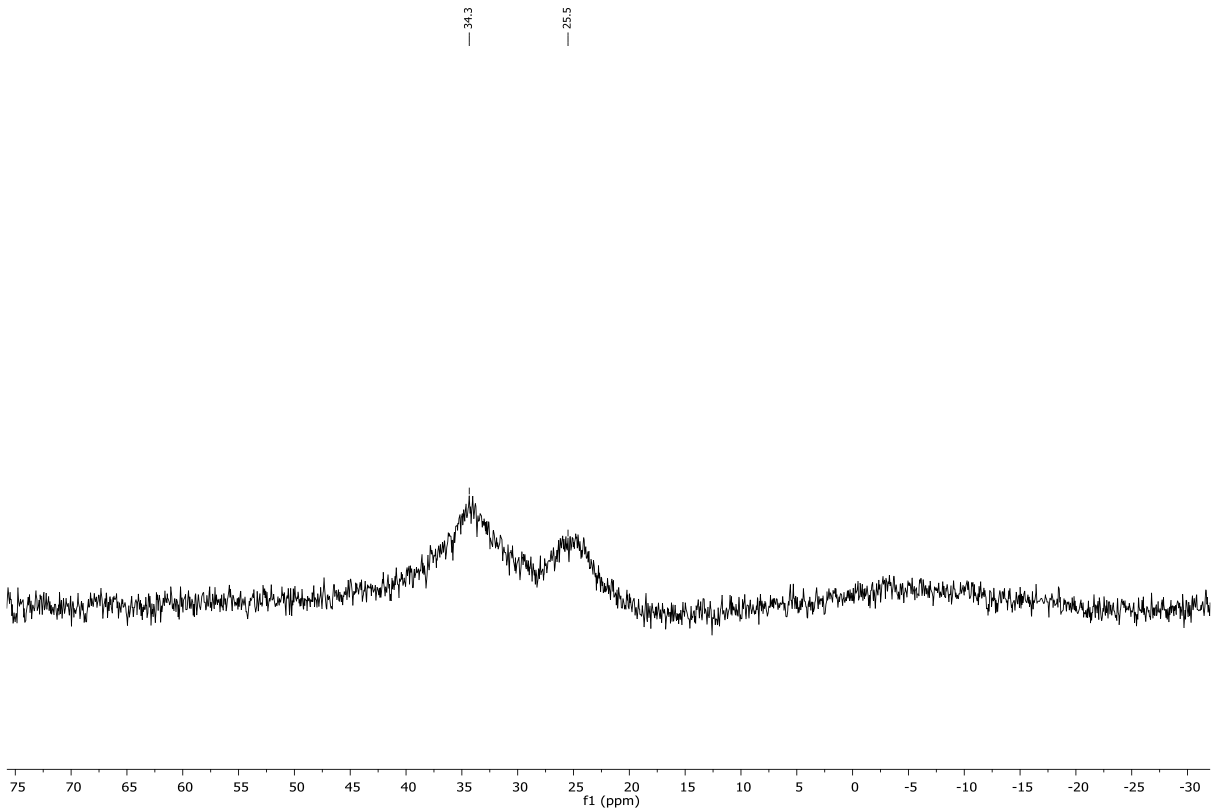

Figure S10: ^1H-^1H COSY trace of **4**.

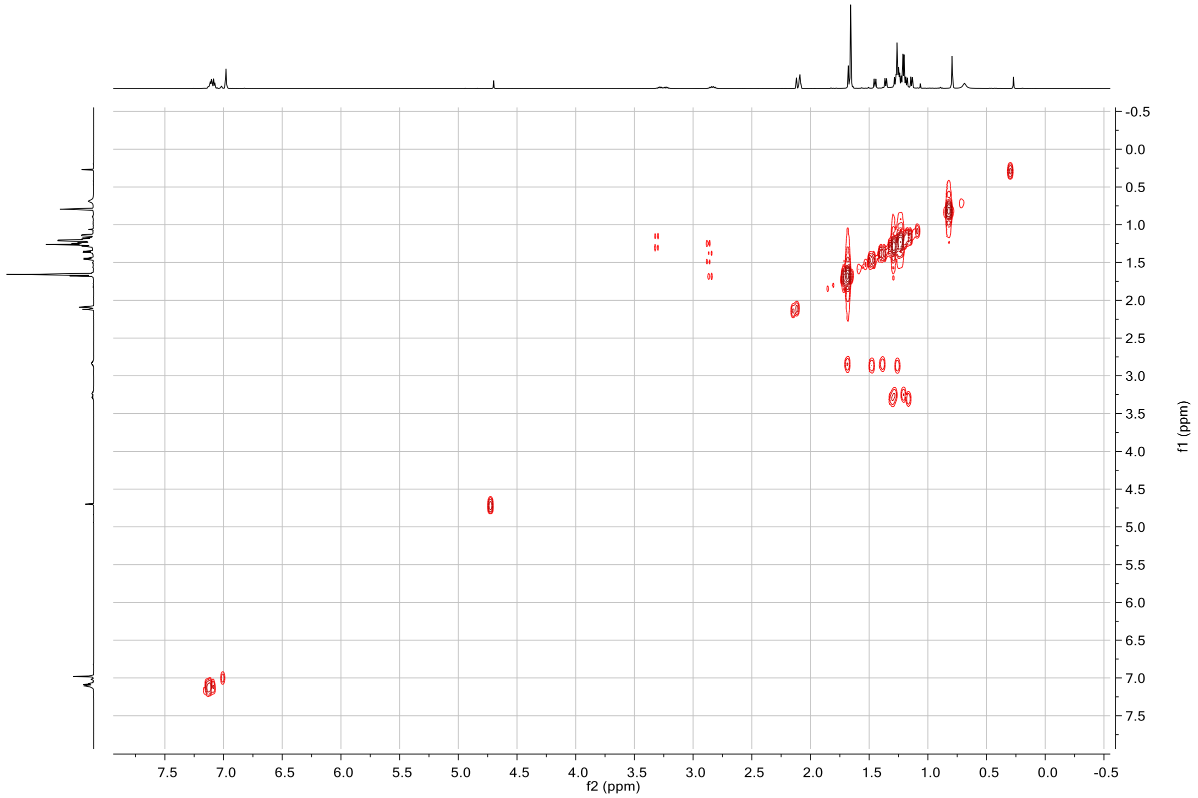

Figure S11: ^{1}H-^{13}C HSQC trace of **4**.

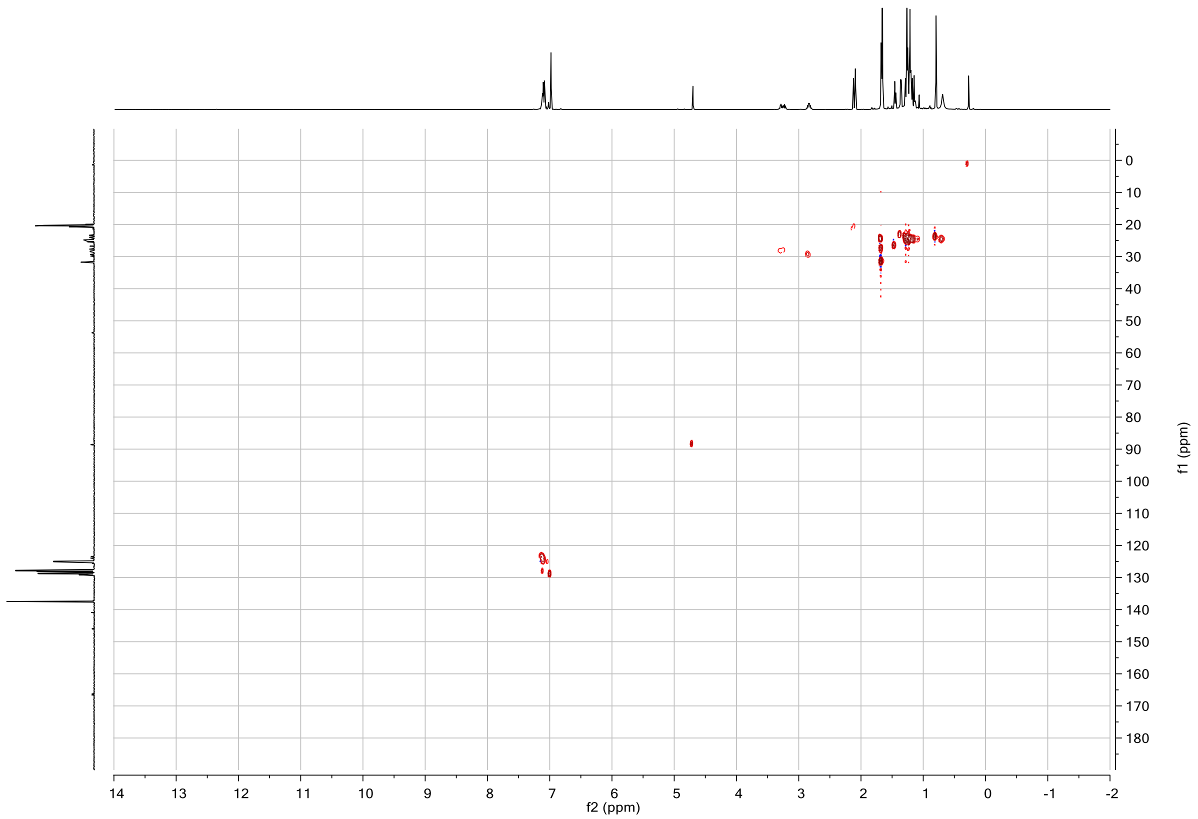

Figure S12: ^{1}H-^{13}C HMBC trace of **4**.

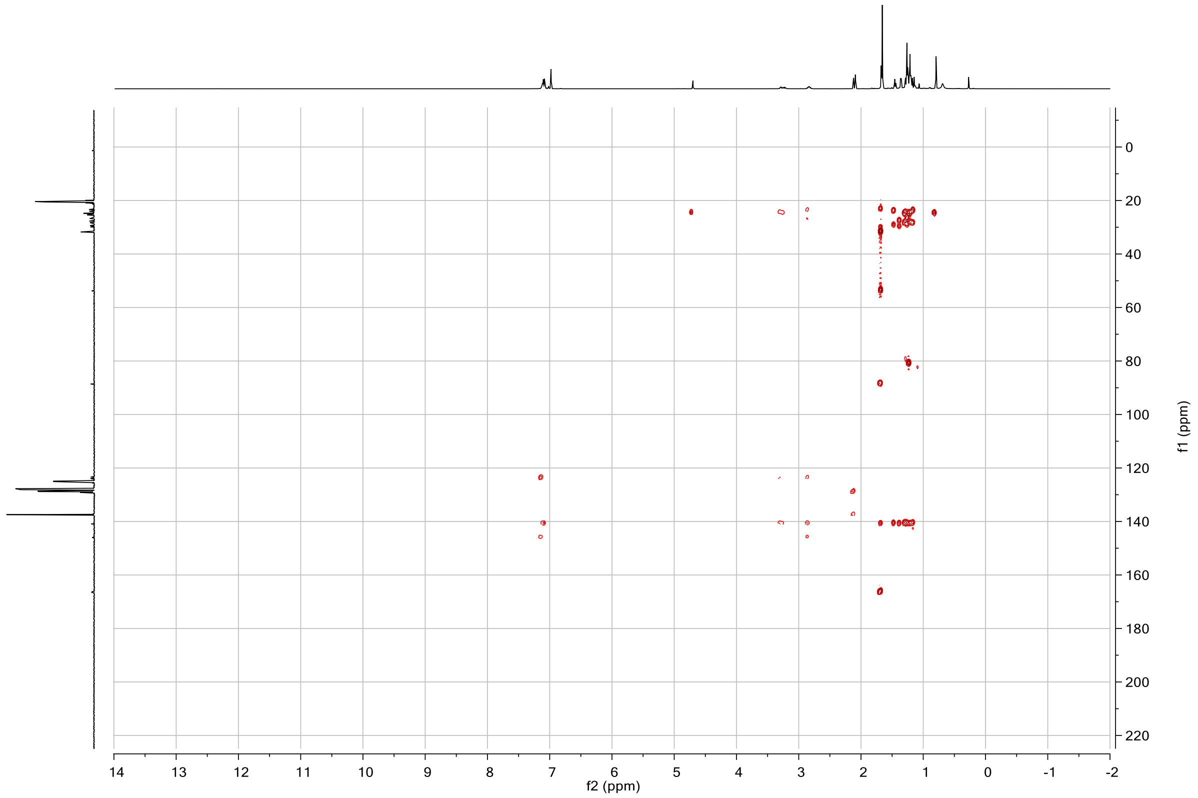

Synthesis of [(BDI^{Dipp})Mg{(pinB)2-C(NCy(Bpin)}] **(7)**

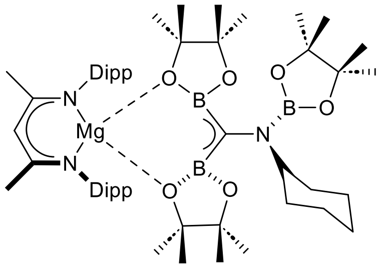

In a J Youngs NMR tube, *Cy*NC (10.9 mg, 12.4 μL, 0.10 mmol) was added to a colourless d_6-benzene (*ca*. 0.5 mL) solution of [(BDI^{Dipp})Mg{pinB}$_3$] (**5** 82.3 mg, 0.10 mmol). The reaction mixture was observed to change into a pale-yellow solution. The reaction mixture was then left at room temperature overnight, and the resulting solution was now orange. Slow evaporation of the benzene solution in the glovebox afforded compound **7** as colourless crystals suitable for X-ray single-crystal diffraction. Yield 61.8 mg, 66%. Anal. Calcd. For $C_{54}H_{88}B_3MgN_3O_6$ (**7**): C, 69.59; H, 9.52; N, 4.51%. Found: C, 69.50; H, 9.37; N, 3.92%. ^{1}H NMR (500 MHz, 298 K, Benzene-d_6) δ 7.27 (d, $^3J_{HH}$ = 7.6 Hz, *p*-C_6H_3), 7.18 (d, J = 7.5 Hz, 1H, *p*-C_6H_3), 7.14 – 7.08 (m, 4H, *m*-C_6H_3), 4.88 (s, 1H, NC(CH$_3$)C*H*), 3.48 – 3.32 (m, 3H, C*H*(CH$_3$)$_2$ and C*H*(CH$_2$)$_5$), 3.20 (2 x sept, $^3J_{HH}$ = 6.8 Hz, 2H, C*H*(CH$_3$)$_2$), 2.40 – 2.30 (m, 2H, CH(C*H*$_2$)$_5$), 2.12 – 1.93 (m, 4H, CH(C*H*$_2$)$_5$), 1.80 (d, $^3J_{HH}$ = 6.8 Hz, 3H, CH(C*H*$_3$)$_2$), 1.67 (s, 3H, NC(C*H*$_3$)CH), 1.66 (d, $^3J_{HH}$ = 6.8 Hz, 3H, CH(C*H*$_3$)$_2$), 1.64 (s, 3H, NC(C*H*$_3$)CH), 1.57 – 1.48 (m, 2H, CH(C*H*$_2$)$_5$), 1.34 (d, $^3J_{HH}$ = 6.8 Hz, 9H, CH(C*H*$_3$)$_2$), 1.28 (s, 6H, C*H*$_3$), 1.26 (s, 3H, C*H*$_3$), 1.25 – 1.22 (m, 15H, C*H*$_3$), 1.20 (s, 3H, C*H*$_3$), 1.18 (d, $^3J_{HH}$ = 6.8 Hz, 3H, CH(C*H*$_3$)$_2$), 1.17 (d, $^3J_{HH}$ = 6.8 Hz, 3H, CH(C*H*$_3$)$_2$), 1.16 – 1.07 (m, 2H, CH(C*H*$_2$)$_5$), 1.02 (s, 6H, C*H*$_3$), 0.76 (s, 3H, C*H*$_3$), 0.74 (s, 3H, C*H*$_3$), 0.67 (s, 3H, C*H*$_3$), 0.60 (s, 3H, C*H*$_3$) ppm. ^{13}C NMR (126 MHz, 298 K, Benzene-d_6) δ 171.4 (N*C*(CH$_3$)CH), 171.3((N*C*(CH$_3$)CH), 145.2, 145.1, 144.0, 143.4, 142.2, 142.1 (*o*-*C*$_6$H$_3$ and *i*-*C*$_6$H$_3$), 125.9, 124.9 124.7 124.5 (*m*-*C*$_6$H$_3$ and *p*-*C*$_6$H$_3$) 97.3 (NC(CH$_3$)*C*H), 88.0, 86.3 (B(O*C*(CH$_3$)$_2$)$_2$), 83.0 (B$_2$*C*N), 78.0 (B(O*C*(CH$_3$)$_2$)$_2$), 62.5 (*C*H(CH$_2$)$_5$), 33.2, 33.0 (CH(*C*H$_2$)$_5$), 28.5, 28.5, 28.4, 28.2, 27.9, 27.8 (*C*H(CH$_3$)$_2$ and CH(*C*H$_2$)$_5$), 27.1, 26.9, 26.4, 26.3, 26.3, 26.2, 25.8, 25.6, 25.5, 25.5, 25.4, 25.4, 25.3 (*C*H$_3$), 25.2, 25.2 (NC(*C*H$_3$)CH), 25.0, 24.5, 24.5, 24.4, 24.3, 24.1, 23.7 (*C*H$_3$) ppm. ^{11}B{^{1}H} NMR (160 MHz, 298 K, Benzene-d_6) δ 34.4, 31.3, 23.9 ppm.

Figure S13: ^{1}H NMR spectrum (500 MHz, 298 K, Benzene-d_6) of **7**.

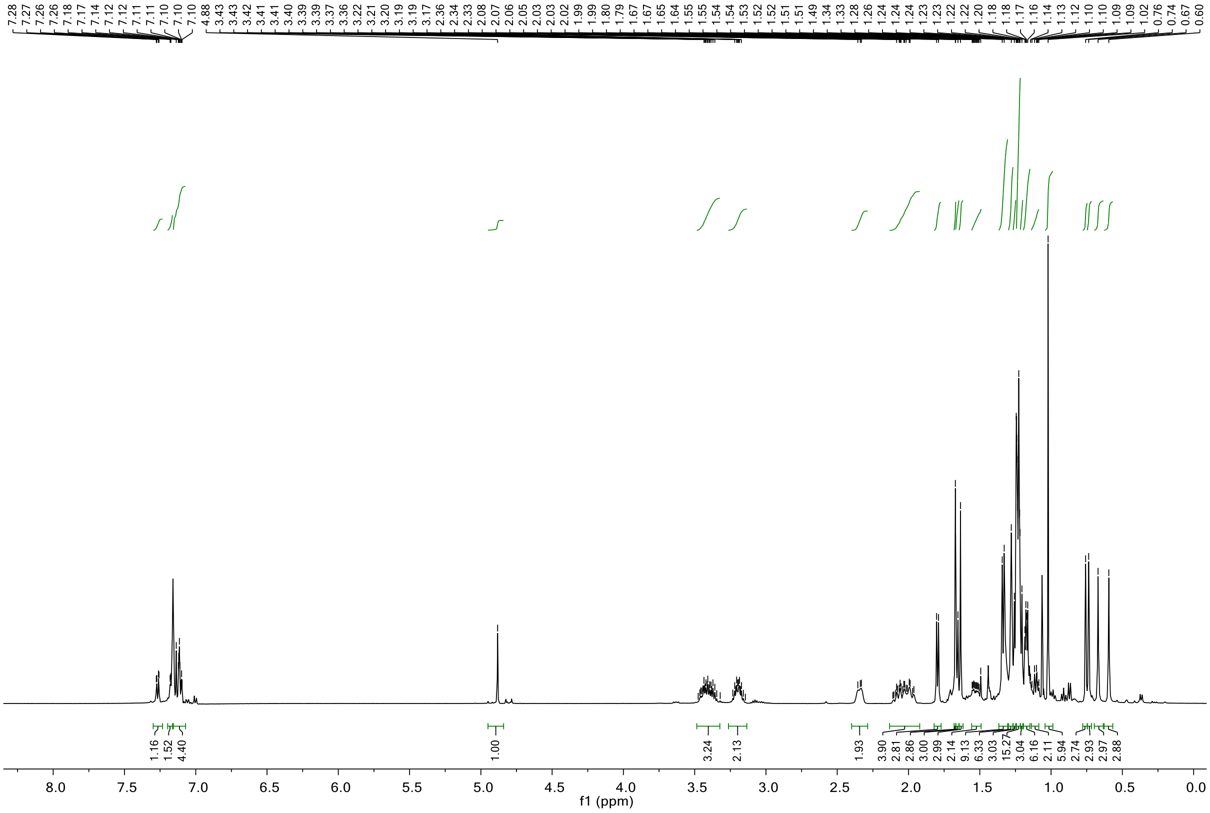

Figure S14: ^{13}C{^{1}H} NMR spectrum (126 MHz, 298 K, Benzene-d_6) of **7**.

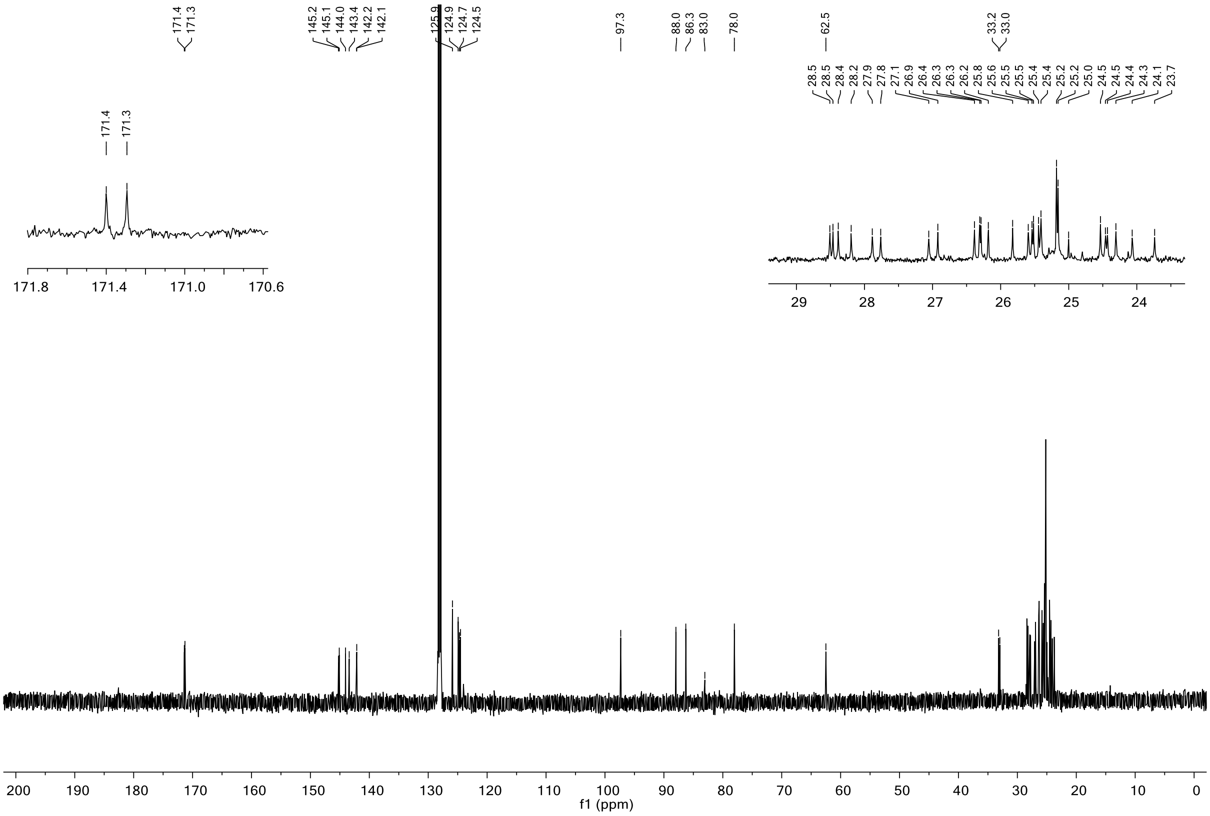

Figure S15: $^{11}B\{^{1}H\}$ NMR spectrum (160 MHz, 298 K, Benzene-d_6) of **7**.

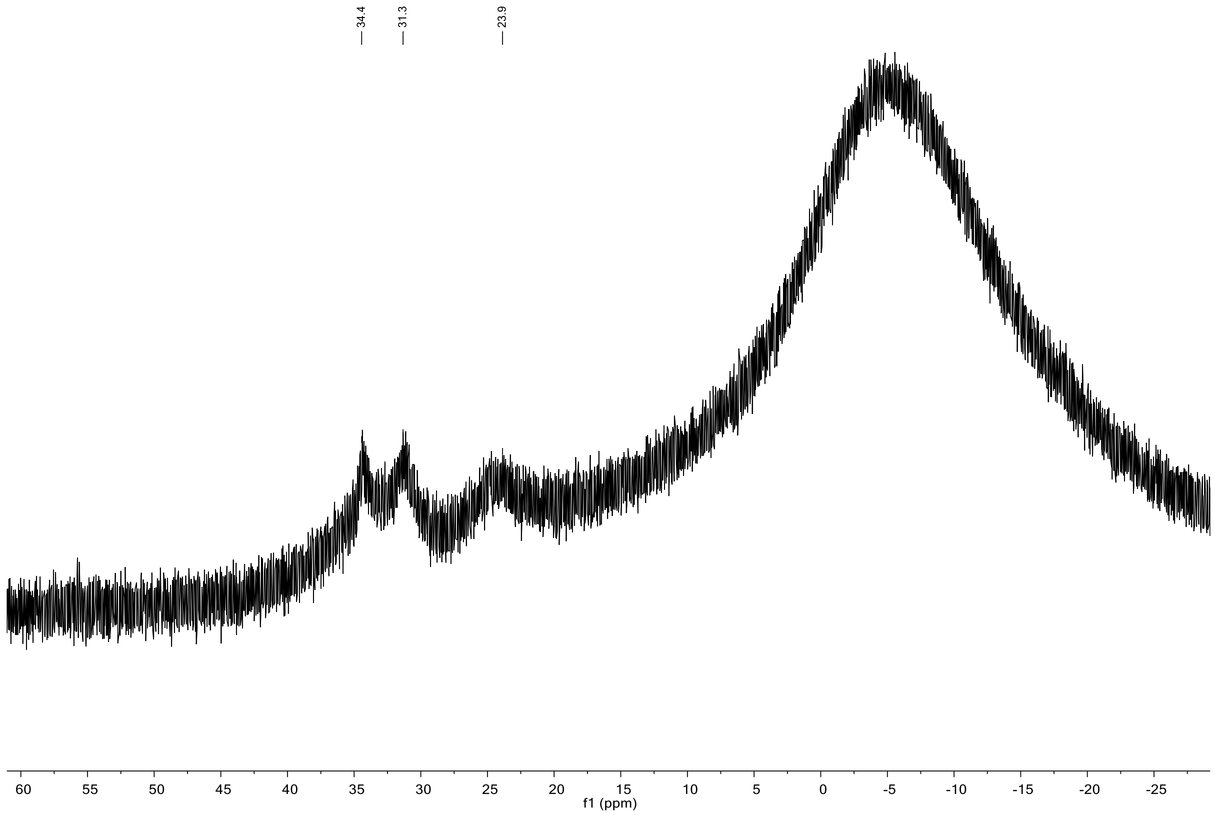

Figure S16: ^{1}H-^{1}H COSY trace of **7**.

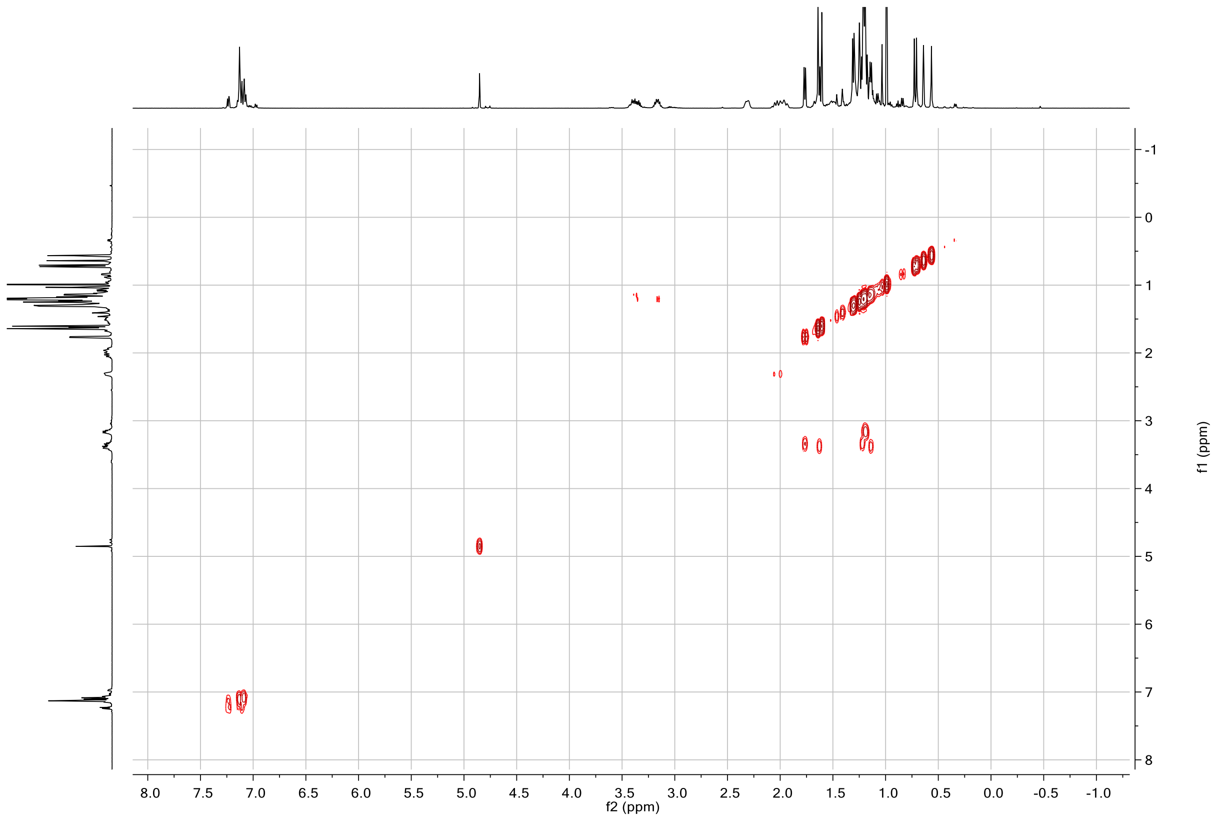

Figure S17: ^{1}H-^{13}C HSQC trace of **7**.

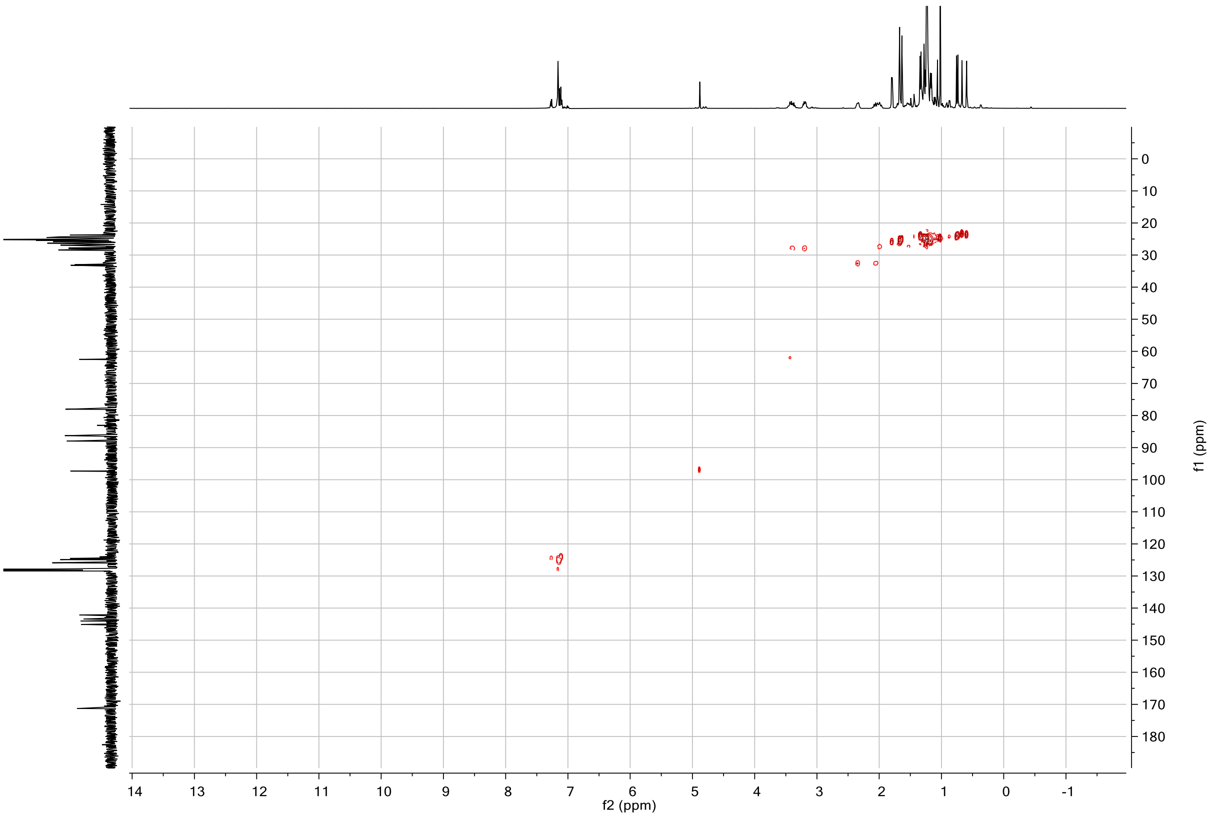

Figure S18: ^{1}H-^{13}C HMBC trace of **7**.

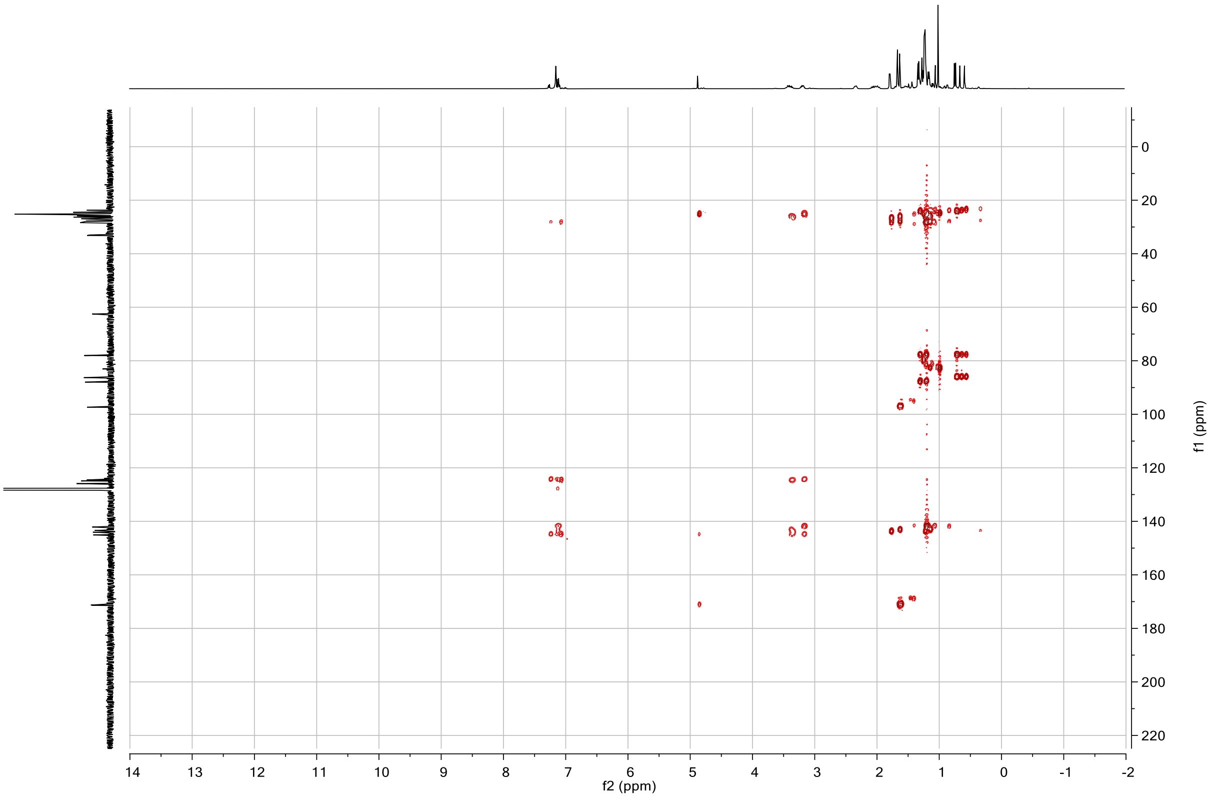

Synthesis of [(BDI^{Dipp})Mg{(neoB)(pinB)-C(N(*t*-Bu)(Bneo)}] (**8**)

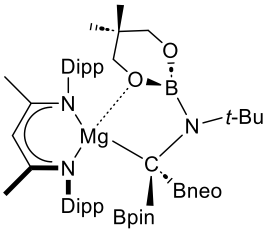

In a J Youngs NMR tube, *t*-BuNC (8.3 mg, 11.3 μL, 0.10 mmol) was added to a colourless d_6-benzene (*ca*. 0.5 mL) solution of [(BDI^{Dipp})Mg{(neo)BB(neo)Bpin}] (**6**, 79.5 mg, 0.10 mmol). The reaction mixture was observed to change into an orange solution upon addition of the isonitrile. The reaction mixture was then left at room temperature overnight, and the resulting solution was now pale-red in colour. Slow evaporation of the benzene solution in the glovebox afforded compound **8** as colourless crystals suitable for X-ray single-crystal diffraction. Yield 53.8 mg, 61%. Anal. Calcd. For $C_{50}H_{83}B_3MgN_3O_6$ (**8**): C, 68.40; H, 9.41; N, 4.79%. Found: C, 68.80; H, 9.34; N, 4.31%. ^{1}H NMR (500 MHz, 298 K, Benzene-d_6) δ 7.15 – 7.00 (m, 6H, Ar*H*), 4.82 (s, 1H, NC(CH$_3$)C*H*), 4.01 – 3.88 (m, 2H, BOCH_2C(CH$_3$)$_2$), 3.54 (s, 2H, BOCH_2C(CH$_3$)$_2$), 3.48 – 3.29 (m, 2H, C*H*(CH$_3$)$_2$), 3.22 (2 x sept, $^3J_{HH}$ = 6.8 Hz, 2H, C*H*(CH$_3$)$_2$), 2.95 (d, $^4J_{HH}$ = 9.9 Hz, 2H, BOCH_2C(CH$_3$)$_2$), 2.71 (d, $^4J_{HH}$ = 9.9 Hz, 2H, BOCH_2C(CH$_3$)$_2$), 1.82 (s, 9H, C(CH_3)$_3$), 1.64 (d, $^3J_{HH}$ = 6.8 Hz, 3H, CH(CH_3)$_2$), 1.59 (s, 3H, NC(CH_3)CH), 1.55 (d, $^3J_{HH}$ = 6.8 Hz, 3H, CH(CH_3)$_2$), 1.52 (d, $^3J_{HH}$ = 6.8 Hz, 3H, CH(CH_3)$_2$), 1.48 (s, 3H, NC(CH_3)CH), 1.46 (d, $^3J_{HH}$ = 6.8 Hz, 3H, CH(CH_3)$_2$), 1.30 (d, $^3J_{HH}$ = 6.8 Hz, 3H, CH(CH_3)$_2$), 1.25* (d, $^3J_{HH}$ = 6.8 Hz, 6H, CH(CH_3)$_2$) 1.23* (d, $^3J_{HH}$ = 6.8 Hz, 3H, CH(CH_3)$_2$) *overlapping signals, 0.98 (s, 6H, BOC(CH_3)$_2$), 0.87 (s, 6H, BOCH$_2$C(CH_3)$_2$), 0.79 (s, 6H, BOC(CH_3)$_2$), 0.67 (s, 6H, BOCH$_2$C(CH_3)$_2$) ppm. ^{13}C NMR (126 MHz, 298 K, Benzene-d_6) δ 169.2 (N*C*(CH$_3$)CH), 169.2 (N*C*(CH$_3$)CH), 148.3 (*i*-C_6H$_3$), 146.9 (*i*-C_6H$_3$), 143.2 (*o*-C_6H$_3$), 143.1 (*o*-C_6H$_3$), 142.8 (*o*-C_6H$_3$), 142.7 (*o*-C_6H$_3$), 125.5, 125.0, 124.8, 124.5, 124.4, 124.1 (*m*- and *p*-C_6H$_3$), 95.9 (NC(CH$_3$)*C*H), 80.3 (BO*C*H$_2$C(CH$_3$)$_2$), 76.2 (BO*C*(CH$_3$)$_2$), 71.8 (BO*C*H$_2$C(CH$_3$)$_2$), 71.1 (BO*C*(CH$_3$)$_2$), 55.7 (*C*(CH$_3$)$_3$), 31.9 (BOCH$_2$*C*(CH$_3$)$_2$), 31.3 (BOCH$_2$*C*(CH$_3$)$_2$), 30.8 (C(*C*H$_3$)$_3$), 28.7, 28.6, 28.1, 28.0 (*C*H(CH$_3$)$_2$), 27.3 (BOC(*C*H$_3$)$_2$), 26.4, 26.2, 25.3, 25.2 (CH(*C*H$_3$)$_2$), 25.1 (NC(*C*H$_3$)CH), 25.0, 24.9, 24.8, 24.7 (CH(*C*H$_3$)$_2$), 24.6 (NC(*C*H$_3$)CH), 23.9 (BOC(*C*H$_3$)$_2$), 22.6 (BOCH$_2$C(*C*H$_3$)$_2$), 22.5 (BOCH$_2$C(*C*H$_3$)$_2$) ppm. *^{13}C resonance correlated to B$_2$*C*N was not observed. ^{11}B{^{1}H} NMR (160 MHz, 298 K, Benzene-d_6) δ 34.4, 31.8, 20.6 ppm.

Figure S19: ^{1}H NMR spectrum (500 MHz, 298 K, Benzene-d_6) of **8**.

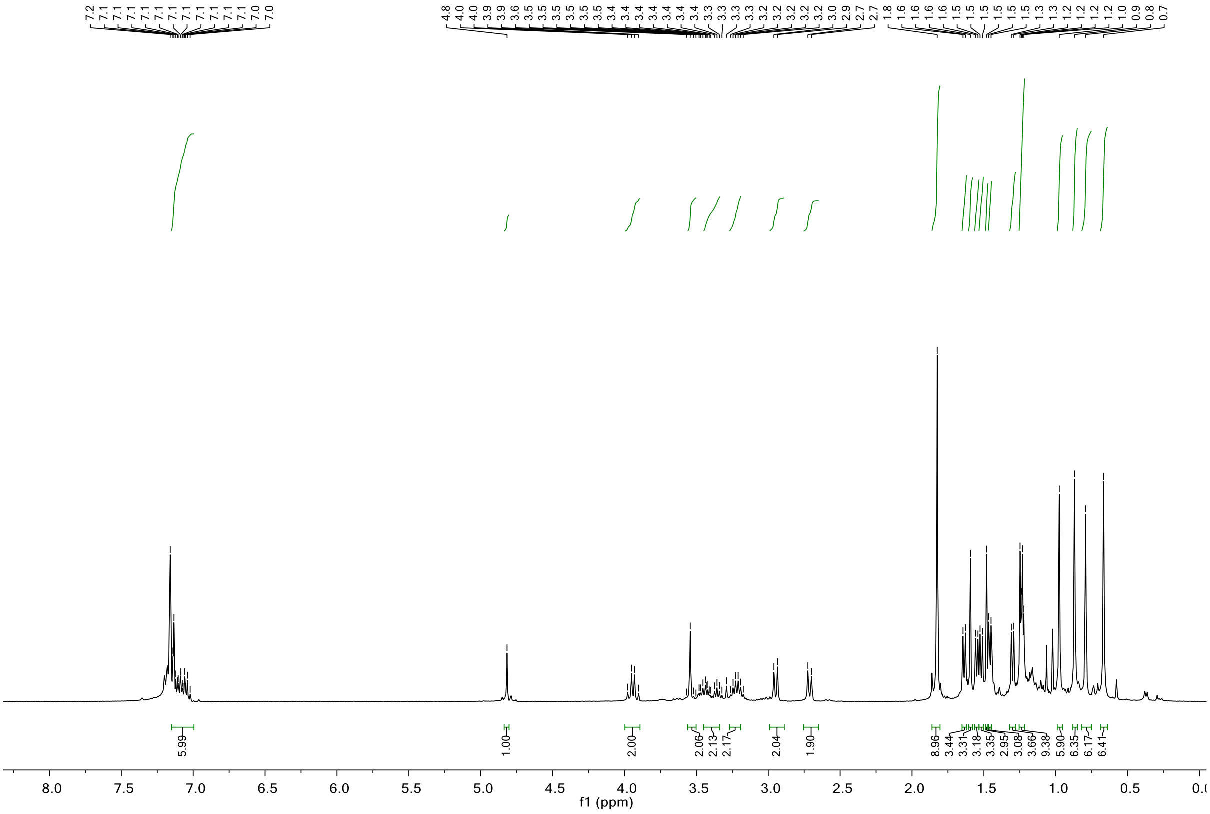

Figure S20: ^{13}C{^{1}H} NMR spectrum (126 MHz, 298 K, Benzene-d_6) of **8**.

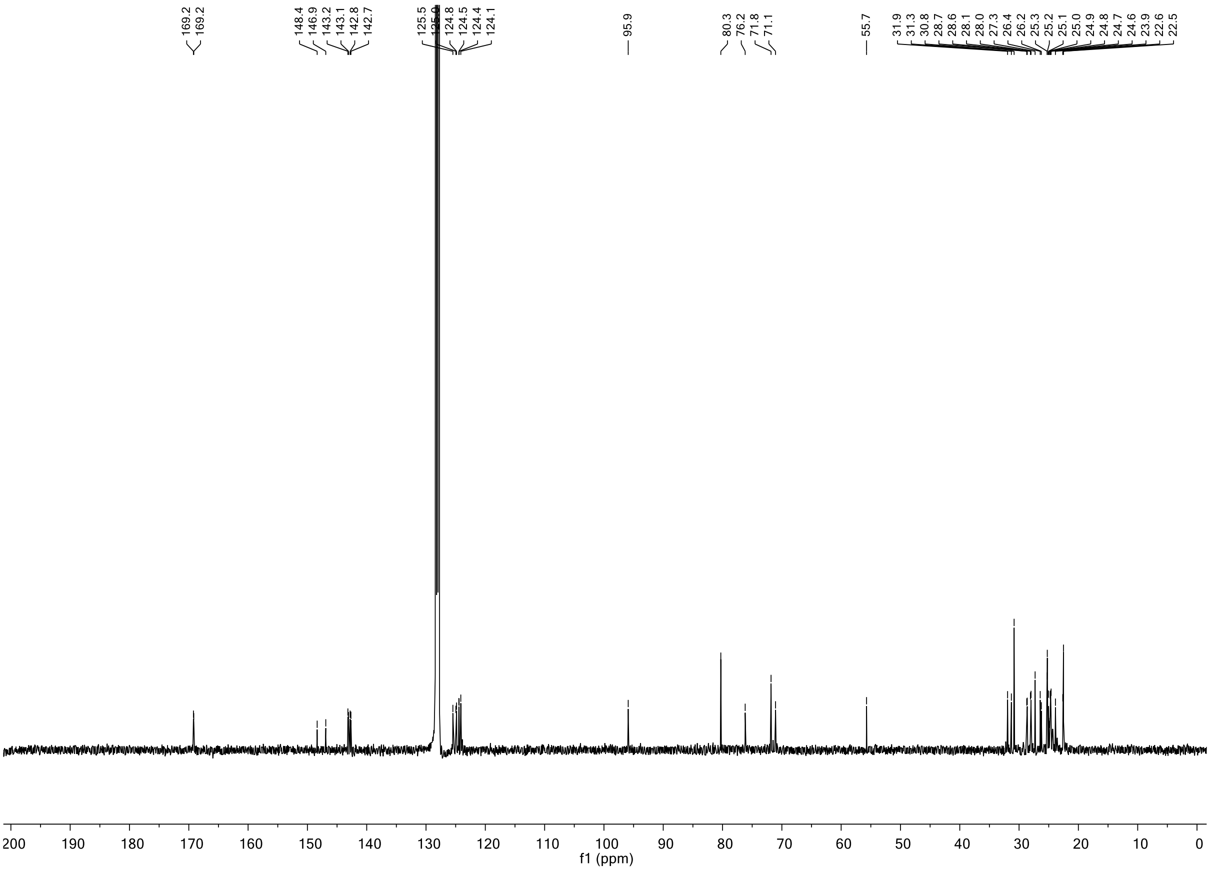

Figure S21: $^{11}B\{^{1}H\}$ NMR spectrum (160 MHz, 298 K, Benzene-d_6) of **8**.

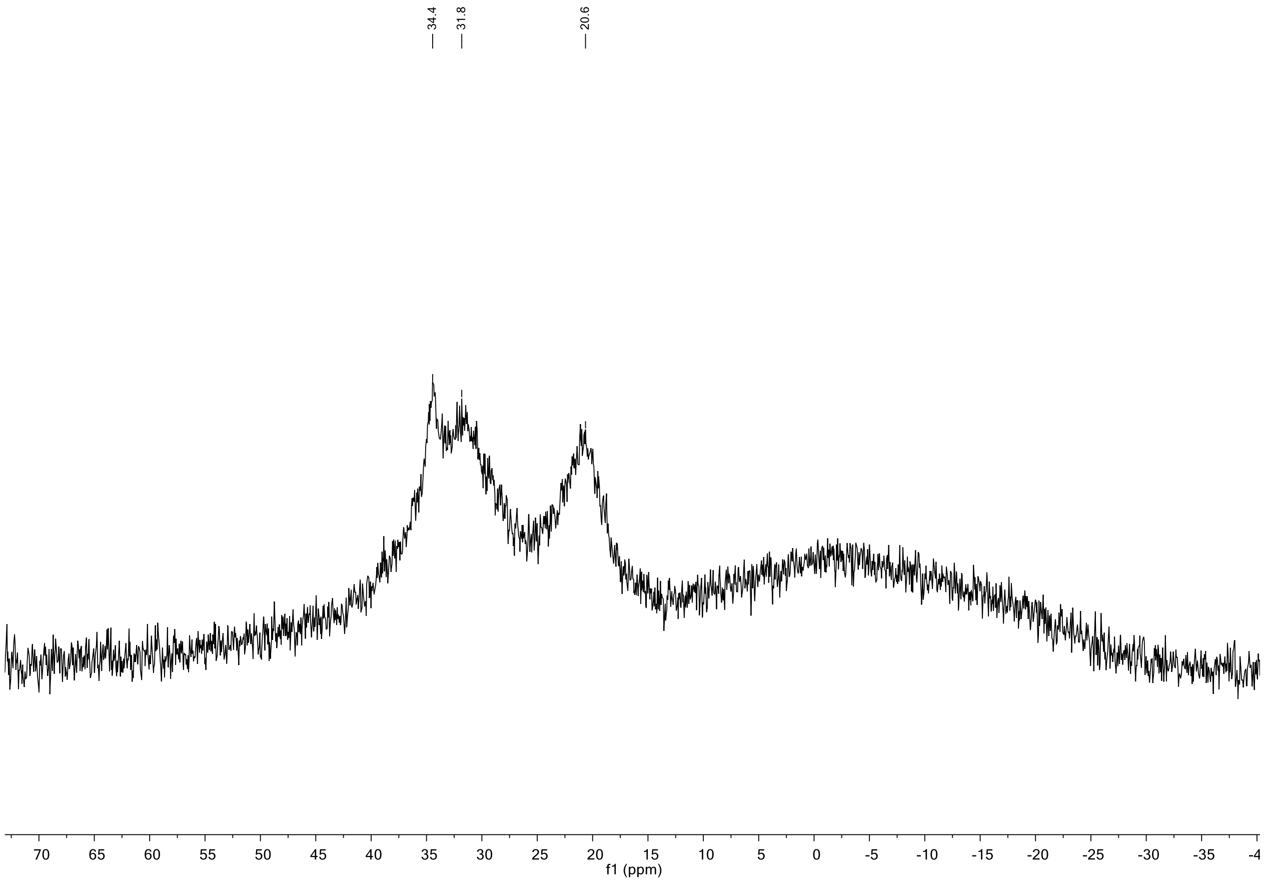

Figure S22: ^{1}H-^{1}H COSY trace of **8**.

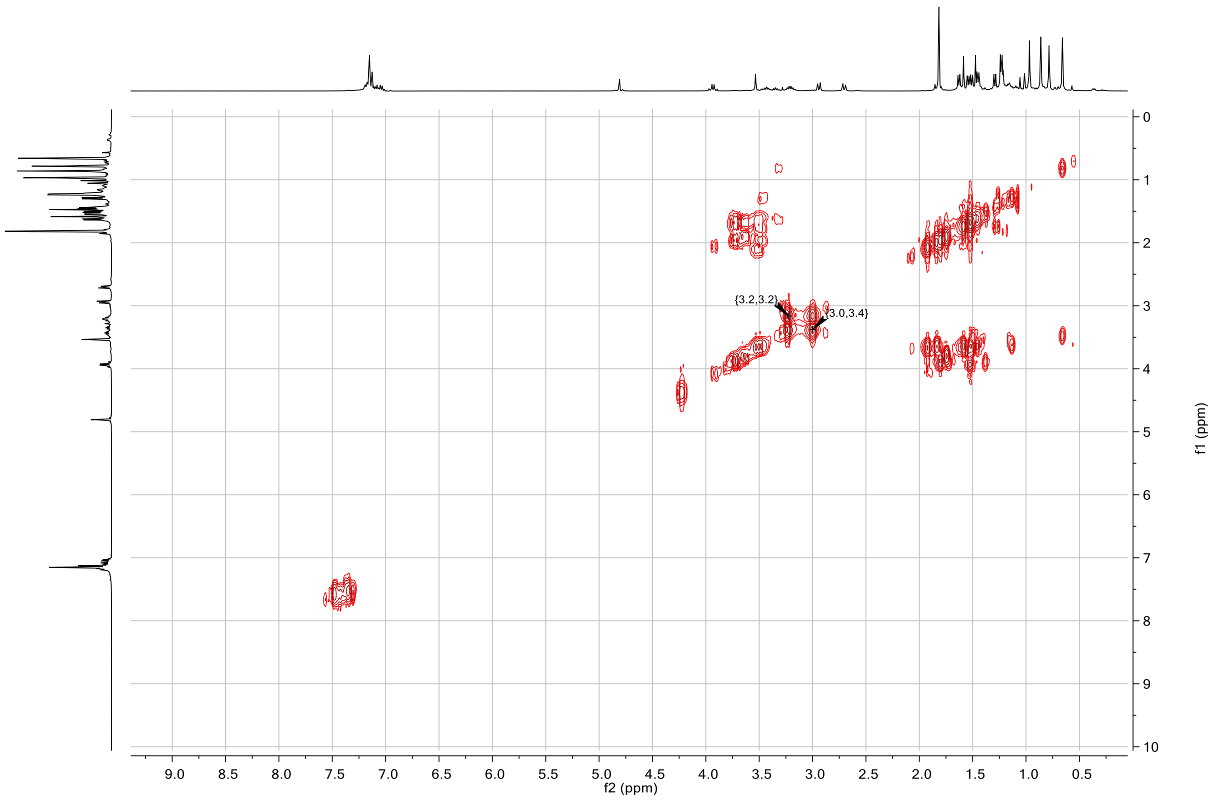

Figure S23: ^{1}H-^{13}C HSQC trace of **8**.

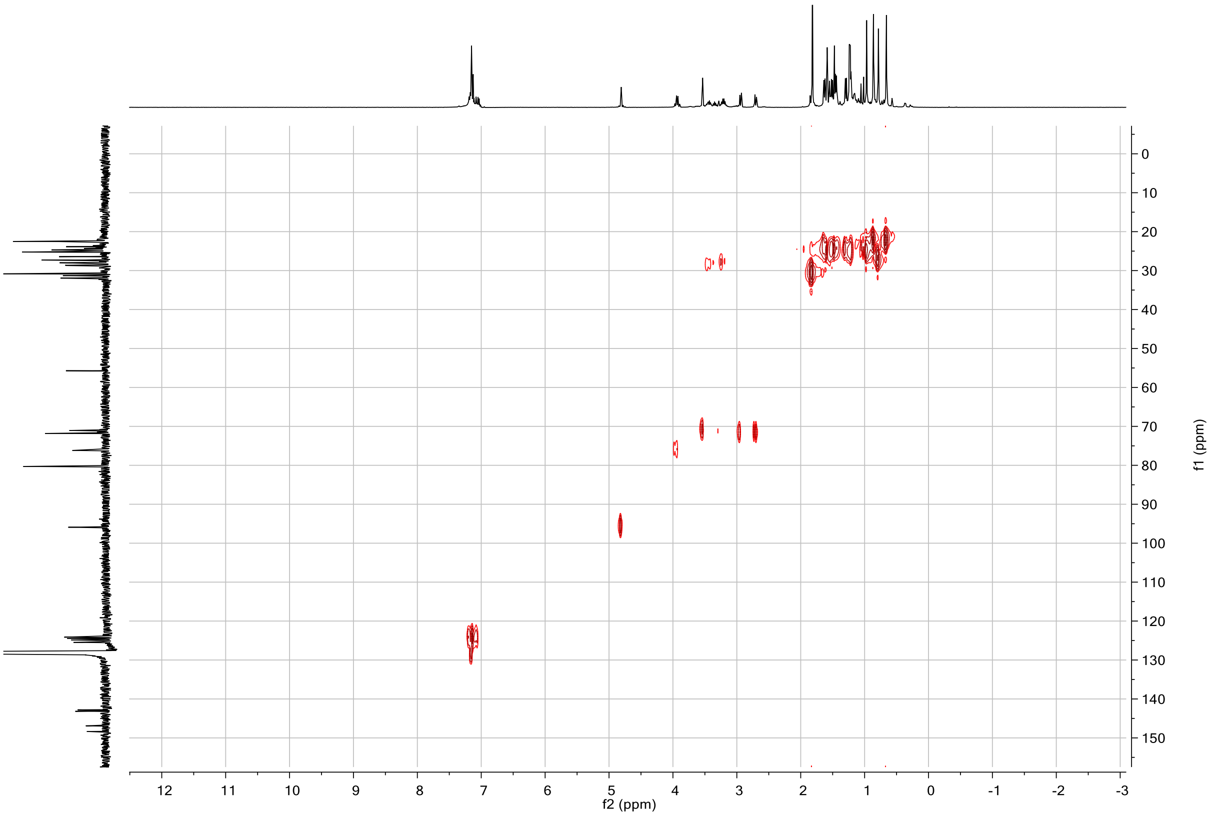

Figure S24: ^{1}H-^{13}C HMBC trace of **8**.

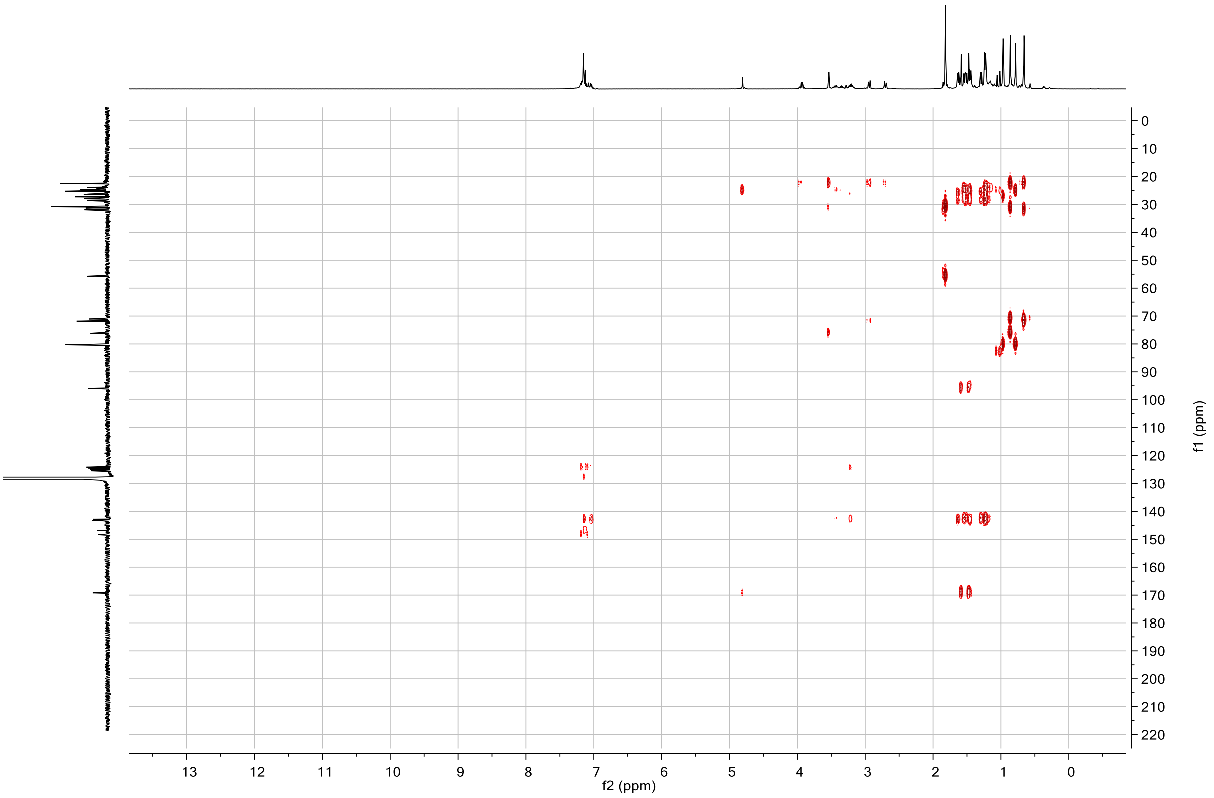

Crystallographic Details

Single Crystal X–ray diffraction data were collected on a SuperNova, EosS2 diffractometer using CuKα (λ = 1.54184 Å) radiation, except for **8** which were collected on an Xcalibur, EosS2 diffractometer using MoKα (λ = 0.71073 Å) radiation. Samples were universally maintained at 150 K during data collection. Using Olex2,[4] the structures were solved with the olex2.solve[5] structure solution program or ShelXT and refined with the ShelXL[6] refinement package using Least−Squares minimisation.

In the structure of **3**, the asymmetric unit comprises one molecule of the magnesium complex and a region of solvent. The isopropyl group in the main feature, based on C24, was modelled to take account of 55:45 disorder while C36-C41 were treated for a 75:25 split. The solvent moiety is a toluene molecule with half site-occupancy, that is disordered with itself, about a crystallographic inversion centre. Distance, planarity and ADP restraints were used in the disordered regions (on merit) to attain a chemically sensible convergence.

The asymmetric unit in **4** was host to one molecule of toluene in addition to one molecule of the calcium complex. The solvent was disordered in a 50:50 ratio over two very proximate sites, and was successfully refined with the addition of distance and ADP restraints.

One molecule of benzene in addition to one molecule of the magnesium complex constitute the asymmetric unit in **7**.

In **8**, the asymmetric unit plays host to one molecule of the magnesium complex and half of a molecule of hexane. The latter lies proximate to a crystallographic inversion centre which serves to generate the remainder. The solvent content was modelled to take account of 85:15 disorder with the inclusion of appropriate distance and ADP restraints.

Table S1: Crystal Data and Structural Refinement for Compounds **3**, **4**, **7** and **8**.

	**3**	**4**	**7**	**8**
Empirical formula	$C_{55.5}H_{90}B_3MgN_3O_6$	$C_{59}H_{94}B_3CaN_3O_6$	$C_{60}H_{94}B_3MgN_3O_6$	$C_{53}H_{89}B_3MgN_3O_6$
Formula weight	952.04	1013.88	1010.12	921.01
Crystal system	monoclinic	triclinic	triclinic	triclinic
Space group	$P2_1/c$	*P*-1	*P*-1	*P*-1
a/ Å	11.5951(1)	11.2516(2)	10.6784(2)	11.6016(4)
b/ Å	19.7465(1)	13.3172(3)	12.5289(2)	11.9817(4)
c/ Å	24.9374(2)	20.8818(5)	22.5410(3)	21.7312(6)
α/ °	90	83.912(2)	98.706(1)	93.165(3)
β/ °	96.330(1)	80.949(2)	91.350(1)	90.606(2)
γ/ °	90	77.803(2)	99.801(1)	114.468(3)
U/ Å^3	5674.92(7)	3011.61(12)	2933.70(8)	2743.44(16)
Z	4	2	2	2
ρ_{calc}/ g cm^{-3}	1.114	1.118	1.143	1.115
μ/ mm^{-1}	0.643	1.271	0.650	0.080
F(000)	2076.0	1104.0	1100.0	1006.0
Crystal size/ mm^3	0.254 × 0.11 × 0.085	0.255 × 0.166 × 0.085	0.229 × 0.214 × 0.095	0.419 × 0.358 × 0.21
2θ range for data collection/°	7.672 to 145.954	7.776 to 145.99	7.25 to 146.252	6.398 to 59.15
Index ranges	-14 ≤ h ≤ 10<br>-24 ≤ k ≤ 24<br>-30 ≤ l ≤ 29	-11 ≤ h ≤ 13<br>-16 ≤ k ≤ 16<br>-25 ≤ l ≤ 25	-11 ≤ h ≤ 13<br>-15 ≤ k ≤ 15<br>-27 ≤ l ≤ 27	-15 ≤ h ≤ 15<br>-16 ≤ k ≤ 15<br>-29 ≤ l ≤ 29
Reflections collected	79287	38857	59379	48452
Independent reflections, R_{int}	11295, 0.0298	11998, 0.0398	11646, 0.0421	14354, 0.0308
Data/restraints/parameters	11295/255/762	11998/211/720	11646/0/680	14354/73/649
Goodness–of–fit on F^2	1.031	1.024	1.046	1.019
Final R_1, wR_2 [I>=2σ(I)]	0.0382, 0.0991	0.0402, 0.1008	0.0448, 0.1207	0.0510, 0.1142
Final R_1, wR_2 [all data]	0.0430, 0.1033	0.0475, 0.1075	0.0473, 0.1234	0.0791, 0.1307
Largest diff. peak/hole/ e Å^{-3}	0.36/-0.22	0.27/-0.24	0.40/-0.24	0.36/-0.23

Computational Details

DFT calculations were run with Gaussian 16 (C.01).[7] The Mg centres were described with the Stuttgart RECPs and associated basis sets,[8] and the 6-31G** basis set was used for all other atoms (BS1).[9] Initial BP86 optimisations were performed using the 'grid = ultrafine' option,[10] with all stationary points being fully characterised via analytical frequency calculations as minima or transition states (all positive eigenvalues or one imaginary eigenvalue respectively). All energies were recomputed with a larger basis set featuring 6-311++G** basis sets on all atoms (BS2). Corrections for the effect of benzene (ε = 2.2706) solvent were run using the polarisable continuum model and BS1.[11] Single-point dispersion corrections to the BP86 results employed Grimme's D3 parameter set with Becke-Johnson damping as implemented in Gaussian.[12] Please note that the structure and energetics of **I** has already been reported previously.[1]

Breakdown of energy contributions

The following table details the evolution of the relative energies as the successive corrections to the initial SCF energy are included. Terms used are:

$\mathbf{\Delta E_{BSI}}$	SCF energy computed with the BP86 functional with BS1
$\mathbf{\Delta H_{BSI}}$	Enthalpy at 0 K with BS1
$\mathbf{\Delta G_{BSI}}$	Free energy at 298.15 K and 1 atm with BS1
$\mathbf{\Delta G_{BSI/C_6H_6}}$	Free energy corrected for C_6H_6 solvent with BS1
$\mathbf{\Delta G_{BSI/C_6H_6+D3BJ}}$	Free energy corrected for C_6H_6 and dispersion effects (D3BJ) with BS1
$\mathbf{\Delta E_{BS2}}$	SCF energy computed with the BP86 functional with BS2
$\mathbf{\Delta G_{C_6H_6}}$	Free energy corrected for BS2, D3BJ and C_6H_6 solvent

In each case the final data used in the main article are highlighted in bold.

Wiberg bond indices were computed with NBO7 at the BP86-D3BJ/6-311++G**//BP86/BS1 level of theory.

Table S2. Relative energies (kcal mol^{-1}) for computed structures. Data in bold are those used in the main text. All energies are quoted relative to **I** at 0.0 kcal/mol.

	ΔE_{BSI}	ΔH_{BSI}	ΔG_{BSI}	$\Delta G_{BSI/C_6H_6}$	$\Delta G_{BSI/C_6H_6+D3BJ}$	ΔE_{BS2}	$\Delta G_{C_6H_6}$
**I**	0.0	0.0	0.0	0.0	0.0	0.0	**0.0**
**TS(I-II)**	6.9	6.5	20.7	22.9	69.4	11.7	**14.1**
**II**	3.9	4.8	18.3	20.1	66.4	9.2	**11.7**
**TS(II-III)**	6.9	7.4	21.5	22.8	69.6	10.3	**13.0**
**III**	-20.8	-19.2	-4.6	-4.3	41.0	-16.6	**-14.8**
**TS(III-IV)**	X	X	X	X	X	X	**X**
**IV**	-29.5	-27.8	-14.1	-12.7	33.5	-24.0	**-21.1**
**TS(IV-V)**	-8.9	-7.9	9.8	11.1	52.5	-2.7	**-1.2**
**V**	-67.1	-63.2	-45.6	-43.7	-2.3	-61.5	**-56.8**
**TS(V-P)**	X	X	X	X	X	X	**X**
**P_M**	-69.1	-66.1	-52.5	-51.7	-5.3	-65.7	**-64.7**
**V_{neo}**	0.0	0.0	0.0	0.0	0.0	0.0	**0.0**
**P_{neo}**	8.8	8.3	6.0	4.8	10.3	7.1	**8.6**

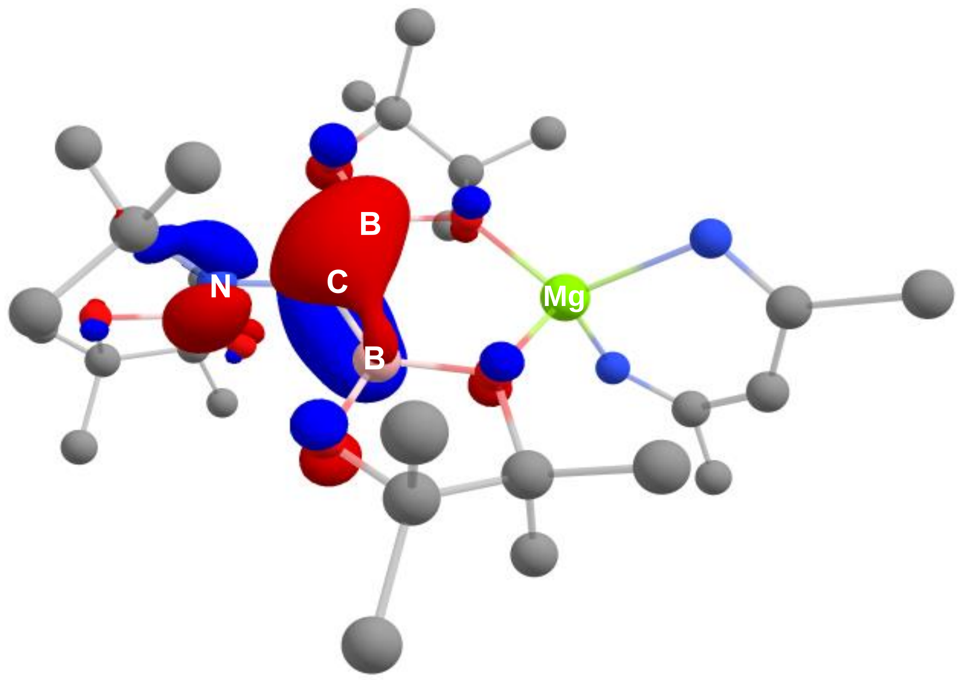

Figure S25. The HOMO of compound **3**, which predominantly features B-C-B π-bonding character, computed at the BP86-D3BJ/6-311++G**//BP86/BS1 level of theory.

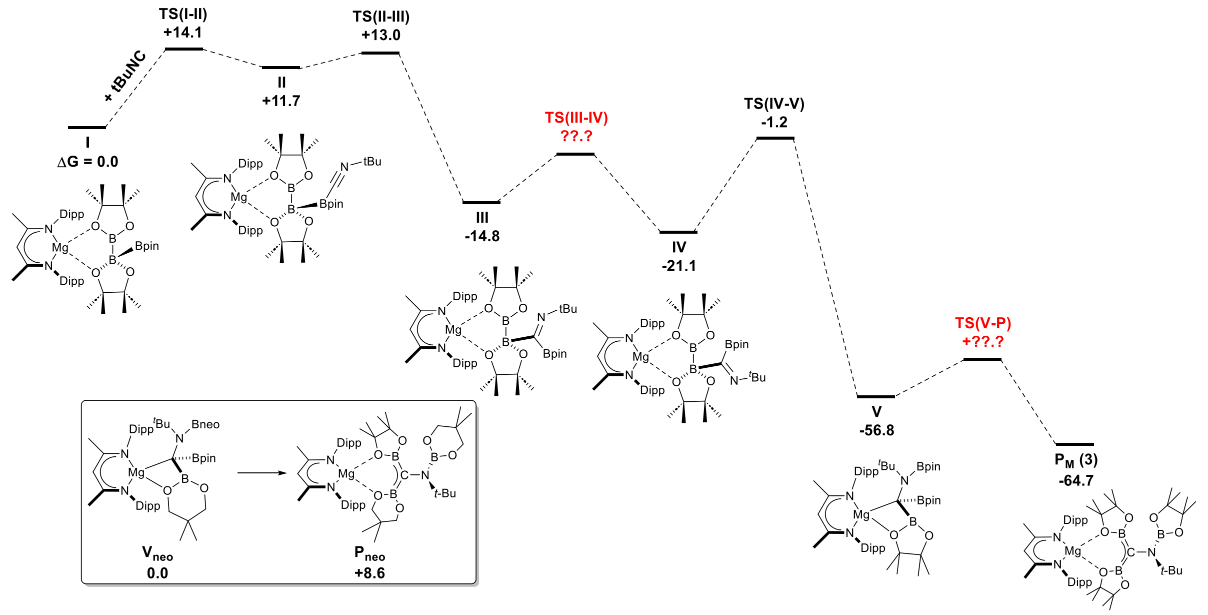

Figure S26. Free energy profile (calculated with DFT at the BP86-D3BJ(PCM=Benzene)/BS2//BS2586/BS1 level of theory, energies in kcal mol^{-1}) of ^{t}BuNC to **I** to form **P$_M$**. *Inset*: Relative difference in free energies between **V$_{neo}$** (crystalographically observed) and **P$_{neo}$**.

Cartesian Coordinates and Computed Energies (in Hartrees) for Calculated Structures

***t*-BuNC**

SCF (BP86) Energy = -250.657661497
Enthalpy 0K = -250.531377
Enthalpy 298K = -250.522865
Free Energy 298K = -250.561851
Lowest Frequency = 164.9159 cm^{-1}
Second Frequency = 164.9507 cm^{-1}
SCF (BP86-D3BJ) Energy = -250.677045708
SCF (C6H6) Energy = -250.660438790
SCF (BS2) Energy = -250.723756249

```
C   2.38157 -0.00001  0.00002
N   1.19018  0.00001 -0.00002
C  -0.25779  0.00000 -0.00003
C  -0.73920  0.14366  1.46159
H  -0.37417  1.08539  1.90209
H  -0.37377 -0.69406  2.07707
H  -1.84153  0.14627  1.49071
C  -0.73919  1.19397 -0.85521
H  -0.37413  2.14585 -0.43716
H  -1.84153  1.21763 -0.87239
H  -0.37379  1.10483 -1.89090
C  -0.73920 -1.33763 -0.60638
H  -0.37358 -1.45193 -1.63952
H  -1.84154 -1.36411 -0.61888
H  -0.37432 -2.18991 -0.01089
```

TS(I-II)

SCF (BP86) Energy = -2724.63106023
Enthalpy 0K = -2723.355020
Enthalpy 298K = -2723.277715
Free Energy 298K = -2723.463843
Lowest Frequency = -151.0554 cm^{-1}
Second Frequency = -9.8888 cm^{-1}
SCF (BP86-D3BJ) Energy = -2725.02258476
SCF (C6H6) Energy = -2724.63674821
SCF (BS2) Energy = -2924.53955766

```
Mg -1.16288 -0.06332  0.35700
O   0.33808 -0.22699  1.86221
O   2.44328  0.66548  2.15952
O   0.23547 -0.38268 -1.01012
O   1.80540  1.33219 -1.37086
O   3.54263 -1.55332 -1.90356
N  -2.29673  1.70073  0.66235
N  -2.79767 -1.34325  0.65116
C  -4.41809 -1.99982  2.42775
H  -5.39214 -2.09529  1.91869
H  -4.61665 -1.69070  3.46551
H  -3.95736 -3.00009  2.42644
C  -3.53185 -0.97977  1.72538
C  -3.56590  0.33651  2.26067
H  -4.16566  0.43394  3.16989
C  -3.14853  1.57876  1.70353
C  -3.69779  2.81578  2.40648
H  -2.96859  3.16194  3.16163
H  -4.64124  2.59500  2.92791
H  -3.85785  3.64980  1.70846
C  -3.10818 -2.59989  0.00651
C  -2.26564 -3.73197  0.20479
C  -2.54388 -4.92891 -0.48260
H  -1.88735 -5.79279 -0.32944
C  -3.64089 -5.03856 -1.34179
H  -3.84513 -5.97771 -1.86670
C  -4.47811 -3.93244 -1.51614
H  -5.34445 -4.01568 -2.18221
C  -4.23990 -2.70828 -0.86001
C  -1.07606 -3.69671  1.15698
H  -1.06065 -2.69981  1.62882
C  -1.21535 -4.74194  2.28717
H  -2.14894 -4.60606  2.86029
H  -0.36829 -4.66726  2.99138
H  -1.21911 -5.77030  1.88521
C   0.25424 -3.87495  0.39995
H   0.30982 -4.87348 -0.07090
H   1.11394 -3.76731  1.07980
H   0.37222 -3.11352 -0.38734
C  -5.21365 -1.55716 -1.12641
H  -4.88068 -0.68171 -0.54261
C  -6.65737 -1.90349 -0.68924
H  -7.07109 -2.72829 -1.29582
H  -7.31994 -1.03004 -0.81997
H  -6.71266 -2.21475  0.36708
C  -5.21379 -1.16336 -2.62087
H  -4.21760 -0.83218 -2.95597
H  -5.93034 -0.34390 -2.80441
H  -5.51535 -2.01362 -3.25721
C  -2.19190  2.99904  0.02354
C  -1.17426  3.92728  0.39146
C  -1.13095  5.18404 -0.24426
H  -0.35001  5.89547  0.04636
C  -2.05930  5.54172 -1.22437
H  -2.01451  6.52699 -1.70039
C  -3.12483  3.34357 -1.00538
C  -3.04054  4.61849 -1.59942
H  -3.76027  4.89111 -2.37756
C  -0.12782  3.62960  1.46145
H  -0.29146  2.59042  1.79302
C  -0.28297  4.55878  2.68830
H  -0.09339  5.61086  2.41091
H   0.44606  4.29125  3.47437
H  -1.29373  4.51121  3.12699
C   1.30870  3.72200  0.90091
H   1.44579  3.05429  0.03223
H   2.03204  3.42286  1.67915
H   1.54690  4.75696  0.59448
C  -4.22273  2.38433 -1.47519
H  -3.89883  1.36156 -1.20914
C  -5.56690  2.64914 -0.75547
H  -5.48760  2.50603  0.33368
H  -6.34773  1.96288 -1.12902
H  -5.91034  3.68299 -0.93898
C  -4.43181  2.43971 -3.00498
H  -4.88433  3.39510 -3.32352
H  -5.11742  1.63880 -3.32540
H  -3.48234  2.31775 -3.55160
C   0.14342 -0.09132 -2.44001
C   1.16898  1.14117 -2.64308
C   0.52124 -1.36911 -3.20727
H   1.54145 -1.69174 -2.95634
H   0.45480 -1.21484 -4.29798
H  -0.18337 -2.17413 -2.93551
C  -1.32307  0.24740 -2.74516
H  -1.96197 -0.61947 -2.49469
H  -1.46843  0.45813 -3.81816
H  -1.67747  1.12897 -2.18517
C   0.46686  2.47115 -2.98802
H  -0.26771  2.76538 -2.22232
H  -0.03671  2.43254 -3.97021
H   1.22877  3.26728 -3.03490
C   2.23561  0.85932 -3.72579
H   2.88471  1.74642 -3.81822
H   1.77987  0.67931 -4.71622
H   2.86479 -0.00152 -3.45500
C   0.55548 -0.24353  3.34703
C   2.11113  0.06076  3.45050
C  -0.34007  0.82960  3.97545
H  -0.07309  1.84243  3.64169
H  -0.26057  0.79896  5.07543
H  -1.39227  0.63724  3.70394
C   0.14679 -1.61959  3.87634
H  -0.93831 -1.76723  3.74957
H   0.36991 -1.69240  4.95509
H   0.67554 -2.43078  3.35636
C   2.48633  1.06039  4.55221
H   3.57140  1.24762  4.51219
H   2.24853  0.65729  5.55183
H   1.97093  2.02489  4.43070
```

```
C   2.98575 -1.20070  3.57072
H   2.83053 -1.87286  2.71179
H   2.80279 -1.73969  4.51576
H   4.04349 -0.89184  3.55107
B   1.51153  0.30412  1.20817
B   1.66158  0.12702 -0.51213
B   2.99914 -1.08846 -0.65467
O   3.15045 -2.14120  0.30705
C   4.19444 -2.83848 -1.71770
C   3.33641 -3.89033 -2.44763
H   3.23929 -3.59950 -3.50703
H   3.79186 -4.89520 -2.40898
H   2.32391 -3.94478 -2.01722
C   5.58329 -2.77284 -2.37317
H   6.13730 -3.71950 -2.24268
H   5.46979 -2.59358 -3.45562
H   6.18764 -1.95284 -1.95645
C   4.21445 -3.02717 -0.13829
C   3.90145 -4.45547  0.33335
H   4.68458 -5.15960  0.00064
H   3.87528 -4.48189  1.43622
H   2.93097 -4.81302 -0.03883
C   5.53375 -2.57988  0.52616
H   5.42218 -2.65144  1.62114
H   6.38117 -3.21897  0.22469
H   5.77706 -1.53444  0.28192
C   4.39619  0.31059 -0.24694
N   4.93222  1.35221 -0.42539
C   5.49167  2.66456 -0.66554
C   6.98172  2.49328 -1.03753
H   7.09002  1.86863 -1.93874
H   7.54294  2.02285 -0.21361
H   7.42405  3.48229 -1.24247
C   4.69739  3.31018 -1.82258
H   4.88117  2.76923 -2.76468
H   5.01556  4.35868 -1.94930
H   3.61815  3.26726 -1.60997
C   5.34369  3.48702  0.63432
H   4.28229  3.59453  0.90544
H   5.77597  4.49023  0.48344
H   5.87211  2.99811  1.46878
```

II

SCF (BP86) Energy = -2724.63588774
Enthalpy 0K = -2723.358357
Enthalpy 298K = -2723.280345
Free Energy 298K = -2723.467662
Lowest Frequency = 9.2537 cm^{-1}
Second Frequency = 17.3857 cm^{-1}
SCF (BP86-D3BJ) Energy = -2725.02760555
SCF (C6H6) Energy = -2724.64216163
SCF (BS2) Energy = -2924.54367272

```
Mg -1.08720 -0.05164  0.35145
O   0.44436  0.06876  1.82570
O   2.46312  1.17317  2.00609
O   0.25873 -0.06752 -1.10912
O   1.75154  1.71502 -1.45002
O   3.30620 -1.37421 -2.06289
N  -2.52281  1.45801  0.70628
N  -2.41184 -1.64784  0.70225
C  -4.06820 -2.56403  2.33665
H  -4.18600 -3.40801  1.64182
H  -5.06210 -2.22168  2.66329
H  -3.53913 -2.94357  3.22924
C  -3.27683 -1.42148  1.70988
C  -3.52948 -0.13822  2.27750
H  -4.16804 -0.16753  3.16530
C  -3.31125  1.16828  1.76819
C  -4.01349  2.28569  2.53512
H  -3.34825  2.64088  3.34428
H  -4.94571  1.93428  3.00296
H  -4.23487  3.15060  1.89437
C  -2.36255 -2.95434  0.07636
C  -1.59239 -4.01381  0.63853
C  -1.51050 -5.23956 -0.05259
H  -0.91254 -6.05051  0.37800
C  -2.17575 -5.44463 -1.26310
H  -2.09699 -6.40404 -1.78505
C  -2.95257 -4.41053 -1.79573
H  -3.48934 -4.57423 -2.73516
C  -3.06437 -3.16369 -1.15073
C  -0.86277 -3.89537  1.97531
H  -1.07842 -2.89609  2.38741
C  -1.35640 -4.95079  2.99409
H  -2.45152 -4.92904  3.11944
H  -0.89248 -4.77865  3.98142
H  -1.08109 -5.97170  2.67618
C   0.66391 -4.01638  1.79202
H   0.92153 -4.97868  1.31602
H   1.17968 -3.99636  2.76909
H   1.08115 -3.20792  1.16925
C  -3.99380 -2.10299 -1.74138
H  -3.64255 -1.11820 -1.38218
C  -5.43734 -2.29264 -1.21493
H  -5.82715 -3.28493 -1.50394
H  -6.11211 -1.52680 -1.63681
H  -5.48528 -2.21771 -0.11661
C  -3.99307 -2.08169 -3.28454
H  -2.97046 -2.02752 -3.69362
H  -4.55651 -1.20858 -3.65266
H  -4.47853 -2.97868 -3.70754
C  -2.72371  2.72225  0.02922
C  -1.81421  3.80836  0.19624
C  -2.02575  4.99906 -0.52744
H  -1.32483  5.83011 -0.39234
C  -3.11497  5.14949 -1.39000
H  -3.26561  6.08581 -1.93760
C  -3.84048  2.86907 -0.85528
C  -4.01325  4.08815 -1.53948
H  -4.87305  4.20347 -2.20847
C  -0.66691  3.76695  1.20164
H  -0.60056  2.72815  1.56612
C  -0.98141  4.68176  2.41052
H  -1.04751  5.73719  2.09193
H  -0.18295  4.61694  3.17178
H  -1.93849  4.42058  2.89200
C   0.70040  4.13888  0.59181
H   0.98091  3.45403 -0.22685
H   1.48206  4.07525  1.36936
H   0.70397  5.17485  0.20763
C  -4.87365  1.76082 -1.07953
H  -4.47602  0.83498 -0.62932
C  -6.21731  2.08566 -0.38343
H  -6.09741  2.22699  0.70231
H  -6.94292  1.26823 -0.54104
H  -6.65817  3.01125 -0.79491
C  -5.12006  1.49184 -2.58078
H  -5.58504  2.35904 -3.08142
H  -5.80989  0.63918 -2.70687
H  -4.18270  1.26061 -3.11199
C   0.09685  0.29608 -2.52340
C   1.16064  1.49265 -2.74016
C   0.37143 -0.95910 -3.36488
H   1.37954 -1.34780 -3.16085
H   0.27322 -0.74389 -4.44326
H  -0.36490 -1.73978 -3.10519
C  -1.36296  0.72457 -2.72270
H  -2.03249 -0.12011 -2.48180
H  -1.55581  0.99398 -3.77497
H  -1.64288  1.58879 -2.09999
C   0.51032  2.82524 -3.16630
H  -0.23927  3.17621 -2.44049
H   0.03460  2.75145 -4.15991
H   1.29771  3.59508 -3.23052
C   2.26117  1.14762 -3.76995
H   2.95763  2.00118 -3.83233
H   1.84046  0.98955 -4.77918
H   2.83126  0.25310 -3.47800
C   0.69469  0.19915  3.30158
C   2.22960  0.58349  3.32834
C  -0.23327  1.28892  3.84855
H  -0.00502  2.27829  3.42952
H  -0.14529  1.35053  4.94646
```

```
H  -1.27833  1.03271  3.60563
C   0.36421 -1.12893  3.97921
H  -0.71923 -1.32408  3.91509
H   0.63080 -1.08057  5.04965
H   0.90587 -1.96697  3.52217
C   2.59636  1.62830  4.38916
H   3.67008  1.86271  4.30978
H   2.40995  1.24054  5.40567
H   2.03344  2.56499  4.26143
C   3.17016 -0.63048  3.43418
H   3.00209 -1.33748  2.60522
H   3.06532 -1.14646  4.40371
H   4.20932 -0.27264  3.34935
B   1.53285  0.68551  1.10653
B   1.63704  0.50292 -0.60758
B   3.05268 -0.71573 -0.77025
O   3.16096 -1.77206  0.24452
C   3.84552 -2.69662 -1.83313
C   2.77108 -3.71380 -2.26923
H   2.50607 -3.52419 -3.32285
H   3.12716 -4.75620 -2.19110
H   1.85672 -3.60262 -1.66487
C   5.09912 -2.87397 -2.70689
H   5.57225 -3.85921 -2.54434
H   4.81730 -2.80638 -3.77134
H   5.84509 -2.08942 -2.50670
C   4.12261 -2.73075 -0.26852
C   3.87308 -4.09626  0.38654
H   4.58295 -4.84915  0.00055
H   4.02376 -4.02010  1.47705
H   2.85252 -4.45831  0.20599
C   5.55239 -2.28185  0.11259
H   5.61533 -2.19360  1.21042
H   6.30875 -3.01383 -0.21821
H   5.81368 -1.30404 -0.32061
C   4.24620  0.43933 -0.58316
N   4.79663  1.48834 -0.53611
C   5.27129  2.85413 -0.46909
C   6.78552  2.85975 -0.77829
H   6.98126  2.45538 -1.78462
H   7.34148  2.25604 -0.04237
H   7.16373  3.89477 -0.73911
C   4.48692  3.68107 -1.51243
H   4.75081  3.36071 -2.53370
H   4.74345  4.74832 -1.40280
H   3.40483  3.53214 -1.37596
C   5.00278  3.36663  0.96418
H   3.93415  3.28201  1.21150
H   5.31602  4.42143  1.03700
H   5.57482  2.77834  1.70011
```

TS(II-III)

SCF (BP86) Energy = -2724.63112051
Enthalpy 0K = -2723.353828
Enthalpy 298K = -2723.276265
Free Energy 298K = -2723.462553
Lowest Frequency = -35.7126 cm^{-1}
Second Frequency = 15.7639 cm^{-1}
SCF (BP86-D3BJ) Energy = -2725.02192268
SCF (C6H6) Energy = -2724.63828972
SCF (BS2) Energy = -2924.54190770

```
Mg  1.08134  0.06748  0.40848
O  -0.55823 -0.08078  1.73086
O  -2.22052 -1.69708  1.88009
O  -0.20243 -0.07067 -1.16203
O  -1.53070 -1.92230 -1.64658
O  -3.27654  1.38395 -1.92059
N   2.54465 -1.41287  0.75001
N   2.35185  1.70016  0.74996
C   4.00852  2.64919  2.36925
H   4.04396  3.52403  1.70464
H   5.03593  2.34487  2.62230
H   3.51871  2.96323  3.30842
C   3.23741  1.49093  1.74636
C   3.52713  0.21519  2.30678
H   4.17257  0.25879  3.18953
C   3.33575 -1.09819  1.80228
C   4.07672 -2.18638  2.57475
H   3.51373 -2.40653  3.50078
H   5.08203 -1.85534  2.87887
H   4.16222 -3.12028  2.00330
C   2.26276  3.00502  0.12485
C   1.47590  4.04659  0.69793
C   1.33908  5.26222 -0.00293
H   0.72831  6.05949  0.43494
C   1.96845  5.47562 -1.23095
H   1.84479  6.42581 -1.76086
C   2.77414  4.46560 -1.76725
H   3.29122  4.64105 -2.71550
C   2.94323  3.23058 -1.11202
C   0.79988  3.92966  2.06376
H   1.04308  2.93502  2.47528
C   1.32585  4.99936  3.05160
H   2.42486  4.98672  3.13335
H   0.90265  4.83415  4.05808
H   1.02993  6.01420  2.73317
C  -0.73429  4.03594  1.94876
H  -1.02320  4.98677  1.46771
H  -1.20279  4.01989  2.94797
H  -1.16419  3.21066  1.35827
C   3.92149  2.20894 -1.69363
H   3.61512  1.21016 -1.33113
C   5.35083  2.46806 -1.15687
H   5.69529  3.47647 -1.44730
H   6.06358  1.73312 -1.57077
H   5.39396  2.40000 -0.05818
C   3.93880  2.18223 -3.23644
H   2.92516  2.08264 -3.65909
H   4.54490  1.33372 -3.59427
H   4.39002  3.09810 -3.65665
C   2.79500 -2.66296  0.06014
C   1.89886 -3.76939  0.16938
C   2.17033 -4.94259 -0.56365
H   1.48181 -5.78897 -0.47478
C   3.29978 -5.05770 -1.37885
H   3.49358 -5.98257 -1.93230
C   3.95163 -2.77229 -0.77907
C   4.18002 -3.97619 -1.47398
H   5.06982 -4.06207 -2.10731
C   0.70257 -3.77756  1.11918
H   0.48284 -2.72809  1.38483
C   1.06160 -4.53353  2.42204
H   1.29964 -5.58924  2.20077
H   0.20914 -4.52524  3.12481
H   1.93580 -4.09649  2.93248
C  -0.57866 -4.37498  0.50106
H  -0.83976 -3.88381 -0.44895
H  -1.42440 -4.22167  1.19077
H  -0.47894 -5.46164  0.32820
C   4.97190 -1.64234 -0.95569
H   4.56002 -0.73407 -0.48344
C   6.31416 -1.97279 -0.25940
H   6.18536 -2.17160  0.81588
H   7.02453 -1.13445 -0.36887
H   6.77897 -2.86778 -0.71005
C   5.23247 -1.32590 -2.44587
H   5.71145 -2.17399 -2.96541
H   5.91479 -0.46292 -2.53921
H   4.29983 -1.08924 -2.98249
C   0.03019 -0.33809 -2.60574
C  -1.00240 -1.52199 -2.93350
C  -0.24232  0.96863 -3.35709
H  -1.26100  1.32649 -3.14996
H  -0.11677  0.82613 -4.44436
H   0.47365  1.74215 -3.02962
C   1.50033 -0.74001 -2.75555
H   2.14666  0.09288 -2.42952
H   1.74617 -0.94420 -3.81102
H   1.75464 -1.63523 -2.16729
C  -0.34769 -2.77281 -3.54799
H   0.43427 -3.19483 -2.89843
H   0.08820 -2.55706 -4.53861
H  -1.12447 -3.54303 -3.68164
```

```
C  -2.18442 -1.08938 -3.82338
H  -2.87859 -1.94118 -3.90850
H  -1.84617 -0.82346 -4.84027
H  -2.73446 -0.23821 -3.39436
C  -0.82828 -0.24990  3.19404
C  -2.20054 -1.04553  3.18579
C   0.34696 -1.02743  3.79817
H   0.44550 -2.03268  3.36379
H   0.21889 -1.13335  4.88846
H   1.28751 -0.47667  3.62091
C  -0.92456  1.12849  3.84460
H   0.05624  1.63086  3.82744
H  -1.22464  1.02192  4.90168
H  -1.65886  1.76468  3.33398
C  -2.29395 -2.13962  4.25749
H  -3.25539 -2.66649  4.14939
H  -2.25709 -1.70477  5.27130
H  -1.48724 -2.88211  4.16259
C  -3.44481 -0.14054  3.25383
H  -3.45072  0.61358  2.44876
H  -3.52953  0.36162  4.23288
H  -4.33593 -0.77459  3.11833
B  -1.43954 -0.98131  0.99536
B  -1.31052 -0.94043 -0.67571
B  -3.35587  0.67596 -0.66578
O  -3.50734  1.63923  0.40051
C  -3.67438  2.76171 -1.67568
C  -2.41923  3.64994 -1.75457
H  -1.94668  3.52571 -2.74274
H  -2.66489  4.71922 -1.63119
H  -1.68276  3.36465 -0.98733
C  -4.66815  3.17107 -2.77353
H  -5.05465  4.19215 -2.60493
H  -4.16245  3.15940 -3.75394
H  -5.51912  2.47533 -2.82450
C  -4.29165  2.70379 -0.21669
C  -4.13646  3.98621  0.60558
H  -4.69883  4.81560  0.14175
H  -4.54347  3.82885  1.61884
H  -3.08541  4.28951  0.70101
C  -5.77463  2.27758 -0.21137
H  -6.09109  2.10821  0.83168
H  -6.42759  3.05099 -0.64968
H  -5.91581  1.33525 -0.76544
C  -4.01575 -0.67507 -0.58765
N  -4.14979 -1.89452 -0.43743
C  -5.42547 -2.65903 -0.50363
C  -6.63926 -1.77642 -0.85263
H  -6.49427 -1.28509 -1.82964
H  -6.77847 -0.98805 -0.09409
H  -7.56090 -2.38258 -0.89992
C  -5.21635 -3.74661 -1.57885
H  -5.07770 -3.28729 -2.57207
H  -6.09122 -4.41835 -1.62460
H  -4.31840 -4.34269 -1.34973
C  -5.60862 -3.31409  0.88178
H  -4.70927 -3.88953  1.15370
H  -6.48087 -3.99077  0.87707
H  -5.76977 -2.54432  1.65545
```

III

SCF (BP86) Energy = -2724.67521934
Enthalpy 0K = -2723.396171
Enthalpy 298K = -2723.318623
Free Energy 298K = -2723.504253
Lowest Frequency = 14.9653 cm^{-1}
Second Frequency = 20.9874 cm^{-1}
SCF (BP86-D3BJ) Energy = -2725.06848713
SCF (C6H6) Energy = -2724.68372156
SCF (BS2) Energy = -2924.58476900

```
Mg  1.08462  0.09016  0.35257
O   0.08990 -0.97160  1.88268
O  -1.31521 -2.78731  2.03954
O  -0.38411 -0.26738 -0.97623
O  -0.65305 -2.54107 -1.49806
O  -4.30616  0.07559 -2.04379
N   3.10076 -0.58010  0.32497
N   1.52873  2.04165  1.01536
C   2.78843  3.33707  2.74620
H   2.46080  4.24887  2.22607
H   3.85388  3.43223  3.00425
H   2.22081  3.27923  3.69208
C   2.53978  2.08652  1.90912
C   3.44275  1.01565  2.16167
H   4.11904  1.20144  3.00154
C   3.80185 -0.11828  1.38389
C   5.07326 -0.82513  1.85009
H   4.83769 -1.44482  2.73522
H   5.84571 -0.10285  2.15745
H   5.48709 -1.48826  1.07864
C   0.80714  3.26249  0.71412
C  -0.26289  3.71999  1.54041
C  -0.94217  4.90301  1.18612
H  -1.75819  5.25569  1.82708
C  -0.59453  5.63697  0.04874
H  -1.13357  6.55537 -0.20654
C   0.45723  5.18609 -0.75512
H   0.74137  5.76492 -1.63974
C   1.17111  4.01150 -0.44668
C  -0.69783  2.99508  2.81237
H  -0.01418  2.14278  2.94985
C  -0.59687  3.89992  4.06372
H   0.40025  4.35737  4.17088
H  -0.80858  3.31756  4.97790
H  -1.33324  4.72176  4.02226
C  -2.13465  2.44043  2.69083
H  -2.86127  3.26588  2.58041
H  -2.41821  1.88843  3.60552
H  -2.26347  1.77081  1.82579
C   2.36578  3.62315 -1.31822
H   2.56325  2.54888 -1.14920
C   3.63243  4.39540 -0.87617
H   3.47922  5.48442 -0.97948
H   4.50021  4.11425 -1.49857
H   3.88904  4.18881  0.17560
C   2.11083  3.83482 -2.82558
H   1.17958  3.34461 -3.15562
H   2.94446  3.41828 -3.41469
H   2.03695  4.90554 -3.08457
C   3.77479 -1.45625 -0.61379
C   3.51025 -2.85871 -0.64087
C   4.14173 -3.65498 -1.61744
H   3.93624 -4.73055 -1.63270
C   5.03355 -3.11006 -2.54545
H   5.51709 -3.74840 -3.29242
C   4.69139 -0.89231 -1.55829
C   5.30588 -1.73935 -2.50126
H   6.01295 -1.30919 -3.21910
C   2.65388 -3.55169  0.41618
H   2.12317 -2.75687  0.96643
C   3.56725 -4.30286  1.41678
H   4.12567 -5.10393  0.90082
H   2.96935 -4.77924  2.21429
H   4.30563 -3.63586  1.89199
C   1.59420 -4.51317 -0.16086
H   0.88582 -3.98822 -0.82227
H   1.01054 -4.96028  0.66321
H   2.05929 -5.34756 -0.71593
C   5.05401  0.59576 -1.58381
H   4.36547  1.12439 -0.90239
C   6.49702  0.83526 -1.07795
H   6.64691  0.44666 -0.05834
H   6.73144  1.91439 -1.06985
H   7.23094  0.33656 -1.73584
C   4.89174  1.20860 -2.99294
H   5.59926  0.76503 -3.71496
H   5.09713  2.29308 -2.96432
H   3.87375  1.05923 -3.38728
C  -0.15897 -0.37456 -2.43037
C  -0.53621 -1.90492 -2.77273
C  -1.03159  0.66922 -3.14266
H  -2.10064  0.52948 -2.93060
H  -0.89034  0.61249 -4.23634
```

```
H  -0.73636  1.68199 -2.81630
C   1.31248 -0.03422 -2.69643
H   1.53152  0.98786 -2.33567
H   1.53628 -0.04415 -3.77642
H   2.00460 -0.74175 -2.21461
C   0.56087 -2.65252 -3.55707
H   1.51553 -2.68379 -3.01046
H   0.73263 -2.20240 -4.55057
H   0.23156 -3.69333 -3.71156
C  -1.85597 -2.05001 -3.56902
H  -2.09294 -3.12401 -3.63735
H  -1.75772 -1.65308 -4.59536
H  -2.70945 -1.55251 -3.08830
C   0.17448 -1.42491  3.31623
C  -1.10509 -2.34192  3.41474
C   1.48818 -2.18944  3.49778
H   1.51391 -3.10706  2.89457
H   1.62478 -2.46687  4.55650
H   2.33139 -1.54452  3.20233
C   0.16555 -0.20591  4.23493
H   1.07376  0.39742  4.06715
H   0.17239 -0.53675  5.28831
H  -0.71902  0.42538  4.07855
C  -0.93335 -3.58230  4.29958
H  -1.86408 -4.17135  4.27902
H  -0.73246 -3.29758  5.34697
H  -0.11756 -4.22942  3.94559
C  -2.37778 -1.57863  3.82688
H  -2.53741 -0.68837  3.19767
H  -2.34456 -1.26704  4.88478
H  -3.24406 -2.24470  3.68909
B  -0.77670 -1.87559  1.16314
B  -1.16280 -1.57956 -0.51492
B  -3.67558 -0.14705 -0.81953
O  -3.81361  0.93168  0.04317
C  -5.09782  1.30753 -1.94997
C  -4.94302  2.07288 -3.27043
H  -5.34099  1.46057 -4.09614
H  -5.51057  3.01918 -3.24480
H  -3.89089  2.30227 -3.49331
C  -6.56810  0.88460 -1.77912
H  -7.23733  1.76090 -1.74758
H  -6.86230  0.25771 -2.63651
H  -6.72423  0.29408 -0.86290
C  -4.47818  2.03007 -0.67704
C  -3.39507  3.06113 -1.02594
H  -3.82488  3.93629 -1.54225
H  -2.90878  3.41341 -0.10361
H  -2.61431  2.62378 -1.66644
C  -5.50956  2.65523  0.27025
H  -4.99264  3.09019  1.14105
H  -6.06589  3.46461 -0.23339
H  -6.23085  1.91138  0.64033
C  -2.84163 -1.46766 -0.51522
N  -3.31528 -2.64861 -0.23231
C  -4.75988 -2.99752 -0.12601
C  -5.54922 -2.03367  0.78665
H  -5.54392 -0.99825  0.40997
H  -5.10797 -2.01683  1.79830
H  -6.60255 -2.35478  0.87597
C  -5.38169 -3.06913 -1.53975
H  -5.39686 -2.07845 -2.01995
H  -6.41465 -3.45912 -1.49161
H  -4.78777 -3.74821 -2.17490
C  -4.78418 -4.40748  0.50938
H  -4.22593 -5.11595 -0.12447
H  -5.81808 -4.77731  0.63429
H  -4.29078 -4.38765  1.49565
```

TS(III-IV)

XXXX

IV

SCF (BP86) Energy = -2724.68912923
Enthalpy 0K = -2723.409818
Enthalpy 298K = -2723.332272
Free Energy 298K = -2723.519357
Lowest Frequency = 7.4895 cm^{-1}
Second Frequency = 16.3218 cm^{-1}
SCF (BP86-D3BJ) Energy = -2725.08108510
SCF (C6H6) Energy = -2724.69600399
SCF (BS2) Energy = -2924.59654978

```
Mg  1.26666  0.03270  0.34862
O  -0.21863  0.00200  1.87296
O  -2.31882 -0.89530  2.16755
O  -0.14242  0.08228 -1.03782
O  -1.82478 -1.53046 -1.32056
O  -4.77182 -0.15036  0.56949
N   2.62812 -1.54610  0.66396
N   2.66192  1.55644  0.63263
C   4.36580  2.42896  2.22931
H   3.75615  3.01488  2.93976
H   4.70820  3.13115  1.45378
H   5.23892  2.03754  2.77175
C   3.53671  1.30145  1.62737
C   3.75502  0.01099  2.19135
H   4.41827  0.01306  3.06109
C   3.46728 -1.28863  1.69221
C   4.15973 -2.42937  2.43340
H   3.58337 -2.66808  3.34678
H   5.17553 -2.14854  2.75199
H   4.21100 -3.34416  1.82710
C   2.67108  2.86110  0.00107
C   1.92264  3.94603  0.54300
C   1.93415  5.18561 -0.12566
H   1.35327  6.01675  0.28941
C   2.66700  5.37664 -1.29966
H   2.66622  6.34932 -1.80271
C   3.39843  4.30769 -1.82742
H   3.97392  4.45500 -2.74689
C   3.41248  3.04377 -1.20551
C   1.09644  3.81801  1.81983
H   1.27567  2.80906  2.22485
C   1.51265  4.84729  2.89663
H   2.59378  4.81337  3.11287
H   0.96793  4.66160  3.83932
H   1.27357  5.87727  2.57858
C  -0.41251  3.95023  1.51836
H  -0.63931  4.95977  1.13192
H  -1.00944  3.81948  2.44003
H  -0.75854  3.22065  0.76532
C   4.26020  1.92650 -1.81594
H   3.87375  0.96812 -1.42250
C   5.73717  2.04528 -1.36917
H   6.16356  3.01238 -1.69028
H   6.34827  1.24111 -1.81606
H   5.83994  1.97764 -0.27386
C   4.17279  1.88247 -3.35678
H   3.12681  1.85473 -3.70518
H   4.68755  0.98706 -3.74255
H   4.65828  2.75835 -3.82158
C   2.72722 -2.81908 -0.01830
C   1.73765 -3.83150  0.16084
C   1.83330 -5.02421 -0.58362
H   1.07149 -5.79805 -0.44003
C   2.88494 -5.24892 -1.47651
H   2.94450 -6.18536 -2.04105
C   3.80826 -3.04368 -0.92980
C   3.86414 -4.26312 -1.63299
H   4.69575 -4.43820 -2.32439
C   0.63385 -3.71699  1.20873
H   0.64177 -2.67363  1.56611
C   0.95781 -4.63586  2.41273
H   0.97191 -5.69430  2.09790
H   0.19150 -4.53744  3.20271
H   1.94152 -4.40800  2.85633
C  -0.77962 -4.02015  0.66997
H  -1.06627 -3.33729 -0.14786
H  -1.52049 -3.89724  1.47942
H  -0.86242 -5.06098  0.30880
C   4.92172 -2.02070 -1.17319
H   4.62934 -1.07728 -0.68101
C   6.26313 -2.48309 -0.55475
```

```
H   6.17897 -2.66391  0.52848
H   7.04617 -1.72074 -0.71298
H   6.60813 -3.42180 -1.02380
C   5.11892 -1.72835 -2.67749
H   5.47461 -2.61942 -3.22363
H   5.87833 -0.93915 -2.81647
H   4.18241 -1.39546 -3.15328
C  -0.06498 -0.28840 -2.45685
C  -1.27036 -1.35282 -2.63798
C  -0.20743  1.00084 -3.28441
H  -1.10641  1.55949 -2.98980
H  -0.23537  0.78380 -4.36608
H   0.66078  1.65388 -3.08878
C   1.32632 -0.88587 -2.70443
H   2.10150 -0.13306 -2.46972
H   1.45724 -1.15520 -3.76612
H   1.51614 -1.78760 -2.10256
C  -0.80571 -2.74559 -3.10976
H  -0.07188 -3.19512 -2.42368
H  -0.36977 -2.71218 -4.12341
H  -1.68393 -3.41162 -3.14198
C  -2.35714 -0.86226 -3.61875
H  -3.18924 -1.58289 -3.61255
H  -1.96705 -0.78908 -4.64964
H  -2.77129  0.11392 -3.33150
C  -0.40670 -0.10681  3.36376
C  -1.97095 -0.29559  3.45645
C   0.39134 -1.31505  3.86126
H   0.00430 -2.25985  3.45656
H   0.35372 -1.36932  4.96233
H   1.44702 -1.20470  3.56265
C   0.12331  1.15945  4.03350
H   1.21692  1.22473  3.90662
H  -0.08529  1.11878  5.11695
H  -0.33777  2.06918  3.62689
C  -2.43267 -1.24257  4.56945
H  -3.53101 -1.32506  4.54202
H  -2.14642 -0.85444  5.56220
H  -2.01271 -2.25254  4.45099
C  -2.75050  1.02998  3.53268
H  -2.41993  1.73426  2.75232
H  -2.64585  1.51318  4.51888
H  -3.81453  0.81559  3.34944
B  -1.39948 -0.50913  1.22051
B  -1.54720 -0.32860 -0.50862
B  -4.17597  0.14609 -0.65049
O  -4.89854 -0.35437 -1.72762
C  -5.78481 -1.18188  0.31021
C  -5.08985 -2.53520  0.54150
H  -4.66807 -2.54452  1.55869
H  -5.79334 -3.37914  0.43912
H  -4.25437 -2.67128 -0.16483
C  -6.93750 -0.98795  1.29891
H  -7.74744 -1.71191  1.10280
H  -6.57592 -1.15595  2.32679
H  -7.35531  0.02886  1.24570
C  -6.14680 -0.92160 -1.20421
C  -6.47760 -2.17758 -2.01682
H  -7.37881 -2.67600 -1.61906
H  -6.67757 -1.90013 -3.06496
H  -5.64414 -2.89511 -2.00649
C  -7.24202  0.14166 -1.39902
H  -7.29232  0.41472 -2.46578
H  -8.23268 -0.23475 -1.09248
H  -7.02104  1.05558 -0.82378
C  -2.73355  0.83050 -0.74693
N  -2.40982  2.07379 -0.96531
C  -3.45033  3.12567 -1.24220
C  -4.23148  2.80754 -2.53805
H  -4.79712  1.86674 -2.46390
H  -3.53356  2.71812 -3.38938
H  -4.93923  3.62397 -2.76912
C  -4.40820  3.28678 -0.04117
H  -4.97005  2.36107  0.16695
H  -5.13565  4.09702 -0.22788
H  -3.83903  3.54265  0.86972
C  -2.68353  4.44850 -1.45943
H  -2.13512  4.74067 -0.55030
H  -3.37629  5.26700 -1.72515
H  -1.94725  4.33470 -2.27268
```

TS(IV-V)

SCF (BP86) Energy = -2724.65632293
Enthalpy 0K = -2723.377025
Enthalpy 298K = -2723.300669
Free Energy 298K = -2723.481267
Lowest Frequency = -215.6323 cm^{-1}
Second Frequency = 17.1681 cm^{-1}
SCF (BP86-D3BJ) Energy = -2725.05582980
SCF (C6H6) Energy = -2724.66332426
SCF (BS2) Energy = -2924.56254447

```
Mg  0.88970 -0.03642  0.43302
O  -0.05367 -1.66710  1.45794
O  -0.46537 -3.91361  0.89795
O   0.09246 -0.30731 -1.41105
O  -0.44380 -2.42717 -2.25380
O  -4.56102 -0.51291 -1.58405
N   3.03823  0.14234  0.41649
N   0.63799  1.74663  1.60514
C   1.38881  2.95045  3.66328
H   0.61588  2.61741  4.37623
H   1.04537  3.91572  3.25974
H   2.32372  3.11116  4.21919
C   1.58331  1.92208  2.55383
C   2.81216  1.20596  2.62011
H   3.36977  1.37007  3.54538
C   3.53147  0.49387  1.63231
C   4.96256  0.11823  2.00476
H   5.07436 -0.98053  1.99578
H   5.22759  0.48779  3.00620
H   5.69377  0.50879  1.28030
C  -0.44945  2.71158  1.60647
C  -1.56714  2.56916  2.48106
C  -2.55210  3.57643  2.50440
H  -3.40156  3.46852  3.18797
C  -2.46801  4.70636  1.68592
H  -3.23694  5.48501  1.73073
C  -1.38676  4.82537  0.80730
H  -1.31259  5.70800  0.16348
C  -0.37285  3.84874  0.74745
C  -1.76804  1.35810  3.38952
H  -0.85703  0.73849  3.32161
C  -1.98997  1.73674  4.87256
H  -1.19618  2.39307  5.26735
H  -2.02125  0.82707  5.49761
H  -2.95063  2.26207  5.01475
C  -2.95850  0.51206  2.88678
H  -3.88623  1.11064  2.88374
H  -3.12405 -0.35850  3.54412
H  -2.78423  0.14626  1.86287
C   0.81458  4.09332 -0.18208
H   1.33139  3.12710 -0.32094
C   1.81343  5.07791  0.47274
H   1.31334  6.03318  0.71272
H   2.65427  5.30073 -0.20658
H   2.23453  4.67170  1.40678
C   0.38621  4.61020 -1.57251
H  -0.39642  3.97300 -2.01577
H   1.25076  4.62479 -2.25721
H  -0.00492  5.64198 -1.52712
C   4.04090 -0.07561 -0.61225
C   4.43325 -1.38128 -1.02452
C   5.40454 -1.52841 -2.03577
H   5.69499 -2.53856 -2.34562
C   6.01669 -0.42373 -2.63065
H   6.78028 -0.55768 -3.40410
C   4.65344  1.06115 -1.23286
C   5.63774  0.85779 -2.21941
H   6.11442  1.72997 -2.67996
C   3.87572 -2.64871 -0.38873
H   3.18362 -2.31990  0.40714
C   4.99508 -3.48889  0.27088
H   5.68263 -3.90133 -0.48821
```

H 4.56436 -4.34123 0.82408
H 5.60035 -2.89140 0.97375
C 3.08468 -3.51021 -1.40130
H 2.14583 -3.02585 -1.71708
H 2.81207 -4.48354 -0.96042
H 3.69279 -3.71495 -2.30008
C 4.28956 2.50664 -0.87814
H 3.38498 2.47885 -0.24710
C 5.40773 3.20378 -0.06682
H 5.60166 2.69345 0.88999
H 5.12708 4.24709 0.16125
H 6.35378 3.22620 -0.63677
C 3.97097 3.33752 -2.14262
H 4.85490 3.44572 -2.79476
H 3.65077 4.35586 -1.86428
H 3.16933 2.87742 -2.74401
C 0.33500 -0.21917 -2.87301
C -0.27256 -1.60769 -3.42811
C -0.39381 1.03433 -3.37667
H -1.44729 1.04000 -3.06232
H -0.33845 1.10846 -4.47665
H 0.08565 1.93363 -2.95559
C 1.83444 -0.06112 -3.11236
H 2.19905 0.84151 -2.59657
H 2.04896 0.05993 -4.18762
H 2.41419 -0.91589 -2.73857
C 0.66809 -2.35912 -4.38544
H 1.64571 -2.56789 -3.92800
H 0.82856 -1.79414 -5.32026
H 0.20541 -3.32509 -4.64582
C -1.64403 -1.45411 -4.11908
H -1.98371 -2.45178 -4.44209
H -1.60445 -0.80049 -5.00726
H -2.40087 -1.06114 -3.42359
C 0.25336 -2.46916 2.67428
C 0.30293 -3.95728 2.11499
C 1.56704 -1.98221 3.28457
H 2.40374 -2.04458 2.57205
H 1.82643 -2.59208 4.16746
H 1.48032 -0.93381 3.61473
C -0.88961 -2.24786 3.67512
H -0.90682 -1.19307 3.98708
H -0.75867 -2.86560 4.57895
H -1.86590 -2.48875 3.22562
C 1.71488 -4.45134 1.76469
H 1.62554 -5.42377 1.25359
H 2.34464 -4.58721 2.66077
H 2.21283 -3.75728 1.08039
C -0.31661 -4.99623 3.07274
H -1.36006 -4.77313 3.33200
H 0.26799 -5.06711 4.00730
H -0.29448 -5.98574 2.58711
B -0.85621 -2.58760 0.53064
B -0.78729 -1.51538 -1.18281
B -3.30513 -0.21663 -1.03737
O -3.04720 1.15420 -1.04800
C -5.11303 0.72088 -2.15316
C -4.83835 0.69316 -3.66636
H -5.26017 -0.23182 -4.09181
H -5.30611 1.55312 -4.17494
H -3.75896 0.70468 -3.88452
C -6.62590 0.72270 -1.90340
H -7.08127 1.66051 -2.26581
H -7.09161 -0.11396 -2.44973
H -6.86453 0.60675 -0.83591
C -4.30303 1.83778 -1.38218
C -3.96807 3.08733 -2.20252
H -4.88954 3.61616 -2.50251
H -3.36299 3.77431 -1.58876
H -3.39545 2.84139 -3.10939
C -4.96028 2.24275 -0.05246
H -4.25419 2.86308 0.52072
H -5.88419 2.82219 -0.21929
H -5.20788 1.35718 0.55575
C -2.23945 -1.28392 -0.64860
N -2.32555 -2.38046 0.19994
C -3.59348 -3.18262 0.40648
C -4.66508 -2.30773 1.09757
H -4.96375 -1.45069 0.47769
H -4.30156 -1.94284 2.07245
H -5.56651 -2.91947 1.27814
C -4.08821 -3.66792 -0.97593
H -4.33720 -2.81741 -1.62839
H -4.99390 -4.28840 -0.85530
H -3.30985 -4.27626 -1.46546
C -3.33376 -4.41063 1.29594
H -2.53360 -5.04556 0.89268
H -4.26706 -4.99627 1.36421
H -3.05689 -4.11336 2.32047

V

SCF (BP86) Energy = -2724.74894632
Enthalpy 0K = -2723.465002
Enthalpy 298K = -2723.388793
Free Energy 298K = -2723.569512
Lowest Frequency = 14.3021 cm^{-1}
Second Frequency = 21.0976 cm^{-1}
SCF (BP86-D3BJ) Energy = -2725.14839284
SCF (C6H6) Energy = -2724.75516335
SCF (BS2) Energy = -2924.65635783

Mg 0.13891 0.40760 0.66042
O 0.28860 -0.99690 2.27748
O 0.11807 -3.25216 2.83463
O 0.78119 -0.37105 -2.65732
O 1.81407 -2.29862 -1.90278
O -2.83810 -2.08955 -0.61876
N 1.93168 1.55287 0.89964
N -1.16504 2.06657 1.08299
C -1.53860 4.14848 2.41098
H -2.38515 3.80456 3.02653
H -1.97670 4.67793 1.54863
H -0.94918 4.86601 2.99940
C -0.67094 2.98737 1.93772
C 0.66233 3.00037 2.43366
H 0.82651 3.72340 3.23660
C 1.86115 2.45988 1.90177
C 3.14023 3.02871 2.50901
H 3.88335 2.23594 2.68703
H 2.92664 3.54183 3.45858
H 3.61892 3.75434 1.83060
C -2.48764 2.33243 0.54355
C -3.67205 2.05033 1.29381
C -4.92696 2.36418 0.73583
H -5.83189 2.14547 1.31316
C -5.04408 2.94978 -0.52845
H -6.02942 3.19444 -0.93896
C -3.88278 3.22105 -1.25741
H -3.96362 3.69006 -2.24406
C -2.60172 2.92388 -0.74980
C -3.65065 1.41678 2.68526
H -2.60394 1.42468 3.02957
C -4.49446 2.19699 3.72079
H -4.23936 3.26980 3.75253
H -4.33845 1.77919 4.73069
H -5.57395 2.12311 3.50183
C -4.12661 -0.05251 2.63737
H -5.17234 -0.10608 2.28758
H -4.08985 -0.50696 3.64303
H -3.51571 -0.66244 1.95212
C -1.38958 3.32988 -1.58864
H -0.48570 2.94168 -1.08724
C -1.27144 4.87230 -1.64044
H -2.16237 5.32176 -2.11385
H -0.39037 5.17835 -2.23046
H -1.17249 5.30936 -0.63205
C -1.43713 2.72801 -3.01030
H -1.46513 1.62723 -2.97406
H -0.54553 3.03662 -3.58175
H -2.32174 3.08236 -3.56935
C 3.21496 1.48652 0.22721
C 4.17105 0.47213 0.52897
C 5.42941 0.50570 -0.10678
H 6.16557 -0.26791 0.13829

```
C   5.75941  1.49491 -1.03613
H   6.74373  1.50164 -1.51609
C   3.54473  2.49925 -0.72916
C   4.81033  2.47447 -1.34640
H   5.05932  3.25300 -2.07653
C   3.91947 -0.64466  1.54271
H   2.88761 -0.52509  1.91678
C   4.89056 -0.54339  2.74404
H   5.93851 -0.66148  2.41727
H   4.68622 -1.33842  3.48262
H   4.81475  0.42786  3.26233
C   4.02898 -2.04014  0.88621
H   3.29393 -2.16740  0.07599
H   3.85932 -2.83847  1.62930
H   5.03722 -2.20244  0.46500
C   2.58297  3.62652 -1.11824
H   1.65929  3.50585 -0.52807
C   3.15964  5.02811 -0.80924
H   3.41897  5.14692  0.25595
H   2.42144  5.80872 -1.06325
H   4.07163  5.22917 -1.39864
C   2.20678  3.52782 -2.61252
H   3.10136  3.59865 -3.25647
H   1.52910  4.35054 -2.89946
H   1.70467  2.57274 -2.83085
C   1.53637 -0.88517 -3.79740
C   2.53063 -1.91598 -3.12161
C   0.52021 -1.54993 -4.74433
H   0.00531 -2.39226 -4.25533
H   1.00096 -1.91821 -5.66655
H  -0.24368 -0.80568 -5.01912
C   2.21919  0.27896 -4.52136
H   1.45534  0.97056 -4.91378
H   2.81111 -0.09114 -5.37676
H   2.88588  0.84310 -3.85395
C   3.86723 -1.28447 -2.71305
H   3.71943 -0.37649 -2.10992
H   4.47389 -1.02372 -3.59696
H   4.44042 -2.00467 -2.10794
C   2.78921 -3.19098 -3.93610
H   3.45234 -3.86382 -3.36757
H   3.28835 -2.95108 -4.89119
H   1.85920 -3.73666 -4.15513
C   0.27738 -1.07213  3.76902
C   0.60294 -2.59842  4.03099
C   1.29770 -0.09083  4.34410
H   2.31683 -0.28320  3.98161
H   1.30227 -0.17061  5.44518
H   1.02198  0.94354  4.07853
C  -1.13014 -0.68738  4.22750
H  -1.34226  0.34336  3.90790
H  -1.21283 -0.72394  5.32615
H  -1.88962 -1.35464  3.79330
C   2.10632 -2.89806  4.15407
H   2.25012 -3.99064  4.15671
H   2.53091 -2.49123  5.08723
H   2.66488 -2.48431  3.30206
C  -0.13532 -3.20392  5.23421
H  -1.22708 -3.14302  5.11644
H   0.15157 -2.69898  6.17264
H   0.13628 -4.26816  5.32540
B   0.06943 -2.33619  1.77902
B   0.77680 -1.37759 -1.67607
B  -1.74984 -1.36757 -1.13426
O  -2.12215 -0.65187 -2.28157
C  -4.01042 -1.87685 -1.46384
C  -4.65385 -3.24620 -1.73460
H  -4.99265 -3.68584 -0.78189
H  -5.53481 -3.14390 -2.39200
H  -3.95058 -3.95222 -2.20073
C  -5.00217 -1.00467 -0.68295
H  -5.92891 -0.83717 -1.25798
H  -5.27022 -1.51854  0.25430
H  -4.57009 -0.02638 -0.42670
C  -3.40801 -1.15825 -2.74930
C  -3.11886 -2.12559 -3.91393
H  -4.04992 -2.53453 -4.34173
H  -2.58611 -1.57989 -4.70876
H  -2.48177 -2.96745 -3.59861
C  -4.23874  0.01898 -3.27533
H  -3.72513  0.47279 -4.13908
H  -5.23186 -0.32364 -3.61500
H  -4.37400  0.79930 -2.51262
C  -0.30027 -1.48069 -0.55694
N  -0.14977 -2.62172  0.42165
C  -0.29213 -4.09158  0.01136
C  -1.45423 -4.74474  0.80242
H  -2.38794 -4.19648  0.60208
H  -1.26135 -4.74312  1.88474
H  -1.58251 -5.79394  0.48020
C  -0.60381 -4.26876 -1.48728
H  -1.57131 -3.81882 -1.75142
H  -0.67432 -5.35152 -1.68985
H   0.19092 -3.85604 -2.12296
C   1.04313 -4.81782  0.30405
H   1.85135 -4.36881 -0.29487
H   0.96195 -5.88775  0.03960
H   1.30356 -4.75196  1.37168
```

TS(V-P)

XXXX

P_M

SCF (BP86) Energy = -2724.75215188
Enthalpy 0K = -2723.470705
Enthalpy 298K = -2723.393387
Free Energy 298K = -2723.580512
Lowest Frequency = 9.5661 cm^{-1}
Second Frequency = 13.9863 cm^{-1}
SCF (BP86-D3BJ) Energy = -2725.14368553
SCF (C6H6) Energy = -2724.75996990
SCF (BS2) Energy = -2924.66289835

```
Mg -1.12227 -0.07088  0.44852
O  -0.06105 -0.13362 -1.25939
O   1.76243 -0.60681 -2.63295
O   0.18441 -1.09360  1.72046
O   2.30640 -1.85576  2.34789
O   4.02332  1.05188 -0.45093
O   5.78884 -0.42487 -0.80433
N  -1.80868  1.80526  1.13155
N  -3.04033 -0.93806  0.40532
N   3.52533 -1.40445 -0.38298
C  -2.97695  2.80069  3.10715
H  -2.30347  2.68785  3.97685
H  -4.01063  2.77797  3.48462
H  -2.77386  3.78602  2.66496
C  -2.71886  1.66631  2.12081
C  -3.46429  0.47762  2.36165
H  -4.06736  0.50668  3.27441
C  -3.72607 -0.64758  1.53317
C  -4.81664 -1.58253  2.04593
H  -5.16491 -2.27878  1.27110
H  -5.67714 -1.02448  2.44699
H  -4.40266 -2.18408  2.87686
C  -1.34452  3.13947  0.79874
C  -0.11637  3.64120  1.32000
C   0.31334  4.92893  0.94403
H   1.25789  5.30844  1.34913
C  -0.44343  5.73132  0.08676
H  -0.09847  6.73429 -0.18583
C  -1.64849  5.23536 -0.42141
H  -2.24154  5.86113 -1.09598
C  -2.11597  3.94635 -0.09616
C   0.72622  2.85642  2.31831
H   0.24728  1.87314  2.43454
C   0.73350  3.54766  3.70296
H   1.25435  4.52037  3.65396
H   1.26505  2.92547  4.44447
H  -0.28551  3.73768  4.07960
C   2.17032  2.61913  1.83370
H   2.22396  2.04555  0.89530
H   2.73691  2.04962  2.59190
H   2.70342  3.57450  1.68244
```

```
C  -3.62541 -1.83668 -0.56234
C  -3.05688 -3.12547 -0.79245
C  -3.60118 -3.94794 -1.79856
H  -3.16565 -4.93874 -1.96595
C  -4.69574 -3.54155 -2.56640
H  -5.10707 -4.19813 -3.34016
C  -5.26534 -2.28882 -2.32191
H  -6.13304 -1.97112 -2.91054
C  -4.75591 -1.41839 -1.33797
C  -1.94517 -3.70150  0.08339
H  -1.52588 -2.87899  0.68970
C  -2.53444 -4.74840  1.06083
H  -3.33550 -4.31846  1.68555
H  -1.75092 -5.14906  1.72851
H  -2.96710 -5.59909  0.50536
C  -0.79009 -4.31916 -0.72931
H  -1.12982 -5.16408 -1.35307
H  -0.00779 -4.70647 -0.05568
H  -0.31546 -3.57456 -1.38773
C  -5.46644 -0.07389 -1.14652
H  -4.87359  0.53239 -0.44039
C  -6.88184 -0.25843 -0.54608
H  -7.52875 -0.82763 -1.23703
H  -7.35896  0.72222 -0.37290
H  -6.86174 -0.80113  0.41149
C  -5.57981  0.71549 -2.47040
H  -4.59572  0.87944 -2.93696
H  -6.04567  1.69968 -2.28802
H  -6.21413  0.18795 -3.20389
C  -0.29324  0.54273 -2.56811
C   0.65312 -0.29430 -3.50376
C   0.17568  1.99545 -2.41193
H  -0.38859  2.49435 -1.60812
H   0.01262  2.56436 -3.34253
H   1.24727  2.03116 -2.15761
C  -1.77855  0.46146 -2.90663
H  -2.15251 -0.57300 -2.88809
H  -1.95984  0.88183 -3.91088
H  -2.36859  1.05648 -2.18921
C   1.18972  0.48347 -4.71288
H   1.78406  1.35421 -4.39961
H   0.36779  0.82561 -5.36570
H   1.84353 -0.17560 -5.30699
C   0.01962 -1.61878 -3.97249
H   0.80895 -2.24757 -4.41449
H  -0.76182 -1.45604 -4.73423
H  -0.42614 -2.17280 -3.13058
C   0.12798 -1.44170  3.17794
C   1.39536 -2.36602  3.33865
C   0.24329 -0.14784  3.98998
H   1.18912  0.37421  3.77985
H   0.19496 -0.36521  5.06993
H  -0.59956  0.51940  3.74819
C  -1.19718 -2.13059  3.50445
H  -2.03384 -1.42094  3.39314
H  -1.18219 -2.46631  4.55622
H  -1.38279 -3.00703  2.86910
C   2.07573 -2.27241  4.71236
H   2.94893 -2.94458  4.72633
H   1.39356 -2.58408  5.52239
H   2.43408 -1.25315  4.91862
C   1.10396 -3.84545  3.01366
H   0.59501 -3.94710  2.04137
H   0.48352 -4.32552  3.78943
H   2.06122 -4.38710  2.95221
C   2.09812 -1.10573 -0.11653
C   5.13769  1.83334 -0.96517
C   6.37225  0.89660 -0.66706
C   4.89872  2.03831 -2.47428
H   4.87247  1.07139 -3.00204
H   5.67458  2.67474 -2.93360
H   3.92194  2.52987 -2.61553
C   5.17345  3.18911 -0.25150
H   4.26865  3.76798 -0.50103
H   6.05144  3.77703 -0.57225
H   5.21061  3.06960  0.84162
C   7.53833  1.01908 -1.65494
H   8.33385  0.30593 -1.38128
H   7.97062  2.03484 -1.63365
H   7.21967  0.79256 -2.68358
C   6.88540  1.02879  0.78044
H   6.05215  0.95893  1.49870
H   7.41639  1.98201  0.94597
H   7.58525  0.20262  0.98734
C   4.01886 -2.81509 -0.61016
C   2.86292 -3.82613 -0.53786
H   2.37864 -3.81571  0.44971
H   3.26667 -4.83815 -0.71752
H   2.09859 -3.61464 -1.30143
C   4.64572 -2.92621 -2.02394
H   3.89817 -2.64480 -2.78483
H   4.97745 -3.96211 -2.22179
H   5.51492 -2.25966 -2.12513
C   5.06173 -3.17346  0.47635
H   5.91960 -2.48515  0.42811
H   5.43563 -4.20501  0.33880
H   4.60014 -3.09178  1.47428
B   1.32757 -0.64494 -1.30197
B   1.58765 -1.32330  1.26709
B   4.40514 -0.30591 -0.53448
C  -3.43526  3.46855 -0.70878
H  -3.45150  2.36561 -0.63172
C  -4.65863  4.00728  0.07097
H  -4.65789  3.67642  1.12135
H  -5.59888  3.65942 -0.39256
H  -4.66749  5.11186  0.06446
C  -3.56176  3.85057 -2.20077
H  -3.68037  4.93973 -2.33553
H  -4.45143  3.37376 -2.64335
H  -2.67805  3.53610 -2.78004
```

V_{neo}

SCF (BP86) Energy = -2646.11726129
Enthalpy 0K = -2644.886238
Enthalpy 298K = -2644.812490
Free Energy 298K = -2644.990460
Lowest Frequency = 13.6641 cm^{-1}
Second Frequency = 22.8712 cm^{-1}
SCF (BP86-D3BJ) Energy = -2646.49824429
SCF (C6H6) Energy = -2646.12322049
SCF (BS2) Energy = -2846.00896152

```
Mg  0.07882  0.66929 -0.04802
O   0.99912 -2.22856  1.49752
O   1.95937 -2.94890 -0.48135
O  -2.70527 -1.91713 -1.03126
O  -1.83077 -1.88259  1.22932
O   0.04077  0.98037 -2.11179
O   0.43015 -0.15287 -4.23248
N   1.77951  1.82938  0.48425
N  -1.30600  2.06541  0.73643
N  -0.06238 -1.47132 -2.20095
C   2.83429  4.09128  0.54754
H   3.20803  4.18053  1.58238
H   2.57211  5.10210  0.19982
H   3.67027  3.70501 -0.05495
C   1.61781  3.17237  0.49008
C   0.36874  3.84254  0.51211
H   0.44896  4.93269  0.50000
C  -0.93772  3.36205  0.79128
C  -1.92283  4.43206  1.24767
H  -2.95698  4.21581  0.94562
H  -1.62924  5.42091  0.86350
H  -1.92056  4.49311  2.35095
C   3.11035  1.35388  0.79400
C   3.53275  1.30525  2.15813
C   4.83866  0.86604  2.45041
H   5.16377  0.82329  3.49616
C   5.73362  0.50329  1.43684
H   6.75027  0.18043  1.68488
C   5.31191  0.55334  0.10446
H   6.00682  0.26653 -0.69282
C   4.00802  0.96089 -0.24177
C   2.61653  1.71025  3.31657
```

H   1.70213 2.15198 2.88554
C   2.19259 0.47392 4.13941
H   1.66940 -0.26571 3.51225
H   1.52133 0.76931 4.96523
H   3.07245 -0.02206 4.58726
C   3.25977 2.77086 4.23915
H   4.14043 2.36878 4.77035
H   2.53629 3.09973 5.00542
H   3.58983 3.66300 3.68022
C   3.62004 0.99757 -1.71988
H   2.55203 1.26827 -1.76305
C   3.77960 -0.38146 -2.39680
H   3.42142 -0.34113 -3.44037
H   3.20435 -1.16081 -1.87215
H   4.83869 -0.69517 -2.42448
C   4.41578 2.07338 -2.49574
H   5.49917 1.86069 -2.47096
H   4.26544 3.08394 -2.07926
H   4.10828 2.09398 -3.55706
C -2.63767 1.73986 1.19491
C -2.84889 1.42123 2.56906
C -4.15744 1.13422 3.00633
H -4.32419 0.89901 4.06370
C -5.24428 1.14949 2.12438
H -6.25553 0.93552 2.48672
C -5.02386 1.44487 0.77406
H -5.87346 1.46495 0.08198
C -3.73658 1.74488 0.28285
C -1.71013 1.38647 3.59136
H -0.78065 1.66736 3.06578
C -1.51522 -0.03643 4.16419
H -2.43439 -0.39159 4.66390
H -0.70721 -0.04138 4.91572
H -1.25427 -0.75415 3.36962
C -1.93165 2.40529 4.73316
H -2.06816 3.43109 4.35057
H -1.06591 2.40980 5.41830
H -2.82522 2.15294 5.33119
C -3.58985 2.11960 -1.19606
H -2.51367 2.26545 -1.39414
C -4.32159 3.44337 -1.52564
H -5.40897 3.34769 -1.35809
H -4.17342 3.71013 -2.58728
H -3.96283 4.28672 -0.91317
C -4.09202 0.99800 -2.13173
H -3.50647 0.07361 -2.00260
H -4.01295 1.31570 -3.18546
H -5.15555 0.76557 -1.94439
C   1.80432 -3.39607 1.84417
C   2.52225 -3.14155 3.17262
H   3.14660 -2.23752 3.13186
H   3.16356 -4.00049 3.43715
H   1.78060 -3.01221 3.97831
C   0.82830 -4.57887 2.00045
H   0.06999 -4.31417 2.75441
H   1.34421 -5.49743 2.32825
H   0.30225 -4.79194 1.05513
C   2.76029 -3.53605 0.59221
C   4.04832 -2.71218 0.72165
H   4.56681 -2.69813 -0.25061
H   4.73398 -3.14709 1.46893
H   3.83474 -1.67112 1.00664
C   3.09970 -4.97932 0.19812
H   2.19689 -5.56629 -0.02811
H   3.65517 -5.48597 1.00671
H   3.73779 -4.97786 -0.70113
C -0.19791 -1.46993 -0.70187
C -4.01092 -2.25907 -0.53785
H -4.51113 -2.85013 -1.32865
H -4.59292 -1.32713 -0.39307
C -3.98257 -3.04578 0.78781
C -3.12239 -2.21558 1.75995
H -3.65410 -1.28107 2.02166
H -2.95155 -2.77820 2.69784
C -5.41801 -3.14926 1.33928
H -5.86533 -2.15103 1.48964
H -5.42909 -3.67657 2.30968
H -6.06720 -3.71500 0.64743
C -3.38848 -4.45639 0.59741
H -3.96022 -5.02847 -0.15458
H -3.41881 -5.01916 1.54727
H -2.33846 -4.41704 0.26685
C -0.16954 -2.75630 -3.02553
C -0.52780 -3.98373 -2.16450
H   0.23988 -4.18813 -1.40535
H -0.58201 -4.85993 -2.83381
H -1.51015 -3.86558 -1.68673
C -1.30070 -2.59258 -4.07259
H -2.25146 -2.38290 -3.55638
H -1.41415 -3.52410 -4.65595
H -1.08579 -1.77608 -4.77765
C   1.18224 -3.04888 -3.72176
H   1.47409 -2.22558 -4.39002
H   1.10286 -3.97024 -4.32722
H   1.96595 -3.19605 -2.96234
C   0.21009 2.28197 -2.74305
H   1.24066 2.62566 -2.53404
H -0.48349 2.97134 -2.23094
C -0.03447 2.26713 -4.26241
C   0.78672 1.09415 -4.82745
H   0.62165 0.99567 -5.91630
H   1.86952 1.28746 -4.67044
C -1.52730 2.08062 -4.59818
H -1.91209 1.12284 -4.21522
H -1.68036 2.09152 -5.69159
H -2.13191 2.89472 -4.16210
C   0.48171 3.59558 -4.85002
H -0.05785 4.45606 -4.41694
H   0.32681 3.62291 -5.94255
H   1.55939 3.73753 -4.65507
B   0.92555 -2.18630 0.09099
B -1.63113 -1.75465 -0.14680
B   0.14455 -0.24359 -2.87065

P_{neo}
SCF (BP86) Energy = -2646.10319271
Enthalpy 0K = -2644.873532
Enthalpy 298K = -2644.799302
Free Energy 298K = -2644.980859
Lowest Frequency = 10.7020 cm^{-1}
Second Frequency = 15.7044 cm^{-1}
SCF (BP86-D3BJ) Energy = -2646.47541076
SCF (C6H6) Energy = -2646.11101162
SCF (BS2) Energy = -2845.99764858

Mg -0.95865 -0.01132 0.55363
O -0.04884 -1.49204 1.63034
O   2.01577 -2.48565 2.06721
N -0.95976 1.95669 1.31174
N -3.03508 -0.22822 0.56420
N   3.19214 -1.76859 -0.57319
C -1.87071 3.37153 3.16169
H -1.35271 3.13631 4.10902
H -2.89274 3.69232 3.41557
H -1.33336 4.20763 2.69168
C -1.89273 2.13950 2.26548
C -2.95281 1.21969 2.54042
H -3.50461 1.44618 3.45704
C -3.55588 0.22212 1.73160
C -4.85676 -0.37536 2.25408
H -5.64262 -0.37717 1.48382
H -5.22693 0.16103 3.13994
H -4.69162 -1.43326 2.52895
C -0.04402 3.03320 0.97394
C   1.14428 3.27418 1.72528
C   2.03049 4.28221 1.29439
H   2.94457 4.46026 1.87129
C   1.76929 5.05735 0.16280
H   2.47201 5.83544 -0.15338
C   0.58864 4.83563 -0.55347
H   0.36974 5.45573 -1.42799
C -0.32984 3.83926 -0.17114
C   1.50549 2.50851 2.99610
H   0.68208 1.80825 3.20370

C	1.64333	3.44949	4.21705
H	2.50211	4.13354	4.10040
H	1.81541	2.86159	5.13587
H	0.74501	4.07048	4.36971
C	2.80047	1.68557	2.81384
H	2.74211	1.00138	1.95077
H	3.02035	1.09923	3.72429
H	3.66381	2.35425	2.64734
C	-3.91033	-0.89423	-0.37091
C	-3.82246	-2.30057	-0.59454
C	-4.66201	-2.89922	-1.55542
H	-4.58838	-3.98003	-1.72056
C	-5.59681	-2.15558	-2.28032
H	-6.25061	-2.64295	-3.01116
C	-5.68186	-0.77727	-2.05873
H	-6.40883	-0.18660	-2.62744
C	-4.84870	-0.12185	-1.13128
C	-2.88015	-3.20532	0.19543
H	-2.31706	-2.56677	0.89517
C	-3.67334	-4.23850	1.02977
H	-4.39047	-3.74980	1.71137
H	-2.99080	-4.86024	1.63616
H	-4.24734	-4.92206	0.37998
C	-1.85414	-3.91361	-0.71528
H	-2.35152	-4.52962	-1.48507
H	-1.20619	-4.58405	-0.12466
H	-1.20297	-3.18627	-1.22475
C	-4.97433	1.40078	-1.00979
H	-4.17240	1.75620	-0.34062
C	-6.32843	1.83530	-0.40078
H	-7.17249	1.49891	-1.02864
H	-6.38320	2.93574	-0.32828
H	-6.47970	1.42543	0.61069
C	-4.78337	2.08140	-2.38486
H	-3.82317	1.79795	-2.84660
H	-4.80600	3.18010	-2.28059
H	-5.58623	1.80448	-3.09006
C	-0.05690	-1.91182	3.06236
C	1.11387	-2.96863	3.08275
C	0.24598	-0.67912	3.91798
H	1.23289	-0.26303	3.67138
H	0.22975	-0.93341	4.99086
H	-0.52936	0.08716	3.74703
C	-1.43073	-2.46609	3.44029
H	-2.19733	-1.67753	3.35427
H	-1.41298	-2.79838	4.49296
H	-1.72761	-3.32015	2.81795
C	1.87758	-3.03753	4.41291
H	2.67735	-3.79114	4.33021
H	1.21337	-3.33523	5.24294
H	2.34837	-2.07443	4.65941
C	0.65036	-4.38357	2.68046
H	0.08195	-4.36231	1.73626
H	0.02621	-4.85314	3.45988
H	1.54056	-5.01349	2.52451
C	1.77144	-1.44472	-0.29523
C	3.58641	-3.16281	-1.00297
C	2.38389	-4.12662	-0.96667
H	1.96790	-4.21639	0.04804
H	2.72706	-5.12514	-1.29090
H	1.57727	-3.80038	-1.64200
C	4.12242	-3.14245	-2.45810
H	3.34894	-2.74106	-3.13363
H	4.38737	-4.16343	-2.78995
H	5.01774	-2.50854	-2.53661
C	4.66425	-3.70879	-0.03358
H	5.56135	-3.07197	-0.05047
H	4.96091	-4.73594	-0.31619
H	4.26243	-3.72533	0.99311
B	1.29728	-1.79782	1.07607
C	-1.64497	3.71302	-0.94064
H	-2.00984	2.67650	-0.81459
C	-2.71227	4.65399	-0.32958
H	-2.91226	4.41197	0.72657
H	-3.66596	4.58062	-0.88099
H	-2.37197	5.70364	-0.37732
C	-1.49749	3.99489	-2.45088
H	-1.30395	5.06300	-2.65270
H	-2.42845	3.73434	-2.98069
H	-0.66941	3.41774	-2.89720
O	3.67033	0.61094	-0.29748
O	5.46395	-0.91399	-0.94088
C	4.58596	1.70415	-0.35324
H	5.04987	1.85558	0.64613
H	3.99857	2.61436	-0.58096
C	5.70577	1.50595	-1.39565
C	6.38471	0.17045	-1.03060
H	7.14301	-0.09866	-1.79219
H	6.91840	0.28652	-0.06212
C	5.11598	1.45111	-2.82077
H	4.35483	0.66022	-2.91425
H	5.90870	1.25625	-3.56497
H	4.63762	2.41320	-3.07765
C	6.72830	2.65094	-1.28229
H	6.25683	3.62455	-1.50570
H	7.55826	2.51240	-1.99793
H	7.16303	2.70986	-0.26809
B	4.11948	-0.68725	-0.59497
O	-0.34867	-0.39286	-1.31741
O	1.62983	-0.70129	-2.72305
C	-1.13419	0.04529	-2.46811
H	-1.14937	1.15180	-2.46277
H	-2.16418	-0.32152	-2.31394
C	-0.56479	-0.44742	-3.80883
C	0.93465	-0.08836	-3.80176
H	1.41370	-0.42702	-4.73983
H	1.04845	1.01684	-3.75197
C	-0.75493	-1.96827	-3.98221
H	-0.19181	-2.53561	-3.22422
H	-0.39027	-2.28892	-4.97377
H	-1.82031	-2.24485	-3.90151
C	-1.28199	0.30877	-4.94450
H	-2.36679	0.10282	-4.93579
H	-0.89134	-0.00994	-5.92645
H	-1.14092	1.40116	-4.86208
B	1.04730	-0.84108	-1.46248

References

1. A. F. Pécharman, A. L. Colebatch, M. S. Hill, C. L. McMullin, M. F. Mahon, C. Weetman, *Nature Commun.* **2017**, *8*, 15022
2. H. T. W. Shere, M. S. Hill, S. E. Neale, M. F. Mahon, C. L. McMullin, A. S. S. Wilson, *Z. Anorg. Allg. Chem.* **2023**, *649*, e202200376.
3. A. F. Pécharman, M. S. Hill, M. F. Mahon, *Dalton Trans*., **2018**,*47*, 7300-7305.
4. O. V. Dolomanov, L. J. Bourhis, R. J. Gildea, J. A. K. Howard and H. Puschmann, *J. Appl. Cryst.* 2009, **42**, 339-341.
5. G. M. Sheldrick, *Acta Cryst*. 2015, **A71**, 3-8.
6. G. M. Sheldrick, *Acta Cryst*. 2015, **C71**, 3-8.
7. M. J. Frisch, G. W. Trucks, H. B. Schlegel, G. E. Scuseria, M. A. Robb, J. R. Cheeseman, G. Scalmani, V. Barone, G. A. Petersson, H. Nakatsuji, X. Li, M. Caricato, A. V. Marenich, J. Bloino, B. G. Janesko, R. Gomperts, B. Mennucci, H. P. Hratchian, J. V. Ortiz, A. F. Izmaylov, J. L. Sonnenberg, Williams, F. Ding, F. Lipparini, F. Egidi, J. Goings, B. Peng, A. Petrone, T. Henderson, D. Ranasinghe, V. G. Zakrzewski, J. Gao, N. Rega, G. Zheng, W. Liang, M. Hada, M. Ehara, K. Toyota, R. Fukuda, J. Hasegawa, M. ishida, T. Nakajima, Y. Honda, O. Kitao, H. Nakai, T. Vreven, K. Throssell, J. A. Montgomery Jr., J. E. Peralta, F. Ogliaro, M. J. Bearpark, J. J. Heyd, E. N. Brothers, K. N. Kudin, V. N. Staroverov, T. A. Keith, R. Kobayashi, J. Normand, K. Raghavachari, A. P. Rendell, J. C. Burant, S. S. Iyengar, J. Tomasi, M. Cossi, J. M. Millam, M. Klene, C. Adamo, R. Cammi, J. W. Ochterski, R. L. Martin, K. Morokuma, O. Farkas, J. B. Foresman, D. J. Fox, Wallingford, CT, 2016.
8. D. Andrae, U. Häußermann, M. Dolg, H. Stoll, H. Preuß, *Theor. Chim. Acta* 1990, 77, 123-141.
9. (a) P. C. Hariharan, J. A. Pople, *Theor. Chim. Acta* 1973, 28, 213-222; (b) W. J. Hehre, R. Ditchfield, J. A. Pople, *J. Chem. Phys.* 1972, 56, 2257-2261.
10. (a) A. D. Becke, *Phys. Rev. A* 1988, 38, 3098-3100; (b) J. P. Perdew, *Phys. Rev. B* 1986, 33, 8822-8824.
11. J. Tomasi, B. Mennucci, R. Cammi, *Chem. Rev.* 2005, 105, 2999-3094.
12. S. Grimme, S. Ehrlich, L. Goerigk, *J. Comp. Chem.* 2011, 32, 1456-1465.
